# Low-dose mRNA-1273 COVID-19 vaccine generates durable memory enhanced by cross-reactive T cells

**DOI:** 10.1126/science.abj9853

**Published:** 2021-10-22

**Authors:** Jose Mateus, Jennifer M. Dan, Zeli Zhang, Carolyn Rydyznski Moderbacher, Marshall Lammers, Benjamin Goodwin, Alessandro Sette, Shane Crotty, Daniela Weiskopf

**Affiliations:** 1 Center for Infectious Disease and Vaccine Research, La Jolla Institute for Immunology (LJI), La Jolla, CA 92037, USA.; 2 Department of Medicine, Division of Infectious Diseases and Global Public Health, University of California, San Diego (UCSD), La Jolla, CA 92037, USA.

## Abstract

Vaccine-specific CD4^+^ T cell, CD8^+^ T cell, binding antibody, and neutralizing antibody responses to the 25-μg Moderna messenger RNA (mRNA)–1273 vaccine were examined over the course of 7 months after immunization, including in multiple age groups, with a particular interest in assessing whether preexisting cross-reactive T cell memory affects vaccine-generated immunity. Vaccine-generated spike-specific memory CD4^+^ T cells 6 months after the second dose of the vaccine were comparable in quantity and quality to COVID-19 cases, including the presence of T follicular helper cells and interferon-γ–expressing cells. Spike-specific CD8^+^ T cells were generated in 88% of subjects, with equivalent memory at 6 months post-boost compared with COVID-19 cases. Lastly, subjects with preexisting cross-reactive CD4^+^ T cell memory exhibited stronger CD4^+^ T cell and antibody responses to the vaccine, demonstrating the biological relevance of severe acute respiratory syndrome coronavirus 2–cross-reactive CD4^+^ T cells.

## A smaller-dose jab does the job

Low-dose messenger RNA (mRNA) vaccines potentially allow health providers to administer more doses from a limited vaccine supply and can be less reactogenic. Whether low-dose COVID-19 mRNA vaccines generate immune responses comparable to currently approved doses remains an open question, however. Mateus *et al*. report the results of a clinical trial comparing patients who received a 25-μg mRNA-1273 (Moderna) COVID-19 vaccine to 100-μg mRNA-1273 COVID-19 vaccinees and severe acute respiratory syndrome coronavirus 2–infected individuals. The low-dose Moderna vaccine generated long-lived T cell immunity that was equivalent between younger and older patients and that could be enhanced by the presence of cross-reactive T cells. Moreover, antibody and T cell responses induced by the low-dose vaccine were comparable to natural infection and about half as strong as those seen with high-dose vaccination. —STS

## Abstract (structured)

**INTRODUCTION:**

Understanding human immune responses to severe acute respiratory syndrome coronavirus 2 (SARS-CoV-2) RNA vaccines is of interest for a panoply of reasons. mRNA vaccines have demonstrated impressive protection against COVID-19, but the durability of immunity has been a major unknown. Moreover, a better understanding of age-associated differences and mRNA vaccine dose response curves for dose sparing considerations is needed. Additionally, the impact of preexisting cross-reactive memory on immune responses to SARS-CoV-2 proteins remains an open question. Cross-reactive memory CD4^+^ T cells recognizing SARS-CoV-2 have been found in ~50% of individuals. A vaccine trial is a controlled context for testing the relevance of such cross-reactive T cells. Each of these topics was addressed in this study using blood samples from a National Institutes of Health clinical trial of 25-µg mRNA-1273 COVID-19 vaccinees as well as from 100-µg mRNA-1273 COVID-19 vaccinees and SARS-CoV-2–infected individuals.

**RATIONALE:**

Vaccination and infection are two different paths to immunity. Comparison of vaccine-generated and infection-generated immune memory is of value. Given evidence that antibodies, CD4^+^ T cells, and CD8^+^ T cells can each participate in protective immunity against COVID-19, we measured acute and memory SARS-CoV-2 spike–specific antibodies, CD4^+^ T cells, and CD8^+^ T cells in the blood of subjects who received a low-dose (25 µg) or standard-dose (100 µg) mRNA-1273 COVID-19 vaccine. Immunological measurements were used to address the four issues described above: namely, the durability of immune memory over 7 months after vaccination, mRNA vaccine dose responses, age differences, and the impact of preexisting cross-reactive T cells.

**RESULTS:**

Longitudinal samples from 35 volunteers immunized with 25 µg of mRNA-1273 on days 1 and 29 were used to measure SARS-CoV-2 spike–binding antibodies, receptor binding domain (RBD)–binding antibodies, SARS-CoV-2 pseudovirus (PSV) neutralizing antibodies, spike-specific CD4^+^ T cells, and spike-specific CD8^+^ T cells. Overall, substantial anti-spike, anti-RBD, and PSV neutralizing antibodies were induced in response to two 25-µg mRNA-1273 vaccinations, were maintained in 88 to 100% of vaccinees for at least 6 months after the second immunization, and were comparable in magnitude and quality to those observed 6 to 7 months after infection with SARS-CoV-2.

Spike-specific CD4^+^ T cells were generated by low-dose mRNA-1273 and were maintained as memory CD4^+^ T cells. We observed strong T follicular helper (T_FH_) and type 1 T helper cell polarization of these cells, which is advantageous for antiviral immunity. Spike-specific CD8^+^ T cells were detectable in 88% of vaccinees and maintained for at least 6 months in 67% of vaccinees. Spike-specific CD4^+^ or CD8^+^ T cell frequencies were not lower in older vaccinee groups than in 18- to 55-year-olds, either in the acute or memory phase. Thus, 25-µg mRNA-1273 vaccination induced spike antibody levels and memory T cell frequencies at 7 months after vaccination similar to those observed for COVID-19 cases 7 months after symptom onset.

Next, to assess the impact of mRNA dosing, we compared immune responses between 25-µg and 100-µg doses of mRNA-1273 vaccine. Peak anti-spike, anti-RBD, and PSV neutralizing antibody levels were about twofold higher in 100-µg vaccinees than in 25-µg vaccinees. Spike-specific CD4^+^ T cells responses were ~1.4-to-2.0-fold higher in 100-µg vaccinees, whereas peak CD8^+^ T cell responses were comparable between 25-µg and 100-µg dose regimens.

Finally, to address potential positive or negative effects of preexisting cross-reactive memory T cells, we compared 25-µg mRNA-1273 COVID-19 vaccine responses between subjects with or without measurable preexisting SARS-CoV-2 spike–reactive memory CD4^+^ T cells. Preexisting immunity enhanced vaccine antibody responses after a single vaccine dose, which was associated with higher spike-specific T_FH_ cells and total spike-specific CD4^+^ T cell responses. Individuals with preexisting cross-reactive memory T cells also sustained higher SARS-CoV-2–neutralizing antibodies 6 months after vaccination.

**CONCLUSION:**

The 25-µg dose of mRNA-1273 vaccine induces durable and functional T cell and antibody memory at comparable magnitude to natural infection. This work expands our understanding of immune memory to mRNA vaccine in humans, vaccine dose sparing, and possible timing of boosters. Finally, these data provide evidence that cross-reactive memory CD4^+ ^T cells are biologically relevant and can exert a considerable positive influence on immunity generated by vaccination, with potential implications for vaccines and SARS-CoV-2 infections.

## Teaser

A reduced dose of the Moderna SARS-CoV-2 vaccine induces long-lived T cell and antibody responses comparable to natural infection.

## Graphical abstract

**Figure:**
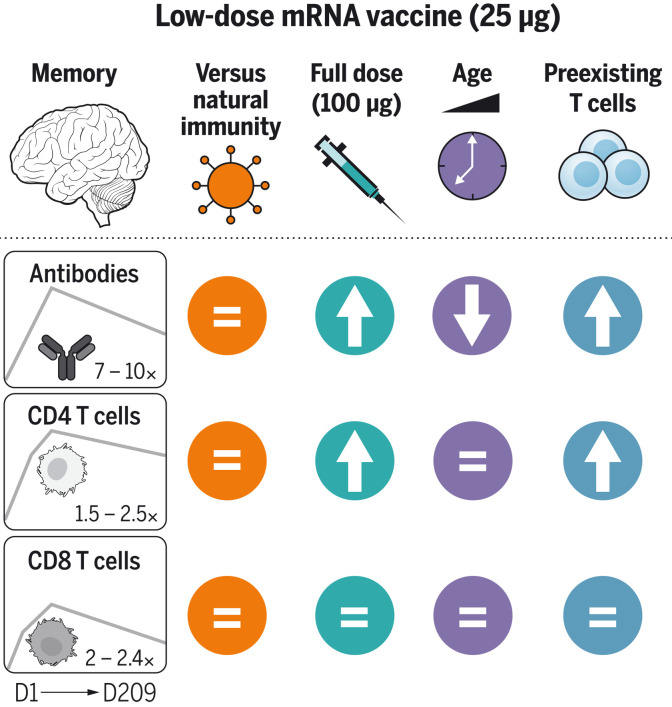
Response to low-dose mRNA-1273 vaccination over 7 months. Immunological memory of antibodies, CD4^+^ T cells, and CD8^+^ T cells was examined after low-dose mRNA vaccination. Levels of spike-specific immune memory were then compared to immune memory observed after natural infection with SARS-CoV-2 or after full-dose vaccination. Robust immune memory comparable to natural infection but lower than after full-dose vaccination was observed. Increased vaccinee age correlated with reduced antibody levels but had no effect on cellular immune memory. Immune memory was enhanced by preexisting cross-reactive T cells. D1, day 1.

Understanding human immune responses to severe acute respiratory syndrome coronavirus 2 (SARS-CoV-2) RNA vaccines is of interest for a panoply of reasons. mRNA vaccines have demonstrated impressive protection against COVID-19 (*1*–*7*). The COVID-19 vaccine mRNA-1273, developed as a collaboration between Moderna and the National Institutes of Health Vaccine Research Center, encodes a stabilized SARS-CoV-2 full-length spike (*8*, *9*). Durability of immunity has been, and remains, a major unknown for mRNA vaccines in humans. Encouraging reports from both Pfizer-BioNTech and Moderna indicate protective immunity of 91 and 93%, respectively, over the 6-month period after the second immunization (7 months after the first immunization) (*10*, *11*), down modestly from the 95% maximal protection observed for each of those two vaccines within 1 to 2 months after the second immunization (*1*, *2*). Although neutralizing antibodies are a clear correlate of immunity after two immunizations (*12*), the underlying mechanisms remain unclear. Moreover, those mechanisms of immunity may change as the immune response develops (e.g., after a single immunization) or as immune memory changes composition (*13*–*15*). Direct measurements of immune memory compartments in humans are necessary to provide insights into these important topics.

Infection and vaccination are two different paths to immunity. Comparison of vaccine-generated immune memory with immune memory of persons infected with SARS-CoV-2 is of value, as studies have indicated that natural immunity is 93 to 100% protective against symptomatic reinfection for 7 to 8 months (*16*–*19*), although natural immunity protection against certain variants of concern (VOCs) is likely to be lower (*20*). After SARS-CoV-2 infection, immunological memory has been observed for ≥8 months for CD4^+^ T cells, CD8^+^ T cells, memory B cells, and antibodies (*21*, *22*). The immune memory in response to SARS-CoV-2 infection exhibits a relatively gradual decline that partially stabilizes within 1 year (*23*–*26*). The 100-μg mRNA-1273 vaccination has been shown to induce durable antibody responses (*27*), but it is unknown whether immune memory to the mRNA-1273 vaccine months after immunization is similar to or different than memory generated by SARS-CoV-2 infection. Additionally, both 25-μg- and 100-μg-dose mRNA-1273 vaccinations have been tested in clinical trials (*9*, *28*), with 100-μg mRNA-1273 proceeding toward licensure (*2*, *29*).

Preexisting cross-reactive memory CD4^+^ T cells that recognize SARS-CoV-2 have been found in ~50% of individuals pre-pandemic (*30*–*37*). There has been intense interest in understanding whether these preexisting cross-reactive memory CD4^+^ T cells, identified in vitro, are biologically relevant in vivo (*33*, *38*, *39*)*.* One approach to test the relevance of such T cells in a controlled fashion is in the context of a vaccine trial, as individuals in a clinical trial are all exposed to a well-defined dose of antigen at a specific time. Additionally, exposure to a low antigen dose may be more sensitive to influence by cross-reactive memory. Thus, we examined immune responses to the 25-μg dose of the mRNA-1273 COVID-19 vaccine.

## Spike-specific antibody elicited by the 25-μg mRNA-1273 vaccine dose over time

**Fig. 1.**
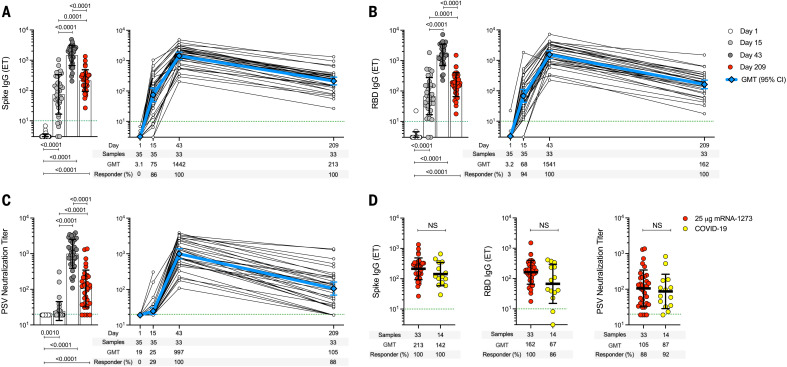
Spike antibodies induced by 25-μg mRNA-1273 vaccination. Participants received two injections of the 25-μg mRNA-1273 vaccine, 28 days apart. PBMC samples were collected on day 1, day 15 ± 2 (2 weeks after first dose), day 43 ± 2 (2 weeks after second dose), and day 209 ± 7 days (6 months after second dose). (**A**) Longitudinal anti-SARS-CoV-2 spike IgG binding titers, (**B**) longitudinal anti-SARS-CoV-2 RBD IgG binding titers, and (**C**) longitudinal SARS-CoV-2 spike pseudovirus neutralizing titers (PSV). (**D**) Comparison of anti-spike IgG, anti-RBD IgG, and PSV neutralizing titers induced by two doses of 25-μg mRNA-1273 vaccine at day 209 ± 7 (*n* = 33) and COVID-19 convalescent donors at 170 to 195 days PSO (*n* = 14). Dotted green lines indicate the limit of quantification (LOQ). The bars in (A), (B), (C), and (D) indicate the geometric mean titers (GMTs) and geometric SD for anti-spike IgG (endpoint titer, ET), anti-RBD IgG (ET), and PSV neutralizing titers, respectively. Data were analyzed for statistical significance using Wilcoxon signed-rank test [(A), (B), and (C)] and Mann-Whitney *U* test (D). NS, nonsignificant. Background-subtracted and log data analyzed in all cases.

An open-label, age de-escalation phase 1 trial used the mRNA-1273 vaccine with 25-μg immunizations on days 1 and 29 (*9*, *28*), with blood samples collected on study days 1, 15, 43, and 209. SARS-CoV-2 spike–binding antibodies, receptor binding domain (RBD)–binding antibodies, and SARS-CoV-2 pseudovirus (PSV) neutralization titers were determined (Fig. 1). Anti-spike and anti-RBD immunoglobulin G (IgG) were maintained at detectable levels for at least 7 months after the first vaccination in 100% (33/33) of subjects (Fig. 1, A and B). RBD IgG was induced by one immunization in 94% (33/35) of subjects. This response rate increased to 100% (33/33) after the second immunization and was maintained for at least 6 months after the second vaccination. SARS-CoV-2 PSV neutralization titers were detected in 29% (10/35) of subjects after one vaccination and 100% of subjects after two vaccinations (33/33), and 88% (29/33) of subjects maintained detectable neutralizing antibodies for at least 6 months after the second vaccination (Fig. 1C). All three antibody measurements demonstrated similar kinetics (Fig. 1, A to C) and were highly correlated (correlation coefficient, *r* = 0.89 to 0.90, fig. S1). Peak spike IgG, RBD IgG, and PSV titers were 6.8-, 9.5-, and 9.5-fold higher, respectively, than at 7 months (study day 209; 181 days after the second immunization). Similar fold changes were reported for 100-μg mRNA-1273 vaccination, indicating similar memory quality and durability (*40*). The 25-μg mRNA-1273 vaccine–generated antibodies were comparable to antibodies from SARS-CoV-2–infected subjects collected at a similar time after exposure [7 months post–symptom onset (PSO), 170 to 195 days] (Fig. 1D). Thus, increased anti-spike IgG, anti-RBD IgG, and PSV neutralizing antibodies were induced in response to two 25-μg mRNA-1273 vaccinations. These levels were maintained in 88 to 100% of vaccinees for at least 6 months after the second immunization and were comparable to those observed after infection with SARS-CoV-2.

## Spike-specific CD4^+^ T cells elicited by the 25-μg mRNA-1273 vaccine dose over time

**Fig. 2.**
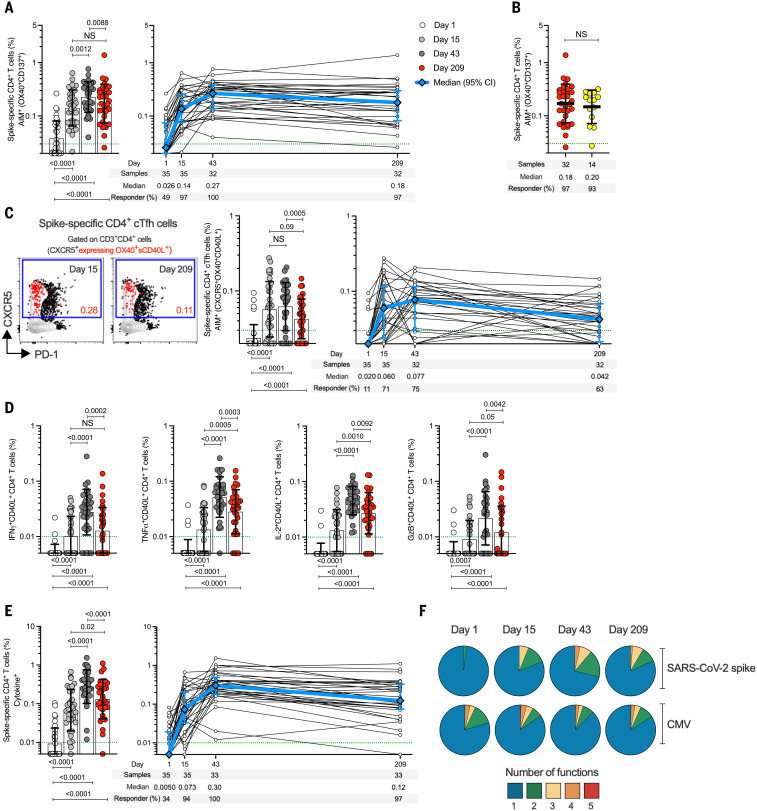
mRNA-1273 vaccination induces durable and multifunctional spike-specific CD4^+^ T cell responses. (**A**) Longitudinal spike-specific CD4^+^ T cells in mRNA-1273 vaccinees measured by AIM. Spike-specific CD4^+^ T cells quantified by AIM (surface OX40^+^CD137^+^) after stimulation with spike megapool (MP) in mRNA-1273 vaccinees (see fig. S2 for gating strategy). (**B**) Comparison of spike-specific AIM^+^ CD4^+^ T cell frequencies between 25-μg mRNA-1273 vaccine at day 209 ± 7 (red circles, *n* = 32) and COVID-19 convalescent donors at 170 to 195 days PSO (yellow circles, *n* = 14). (**C**) Quantitation of spike-specific circulating T follicular helper (cT_FH_) cells (CXCR5^+^OX40^+^surface CD40L^+^, as percentage of CD4^+^ T cells) after stimulation with spike MP. Representative examples of spike-specific cT_FH_ cells (red), overlaid on total CD4^+^ T cells, at days 15 ± 2 and 209 ± 7. (**D**) Spike-specific CD4^+^ T cells expressing intracellular CD40L (iCD40L) and producing IFNγ, TNFα, IL-2, or granzyme B (GzB) in mRNA-1273 vaccinees. (**E**) Longitudinal spike-specific CD4^+^ cytokine^+^ T cells expressing iCD40L or producing IFNγ, TNFα, IL-2, or GzB in 25-μg mRNA-1273 vaccinees (see fig. S4 for gating strategy). Dotted green lines indicate the limit of quantification (LOQ). White, day 1; light gray, day 15 ± 2; dark gray, day 43 ± 2; red, day 209 ± 7. The bars in (A) to (E) indicate the geometric mean and geometric SD in the analysis of the spike-specific CD4^+^ T cell frequencies. (**F**) Longitudinal multifunctional spike-specific CD4^+^ T cells in mRNA-1273 vaccinees. Proportions of multifunctional activity profiles of the spike-specific CD4^+^ T cells from mRNA-1273 vaccinees evaluated on days 1, 15 ± 2, 43 ± 2, and 209 ± 7. The blue, green, yellow, orange, and red colors in the pie charts depict the production of one, two, three, four, or five functions, respectively (see figs. S4 and S6 for details). Data were analyzed for statistical significance using Wilcoxon signed-rank test [(A), (C), (D), and (E)] and Mann-Whitney *U* test (B). Background-subtracted and log data analyzed in all cases.

SARS-CoV-2 spike–specific CD4^+^ T cell responses were first measured using a flow cytometry activation-induced marker (AIM) assay (Fig. 2A and fig. S2). On day 1, before vaccination, spike-specific CD4^+^ T cells with a predominantly memory phenotype were detected in 49% of clinical trial subjects (17/35), demonstrating the presence of preexisting SARS-CoV-2 spike–cross-reactive memory CD4^+^ T cells, as discussed in the latter part of this Report. Spike-specific CD4^+^ T cell responses were observed after the first vaccination in 97% of subjects (34/35) (Fig. 2A). Cytomegalovirus (CMV)–specific CD4^+^ T cells were unchanged, as expected, indicating no bystander influence of the mRNA-1273 vaccination (fig. S3). The SARS-CoV-2 spike–specific CD4^+^ T cell response rate increased to 100% (32/32) after the second vaccination and was maintained for at least 6 more months. Spike-specific memory CD4^+^ T cell frequencies at 7 months were similar to those observed for COVID-19 cases (COVID-19 samples collected 170 to 195 days PSO) (Fig. 2B). Median mRNA-1273 vaccine–generated spike-specific CD4^+^ T cell frequencies at all time points after vaccination also exceeded CMV-specific CD4^+^ T cell frequencies (Fig. 2A and fig. S3A).

T follicular helper (T_FH_) cell differentiation and cytokine production by vaccine-generated spike-specific CD4^+^ T cells were then assessed (Fig. 2, C to F). T_FH_ cells are the specialized subset of CD4^+^ T cells required for B cell help and are critical for the generation of neutralizing antibodies in most conditions (*41*). Spike-specific circulating T_FH_ (cT_FH_) cells were detected in 71% (25/35) and 75% (24/32) of subjects after the first and second vaccination, respectively (Fig. 2C, right panel). Spike-specific cT_FH_ cells were detectable in 94% of subjects overall (33/35). Different response kinetics were observed at the level of individual subjects (Fig. 2C, right panel). Spike-specific memory cT_FH_ cells were still detected in 63% of vaccinees 6 months after the second vaccination (20/32) (Fig. 2C). Vaccine-specific CD4^+^ T cell cytokine profiles were determined by intracellular cytokine staining (ICS) (Fig. 2, D to F, and fig. S4). Interferon-γ positive (IFNγ^+^) spike-specific CD4^+^ T cells were detected in 85% (28/33), tumor necrosis factor–α positive (TNFα^+^) in 97% (32/33), interleukin-2 positive (IL-2^+^) in 100% (33/33), and granzyme B positive (GzB^+^) in 76% (25/33) of subjects at day 43 (Fig. 2D). Little-to-no IL-4, IL-17A, or IL-10 was detected (fig. S5). Cytokine-producing spike-specific CD4^+^ T cells (CD40L^+^ cells producing IFNγ, TNFα, IL-2, and/or GzB) (Fig. 2E and fig. S6) were observed in 94% (33/35) and 100% (33/33) of subjects after the first and second vaccination, respectively, and were maintained for at least 6 months after the second vaccination [97% (32/33)] (Fig. 2E). Spike-specific CD4^+^ T cells generated by the 25-μg mRNA-1273 vaccine exhibited multifunctionality comparable with that of CMV-specific cells (Fig. 2F, fig. S3C, and table S1). Thus, robust spike-specific CD4^+^ T cells and T cell memory were generated by the low-dose mRNA-1723 vaccine, with strong T_FH_ and T helper 1 cell polarization advantageous for antiviral immunity.

## Spike-specific CD8^+^ T cells elicited by the 25-μg mRNA-1273 vaccine dose over time

**Fig. 3.**
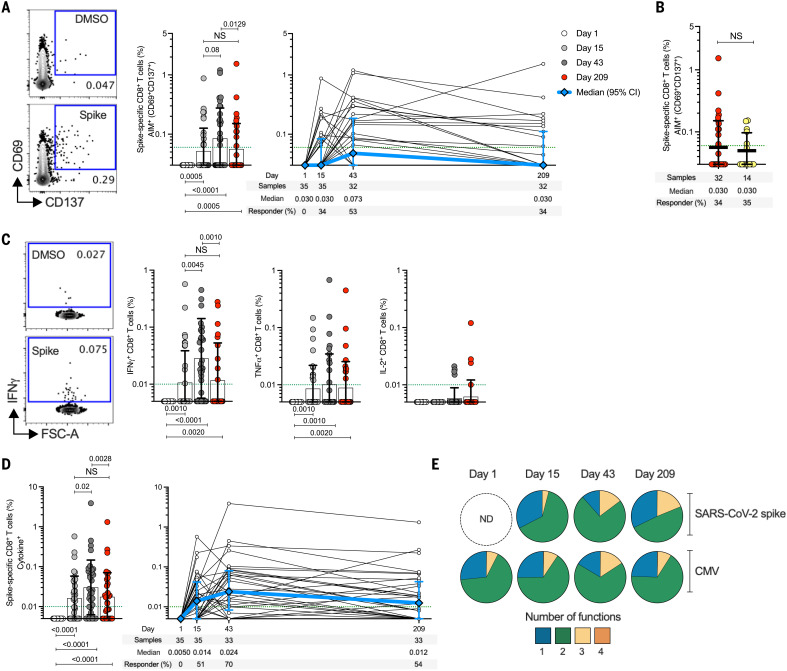
mRNA-1273 vaccination induces multifunctional spike-specific CD8^+^ T cells. (**A**) Longitudinal spike-specific CD8^+^ T cells in mRNA-1273 vaccinees measured by AIM (surface CD69^+^CD137^+^). (Left panel) Representative examples of flow cytometry plots of spike-specific CD8^+^ T cells compared with DMSO control (see fig. S2 for gating strategy). (Right panel) Spike-specific CD8^+^ T cells quantified. (**B**) Comparison of spike-specific AIM^+^ CD8^+^ T cell frequencies between 25-μg mRNA-1273 vaccinees at day 209 ± 7 (*n* = 32) and COVID-19 convalescent donors at 170 to 195 days PSO (*n* = 14). (**C**) Spike-specific CD8^+^ T cells producing IFNγ, TNFα, or IL-2 by intracellular cytokine staining (ICS) in 25-μg mRNA-1273 vaccinees. (**D**) Longitudinal spike-specific CD8^+^ cytokine^+^ T cells producing IFNγ, TNFα, IL-2, or GzB in 25-μg mRNA-1273 vaccinees (see fig. S4 for gating strategy). Dotted green lines indicate the LOQ. The bars in (A) to (D) indicate the geometric mean and geometric SD. White, day 1; light gray, day 15 ± 2; dark gray, day 43 ± 2; red, day 209 ± 7. (**E**) Multifunctional activity profiles of spike-specific CD8^+^ T cells from 25-μg mRNA-1273 vaccinees, evaluated for IFNγ, TNFα, IL-2, or GzB (see figs. S4 and S8 for details). The blue, green, yellow, and orange colors in the pie charts depict the production of one, two, three, or four functions, respectively. ND, nondetectable. Data were analyzed for statistical significance using Wilcoxon signed-rank test [(A), (C), and (D)] and Mann-Whitney *U* test (B). Background-subtracted and log data analyzed in all cases.

SARS-CoV-2 spike–specific CD8^+^ T cells were measured by AIM (CD69^+^ and CD137^+^) (fig. S2) and were observed in 34% (12/35) and 53% (17/32) of subjects after the first and second 25-μg mRNA-1273 vaccination, respectively (Fig. 3A). Spike-specific CD8^+^ T cells were detectable for >6 months after the second vaccination, with a response rate comparable to that observed for COVID-19 cases (COVID-19 samples collected 170 to 195 days PSO) (Fig. 3B). Next, SARS-CoV-2 spike–specific CD8^+^ T cells were measured by ICS (IFNγ, TNFα, IL-2, or GzB) (Fig. 3C and fig. S4). The first immunization induced significant spike-specific CD8^+^ T cell cytokine responses in 51% (18/35) of subjects (Fig. 3D and fig. S7), increasing to 70% (23/33) of subjects after the second vaccination (Fig. 3D). IFNγ^+^ spike-specific CD8^+^ T cells were detected in 70% (23/33), TNFα in 39% (13/33), and IL-2^+^ in 12% (4/33) of vaccinees at day 43 (Fig. 3C). Multiple positive- and negative-control samples and experimental conditions were used to demonstrate the specificity of the spike CD8^+^ T cells (Fig. 3 and figs. S2 and S4, F and G). Correlation between AIM and ICS methods was highly significant for both spike-specific CD4^+^ and CD8^+^ T cells (*P* < 0.0001) (fig. S8). The fraction of multifunctional spike-specific CD8^+^ T cells increased between first and second vaccination (three or more effector molecules expressed) (Fig. 3E and table S2). The most prevalent profile of CD8^+^ T cells with three functions was GzB^+^IFNγ^+^TNFα^+^ (fig. S7), similar to the profile seen in CMV-specific CD8^+^ T cells (Fig. 3D and fig. S9). Thus, 25-μg mRNA-1273 vaccination induces multifunctional spike-specific memory CD8^+^ T cells.

**Fig. 4.**
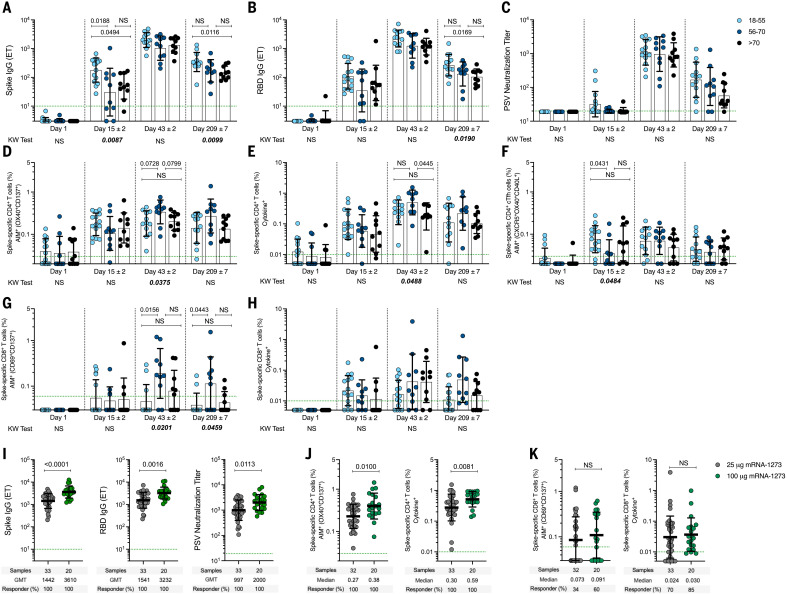
Spike-specific antibody and T cell responses induced by mRNA-1273 vaccination. (**A** to **H**) Immune responses to 25-μg mRNA-1273 vaccination in three adult age groups: 18–55 (light blue symbols), 56–70 (dark blue), and over 70 years of age (black). [(A) to (C)] Anti-spike IgG, anti-RBD IgG, and PSV neutralizing titers. [(D) and (E)] Spike-specific CD4^+^ T cells by AIM (D) or ICS (E). (F) Spike-specific cT_FH_ cells. [(G) and (H)] Spike-specific CD8^+^ T cells by AIM (G) or ICS (H). Data were analyzed for statistical significance using nonparametric analysis of variance Kruskal-Wallis (KW) test and Dunn’s post-test for multiple comparisons. The *P* values plotted on the bottom show the KW test results, and the *P* values plotted on the top show the post-test analysis comparing age groups. (**I**) Anti-SARS-CoV-2 spike IgG, RBD IgG, and PSV neutralizing titers in 25-μg and 100-μg mRNA-1273 vaccinees at day 43 (2 weeks after second dose). (**J**) Spike-specific CD4^+^ AIM^+^ (left) and cytokine^+^ (right) T cells in mRNA-1273 vaccinees. (**K**) Spike-specific CD8^+^ AIM^+^ (left) and cytokine^+^ (right) T cells in mRNA-1273 vaccinees. Dotted green lines indicate the limit of quantification (LOQ). The bars indicate geometric mean and geomean SD. Data in (I) to (K) were analyzed for statistical significance using Mann-Whitney *U* test. Background-subtracted and log data analyzed in all cases.

Anti-spike antibody and CD4^+^ and CD8^+^ T cell responses generated by 25-μg mRNA-1273 vaccination were multifunctional, durable, and comparable in magnitude to those induced by natural infection (Table 1) (*22*). A concern has been raised that vaccination may not induce adequate immune memory in the elderly (*42*). Our vaccination cohort consisted of volunteers from three different age groups (*9*, *28*). Spike IgG and RBD IgG in the older groups (56–70 and >70 years) were reduced about twofold on day 209 (Fig. 4, A and B), similar to what was reported for 100-μg mRNA-1273 vaccination (*40*). Spike-specific CD4^+^ or CD8^+^ T cells were not reduced in the older vaccinee groups compared with the 18- to 55-year-old age group. Memory CD4^+^ and CD8^+^ T cell frequencies at day 209 were at least as strong in the older age groups as in younger adults (Fig. 4, D and H). Thus, although the study size is underpowered for in-depth examination of the three age groups, a small reduction in antibody but not T cell memory was observed in older adults compared with younger adults.

**Table 1.** Spike-specific immune responses detected in 25-μg mRNA-1273 vaccinees. ELISA, enzyme-linked immunosorbent assay; AIM, activation-induced markers; ICS, intracellular cytokine staining.

**Component**	**Assay**	**Days after vaccination**			
		1	15 ± 2	43 ± 2	209 ± 7
*Antibodies*					
Anti-spike IgG	ELISA	0	86%	100%	100%
Anti-RBD IgG	ELISA	3%	94%	100%	100%
Neutralizing	Neutralization	0	29%	100%	88%
*T cells*					
Spike-specific CD4^+^ T cells	AIM^*^	49%	97%	100%	97%
	ICS^†^	34%	94%	100%	97%
	Total^‡^	49%	97%	100%	97%
Spike-specific CD8^+^ T cells	AIM^*^	0	34%	53%	34%
	ICS^†^	0	51%	70%	54%
	Total^‡^	0	69%	88%	67%

*Antigen-specific T cells using AIM were measured as a percentage CD4^+^ T expressing OX40^+^CD137^+^ and CD8^+^ T cells expressing CD69^+^CD137^+^ after stimulation of PBMC with spike overlapping peptides spanning the entire protein.

†Antigen-specific T cells using ICS were measured as a percentage CD4^+^ T expressing CD40L or producing IFNγ, TNFα, IL-2, or GzB; and CD8^+^ T cells producing IFNγ, TNFα, IL-2, or GzB after stimulation of PBMC with spike overlapping peptides spanning the entire protein.

‡The overall spike-specific T cell response was calculated on the basis of the AIM and ICS results per donor and time point.

## Spike-specific immune responses elicited by the 100-μg mRNA-1273 vaccine dose

More than 100 million doses of the 100-μg mRNA-1273 vaccine have been administered in the United States to date. We compared immune responses to the 25-μg and 100-μg doses of mRNA-1273 (Fig. 4, I to K). Anti-spike IgG, anti-RBD IgG, and PSV neutralizing titers were about twofold higher in 100-μg vaccinees than in those who received the 25-μg dose (Fig. 4I), which is consistent with earlier reports (*9*, *28*, *40*). Spike-specific CD4^+^ T cells responses were ~1.4- to 2.0-fold higher in 100-μg vaccinees than in 25-μg vaccinees (Fig. 4J). Furthermore, the spike-specific CD8^+^ T cell responses were comparable between the doses (Fig. 4K).

## Preexisting cross-reactive memory

**Fig. 5.**
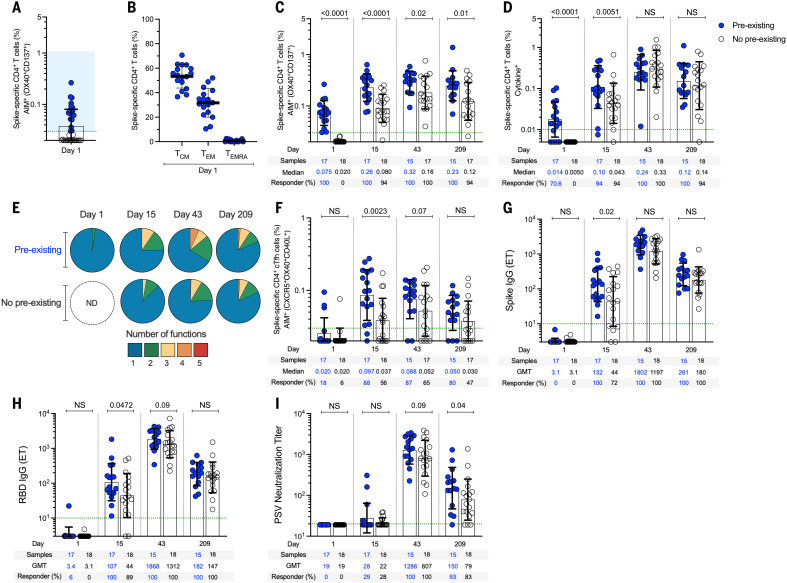
Preexisting anti-spike immunity modulates T cell and antibody responses. (**A**) Preexisting spike-specific CD4^+^ AIM^+^ T cells at day 1 (see Fig. 2 for details). (**B**) Memory phenotype of preexisting spike-specific CD4^+^ AIM^+^ T cells from (A). T_CM_, central memory cells; T_EM_, effector memory cells; T_EMRA_, terminally differentiated effector memory cells. (**C**) Spike-specific CD4^+^ AIM^+^ T cells in mRNA-1273 vaccinees with (“preexisting,” blue) and without (“no preexisting,” white) preexisting cross-reactive spike-reactive memory CD4^+^ T cells evaluated on days 1, 15 ± 2, 43 ± 2, and 209 ± 7 after immunization (see fig. S13 for details). (**D**) Spike-specific CD4^+^ cytokine^+^ T cells in mRNA-1273 vaccinees with and without preexisting cross-reactive memory CD4^+^ T cells. (**E**) Proportions of multifunctional spike-specific CD4^+^ T cells in mRNA-1273 vaccinees with (“pre-existing”) and without (“no preexisting”) preexisting cross-reactive spike-reactive memory CD4^+^ T cells evaluated on days 1, 15 ± 2, 43 ± 2, and 209 ± 7 after immunization (see fig. S10 for details). (**F**) Spike-specific cT_FH_ cells (as percentage of CD4^+^ T cells), (**G**) anti-spike IgG, (**H**) anti-RBD IgG, and (**I**) SARS-CoV-2 PSV neutralizing titers in mRNA-1273 vaccinees without and with preexisting cross-reactive spike-reactive memory CD4^+^ T cells. Dotted green lines indicate the LOQ. The bars in (A) and (C) to (I) indicate the geometric mean and geomean SD in the analysis of the antibody levels or spike-specific CD4^+^ and CD8^+^ T cells in mRNA-1273 vaccinees with (“preexisting”) and without (“no preexisting”) pre-existing cross-reactive spike-reactive memory CD4^+^ T cells evaluated on days 1, 15 ± 2, 43 ± 2, and 209 ± 7 after immunization. The bars in (B) indicate the mean and SD in the analysis of the memory phenotype of spike-specific CD4^+^ T cells. Data were analyzed for statistical significance using Mann-Whitney *U* test. Background-subtracted and log data analyzed in all cases.

Preexisting cross-reactive memory T cells recognizing SARS-CoV-2 in vitro are found in many individuals (*30*–*37*). It was hypothesized that the existence of preexisting spike-recognizing immune memory may modulate immune responses to infection or vaccination (*43*). To address this question, we separated our cohort as a function of whether each subject had preexisting cross-reactive memory CD4^+^ T cells reactive against SARS-CoV-2 spike (Fig. 5, A and B). As noted above (Fig. 2A), preexisting SARS-CoV-2 spike–specific CD4^+^ T cells were present in 49% (17/35) of the 25-μg mRNA-1273 vaccinees. After vaccination, spike-specific CD4^+^ T cells were significantly higher at day 15 in subjects with cross-reactive memory than in subjects with no cross-reactive memory (2.3-fold, *P* < 0.0001) (Fig. 5C). Spike-specific memory CD4^+^ T cell frequencies were also higher after the second vaccination in subjects with cross-reactive memory than in those without (*P* = 0.02) (Fig. 5) and remained higher for ≥6 months (*P* = 0.01) (Fig. 5C). The impact of preexisting cross-reactive spike-specific CD4^+^ T cell memory was more than additive (day 15, *P* = 0.018) (fig. S13), demonstrating that the spike-specific CD4^+^ T cell response to the vaccine was enhanced by cross-reactive memory. Higher frequencies of cytokine-positive spike-specific CD4^+^ T cells (*P* = 0.0051) (Fig. 5D) and multifunctional cells (*P* = 0.02) (Fig. 5E and fig. S10) were also observed after the first vaccination in individuals with cross-reactive memory. Preexisting cross-reactive CD4^+^ T cells were observed in all three age groups (Fig. 4D). We did not detect preexisting cross-reactive spike-specific CD8^+^ T cell memory (Figs. 3, A and D, and 4, G and H), and we observed no modulation of vaccine CD8^+^ T cell responses by preexisting CD4^+^ T cell memory (fig. S11). Cross-reactive memory CD4^+^ T cells recognizing SARS-CoV-2 nonspike epitopes were present (fig. S12), as expected (*31*, *34*). The nonspike cross-reactive memory CD4^+^ T cell frequencies remained unchanged over 7 months and were not modulated by mRNA-1273 vaccination (fig. S12), consistent with the vaccine containing only spike antigen and not causing bystander activation. Thus, preexisting cross-reactive CD4^+^ T cell memory can influence mRNA-1723 vaccine–generated CD4^+^ T cell responses.

T_FH_ cells in subjects with and without cross-reactive memory were of particular interest, because of their relevance in antibody responses. Frequencies of spike-specific cT_FH_ cells 2.6-fold higher were observed on day 15 in vaccinees with preexisting cross-reactive memory (*P* = 0.0023) (Fig. 5F). Likewise, significantly higher levels of anti-spike IgG (*P* = 0.02) (Fig. 5G) and anti-RBD IgG (*P* = 0.047) (Fig. 5H) were detected on day 15 in vaccinees with preexisting cross-reactive memory. The group with preexisting cross-reactive CD4^+^ T cell memory demonstrated higher SARS-CoV-2 neutralizing titers 7 months after vaccination than did the group without preexisting cross-reactive CD4^+^ T cell memory (*P* = 0.04) (Fig. 5I). Thus, a coordinated increase in spike-specific cT_FH_ responses, anti-spike IgG, and anti-RBD IgG are detected after a single vaccination in subjects with preexisting cross-reactive memory. Furthermore, 6 months after the second vaccination, higher levels of vaccine-specific memory CD4^+^ T cells and higher titers of SARS-CoV-2 PSV neutralizing antibodies are present in individuals who have preexisting cross-reactive CD4^+^ T cell memory.

## Concluding remarks

The SARS-CoV-2 mRNA vaccines have been extraordinary successes. It is important to better understand the immunology of these vaccines in order to better appreciate (i) the mechanisms of protective immunity provided by the vaccines, (ii) the durability of immune memory generated by the vaccines (and thus infer trajectories of protective immunity), and (iii) the immunological features of these vaccines that may be relevant for vaccine design against other pathogens. Here, studying 35 vaccinated subjects 7 months out from the initial immunization, we found that two-dose 25-μg mRNA-1273 vaccination generated immune memory against spike comparable to that of SARS-CoV-2 infection for antibodies, CD4^+^ T cells, and CD8^+^ T cells. Furthermore, immune responses were considerably enhanced by the presence of preexisting cross-reactive CD4^+^ T cell memory.

We consistently found spike-specific memory CD4^+^ T cells in vaccinated subjects 6 months after second-dose 25-μg mRNA-1273 immunization. Less than a twofold difference in spike-specific CD4^+^ T cell frequencies was observed between peak and 6 months after the second vaccination, indicative of durable vaccine T cell memory. Spike-specific memory CD4^+^ T cell frequencies were also similar between low-dose mRNA-1273 vaccinated persons and COVID-19 cases. The vaccine-generated cells also exhibited an antiviral functional profile, including substantial T_FH_ cell counts and IFNγ expression, and the presence of multi-cytokine-expressing cells in proportions similar to CMV-specific memory CD4^+^ T cells.

Uncertainty has surrounded whether the mRNA-1273 vaccine elicits effector and memory CD8^+^ T cells in humans (*9*, *44*, *45*). Here we report, at the peak of the immune response, spike-specific CD8^+^ T cells by AIM or ICS assays were detected in 88% of subjects receiving low-dose mRNA-1273, which is a CD8^+^ T cell response rate equivalent to that of COVID-19 cases (*21*, *22*, *26*, *31*). We speculate that absence of detection of spike-specific CD8^+^ T cells in some studies reflects the stringency of the experimental conditions used. Here, allowance for 24 hours of antigen stimulation revealed vaccine-generated CD8^+^ T cells in most individuals. CD8^+^ T cells have been observed in response to the Pfizer-BioNTech BNT162b2 vaccine using peptide–major histocompatibility complex multimers (*44*). Moreover, CD8^+^ T cells have been found 2 months after second immunization with BNT162b2 (*44*). In this study, spike-specific memory CD8^+^ T cells were detected 6 months after the second immunization with 25-μg mRNA-1273. These mRNA-1273 vaccine–generated memory CD8^+^ T cells were detected in 67% of subjects and were not dissimilar in magnitude to spike-specific memory CD8^+^ T cells in COVID-19 cases. Limitations of this study include the relatively small sample size and limited cell availability. Overall, the data show that CD4^+^ and CD8^+^ T cell memory are generated by both low-dose and 100-μg dose COVID-19 mRNA-1273 vaccine.

Low-dose RNA vaccines have potential advantages for future needs and applications such as dose sparing. Low-dose immunization is also less reactogenic (*9*, *44*), which may also be appealing in contexts of multidose regimens. It is of interest to consider different vaccine doses across age groups, or high- versus low-risk groups, but a better understanding of immune memory to different doses is key for such considerations. Data reported here are encouraging demonstrations of the potential of RNA vaccines to generate durable T cell and antibody immune memory, including at lower vaccine doses.

Preexisting immunity in the form of cross-reactive memory CD4^+^ T cells affected immune responses to the mRNA COVID-19 vaccine in this cohort. This indicates that cross-reactive memory T_FH_ cells may both accelerate B cell priming and antibody responses to a new antigen and increase robustness of long-term humoral immunity, as evidenced by the higher neutralizing antibody titers. Both total spike-specific CD4^+^ T cells and spike-specific T_FH_ cells were enhanced after one immunization in persons with cross-reactive memory, suggesting that the spike cross-reactive memory CD4^+^ T cells are recalled upon vaccination and affect the CD4^+^ T cell repertoire. By contrast, CD8^+^ T cell responses were unchanged. Our findings of preexisting immunity enhancing spike-specific CD4^+^ T cell, T_FH_, and antibody responses after immunization with an RNA vaccine do not represent the exact scenario of SARS-CoV-2 infection. It therefore remains unresolved whether preexisting T cells have a biological function during human SARS-CoV-2 infection (*33*). Nevertheless, these data provide evidence that the cross-reactive CD4^+^ T cells are biologically relevant in the context of vaccination. Thus, it is plausible that the presence and magnitude of cross-reactive memory T cells could accelerate the speed and magnitude of CD4^+^ T cell and antibody responses to SARS-CoV-2 infection, as compared with persons who have undetectable levels of cross-reactive memory T cells. Moreover, early T cell responses have been linked to less-severe COVID-19 clinical outcomes (*46*, *47*). These findings show substantial immune responses and immune memory to a low-dose mRNA vaccine and indicate biological relevance of cross-reactive memory T cells.

## Materials and methods

### Human subjects

#### Samples from the phase 1 25-μg mRNA-1273 SARS-CoV-2 trial

A total of 140 peripheral blood samples were obtained from 35 participants who received the 25-μg dose mRNA-1273 SARS-CoV-2 vaccine in a dose-escalation, open-label phase 1 trial (mRN-1273 ClinicalTrials.gov number NCT04283461) (*9*). Participants received two injections of the trial vaccine 28 days apart between March and April of 2020 at one of the two study sites, Kaiser Permanente Washington Health Research Institute in Seattle or at the Emory University School of Medicine in Atlanta. Peripheral blood mononuclear cell (PBMC) samples used in this study were collected at the start of the trial (day 1), 14 days after each vaccination (day 15 ± 2 and 43 ± 2) and 7 months after the first dose (day 209 ± 7).

Participants were divided into three age groups: 18 to 55 (*n* = 15), 56 to 70 (*n* = 10), and >70 (*n* = 10) years of age (table S3). Two participants from the 18–55 group received only one dose of the 25-μg mRNA-1273 vaccine. The day 40 and day 209 samples (both collected after the second dose) were excluded from analysis for these two vaccinees. Both sexes were represented [20:15, male:female (M:F)], and the average age of the participants was 58 years. Participants identified as white (*n* = 34), Hispanic (*n* = 1), or mixed race (Asian/Hispanic, *n* = 1.) An overview of samples analyzed in this study is provided in table S3.

The trial was reviewed and approved by the Advarra institutional review board, as previously published (*9*). All experiments performed at the La Jolla Institute (LJI) were approved by the institutional review board (IRB) of the La Jolla Institute (IRB#: VD-214). Collection and processing of vaccinee PBMC samples was performed at one of the two study location sites, as previously described (*9*).

#### Samples from convalescent COVID-19 donors

To compare levels of immune memory responses induced by 25-μg mRNA-1273 SARS-CoV-2 vaccination with immune memory responses induced by natural infection with SARS-CoV-2, we collected blood from individuals that experienced natural infection with SARS-CoV-2. We matched the 7-month (209-day) postvaccination samples with samples from convalescent donors collected on average 181 days (range: 170 to 195 days) post–symptom onset (PSO). Assuming an average incubation period of 12 days until symptom onset, the time point after exposure or vaccination is comparable between the cohorts (*48*). To further match ethnicities between the cohorts, we selected 13 samples from Caucasian donors and one sample from a Hispanic donor. Convalescent donors were California residents who were either referred to the study by a health care provider or self-referred. In the overall cohort, both sexes were represented (10:4, M:F), and the average age of the donors was 35 years (±16.53). Details of this COVID-19 convalescent cohort are listed in table S4. All of the convalescent donors experienced mild illness, defined as patients with a SARS-CoV-2 positive test who have never been hospitalized (*46*). Seropositivity against SARS-CoV-2 was confirmed by enzyme-linked immunosorbent assay (ELISA), as describe below. At the time of enrollment, all COVID-19 convalescent donors provided informed consent to participate in the present and future studies.

#### 100-μg mRNA-1273 vaccinees

We also included a cohort of individuals vaccinated locally in San Diego, California, who received the emergency use authorization–approved 100-μg mRNA-1273 SARS-CoV-2 vaccine. Samples from 20 vaccinees collected 42 ± 6 days after first immunization (15 days after second immunization) were compared with samples from the 25-μg mRNA-1273 cohort on day 43 ± 2. To match ethnicities between the cohorts, we selected 12 samples from Caucasian donors, 4 samples from Asian donors, and 4 samples from Hispanic donors. In the overall cohort, both sexes were represented (6:14, M:F) and the average age of the donors was 48 years (±14.45). Details of this cohort are listed in table S5. At the time of enrollment, all 100-μg mRNA-1273 SARS-CoV-2 vaccinees provided informed consent to participate in the present and future studies.

#### Peripheral blood mononuclear cells (PBMCs) and plasma isolation

Whole blood samples from convalescent COVID-19 donors and 100-μg mRNA-1273 vaccinees were collected at La Jolla Institute in heparin-coated blood bags and centrifuged for 15 min at 803*g* to separate the cellular fraction and plasma. The plasma was then carefully removed from the cell pellet and stored at −20°C. PBMCs were isolated by density-gradient sedimentation using Ficoll-Paque (Lymphoprep, Nycomed Pharma, Oslo, Norway), as previously described (*22*, *31*). Isolated PBMCs were cryopreserved in cell recovery media containing 10% dimethyl sulfoxide (DMSO) (Gibco), supplemented with 10% heat-inactivated fetal bovine serum (FBS; Hyclone Laboratories, Logan UT), and stored in liquid nitrogen until used in the assays.

### Antibody measurements

#### SARS-CoV-2 ELISAs

SARS-CoV-2 ELISA titers in vaccinated and convalescent samples were determined as previously described (*22*, *31*, *46*, *49*). Endpoint titers (ETs) were plotted for each sample, using background subtracted data, with ETs calculated at the dilution giving a reading above the optical density cutoff of 0.1. Negative and positive controls were used to standardize each assay and normalize across experiments. A positive control standard was created by pooling plasma from six convalescent COVID-19 donors to normalize between experiments. The limit of detection was defined as 1:3 for IgG. The limit of quantification (LOQ) for vaccinated individuals was established on the basis of uninfected subjects, using plasma from healthy donors never exposed to or vaccinated against SARS-CoV-2.

#### Pseudovirus (PSV) neutralization assay

The PSV neutralization assays of vaccinated and convalescent samples were performed as previously described (*22*). Neutralization titers or inhibition dose 50 (ID_50_) were calculated using the One-Site Fit Log IC50 model in Prism 9.2 (GraphPad). As internal quality control to define the interassay variation, three samples were included across the PSV neutralization assays. Samples that did not reach 50% inhibition at the lowest serum dilution of 1:20 were considered to be non-neutralizing, and the values were set to 19. PSV neutralization titers were performed as two independent experiments on different days with two replicates per experiment. Results were comparable between experiments and results from the first experiment are graphed. We included the World Health Organization (WHO) International Reference Panel for anti-SARS-Cov2 immunoglobulin (20/268) to calibrate our PSV neutralization titers. The ID_50_ of WHO-High and WHO-Mid were measured by four independent experiments with two replicates per experiment. The geometric means (GMTs) of ID_50_ of WHO-High and WHO-Mid were 2658 and 364, respectively, by our PSV neutralization assay. The WHO assigned neutralization activity unitage of 1473 and 210 IU/ml for the WHO-High and WHO-Mid standards. The calibration factor was thus calculated as ((2658/1473) + (364/ 210))/ 2 = 1.766. The GMTs of PSV neutralization ID_50_ in 25-µg and 100-µg mRNA-1273 vaccinees at day 43 were 997 and 2000, respectively, in our figures. The WHO IU calibrated neutralization ID_50_ (cID_50_) GMTs in 25-µg mRNA-1273 vaccinees at day 43 would be 565 IU (997/1.766), and for 100-µg mRNA-1273 vaccinees at day 43 would be 1133 IU (2000/1.766). The limit of detection was calculated as 10.7 IU (19/1.766).

### Peptide megapools (MPs)

We have previously developed the MP approach to allow simultaneous testing of a large number of epitopes (*22*, *31*, *46*, *50*). Here, three MPs to evaluate the antigen-specific T cell response against SARS-CoV-2 were used, as described below. A cytomegalovirus (CMV) MP was used as a control against a ubiquitous pathogen in the experiments.

#### SARS-CoV-2 MPs

To characterize SARS-CoV-2–specific T cell response, we used three MPs previously described (*31*, *43*). First, we used a spike MP of 253 overlapping peptides spanning the entire sequence of the spike protein. As this peptide pool consists of peptides with a length of 15 amino acids, both CD4^+^ and CD8^+^ T cells have the capacity to recognize this MP (*51*). In addition, to confirm that the spike-specific CD8^+^ T cell response observed in the mRNA-1273 vaccinees is also induced in the absence of the spike-specific CD4^+^ T cell response, we performed some experiments with an optimal MP of HLA class I epitopes (CD8-S MP). This CD8-S MP consists of 197 9- and 10-mers derived spike peptides that have previously been described to be recognized by CD8^+^ T cells in SARS-CoV-2 exposed donors (*22*, *31*, *46*, *52*). Lastly, we used a predicted SARS-CoV-2–specific MP (CD4-R) to evaluate the nonspike response or the remainder of the SARS-CoV-2 genome of 246 HLA class II CD4^+^ epitopes previously described (*31*). We have previously shown that these MPs are suitable for stimulating T cell responses from either COVID-19–exposed or SARS-CoV-2–unexposed individuals (*31*, *34*).

#### Cytomegalovirus (CMV) MP

As a control, we used a MP of 313 experimentally defined epitopes. This CMV MP consists of HLA class I and class II epitopes and CD4^+^ and CD8^+^ T cells have the capacity to recognize this MP, as has been previously published (*50*).

### Flow cytometry assays

#### Activation-induced markers (AIM) assay

Antigen-specific CD4^+^ T cells were measured as a percentage of AIM^+^ (OX40^+^CD137^+^) CD4^+^ and (CD69^+^CD137^+^) CD8^+^ T cells after stimulation of PBMCs from mRNA-1273 vaccinees and COVID-19 convalescent donors with peptide MPs. Antigen-specific circulating T follicular helper (cT_FH_) cells (CXCR5^+^OX40^+^CD40L^+^, as percentage of CD4^+^ T cells) were defined by the AIM assay.

Before addition of MPs, cells were blocked at 37°C for 15 min with 0.5 μg/ml of anti-CD40 monoclonal antibody (mAb) (Miltenyi Biotec). Then, cells were incubated at 37°C for 24 hours in the presence of fluorescently labeled anti-chemokine receptor antibodies (anti-CCR4, -CCR6, -CCR7, -CXCR3, and -CXCR5) and SARS-CoV-2 MPs (1 μg/ml) or CMV MP (1 μg/ml) in 96-well U-bottom plates, as previously described (*31*, *46*). In addition, PBMCs were incubated with an equimolar amount of DMSO as negative control and with phytohemagglutinin (5 μg/ml) (PHA, Roche) as a positive control.

For the surface stain, 1 × 10^6^ PBMCs were resuspended in phosphate-buffered saline (PBS), incubated with BD human FC block (BD Biosciences, San Diego, CA) and the LIVE/DEAD marker in the dark for 15 min and washed with PBS. Then, an antibody mix containing the rest of the surface antibodies was added directly to cells and incubated for 60 min at 4°C in the dark. After surface staining, cells were washed twice with PBS containing 3% FBS (FACS buffer). All samples were acquired on a Cytek Aurora (Cytek Biosciences, Fremont, CA). A list of antibodies used in this panel can be found in table S6, and a representative gating strategy of spike-specific CD4^+^ and CD8^+^ T cells using the AIM assay is shown in fig. S2.

Antigen-specific CD4^+^ and CD8^+^ T cells were measured as background (DMSO)–subtracted data, with a minimal DMSO level set to 0.005%. A response of >0.02% and a stimulation index (SI) of >2 for CD4^+^, and a response of >0.03% and SI of >3 for CD8^+^ T cells, were considered positive. The LOQ for antigen-specific CD4^+^ T cell responses (0.03%) and antigen-specific CD8^+^ T cell responses (0.06%) was calculated using the median twofold standard deviation of all negative controls. As an internal quality control to define interassay variation, the CMV-specific CD4^+^ and CD8^+^ T cell responses were evaluated for a SARS-CoV-2–unexposed donor included in each independent experiment. The antigen-specific response against CMV and the response to the positive control was compared across experiments and revealed a coefficient of variation (CV) between 10 and 13% for the antigen-specific stimulation with the CMV-MP and a CV between 6 and 12% for mitogenic stimulation with PHA (fig. S2).

#### Intracellular cytokine staining (ICS) assay

To optimize spike-specific detection of cytokine-producing CD4^+^ and CD8^+^ T cells, we experimented with different incubation times (5, 6, and 24 hours) in the presence of GolgiPlug containing brefeldin A and GolgiStop containing monensin (BD Biosciences, San Diego, CA) for an additional 1, 3, or 4 hours, respectively. To establish optimal conditions for the ICS assay, we evaluated the IFNγ-producing CD4^+^ and CD8^+^ T cells in 100-μg mRNA-1273 vaccinees (*n* = 4) and COVID-19 convalescent donors (*n* = 4) (table S7). The highest signal of IFNγ-producing CD4^+^ and CD8^+^ T cells was detected after 24+4 hours incubation in both vaccinees and convalescent donors. Thus, we chose 24 hours as the best condition to identify spike-specific CD4^+^ and CD8^+^ T cells producing intracellular cytokines.

Before the addition of MPs, cells were blocked at 37°C for 15 min with 0.5 μg/ml of anti-CD40 mAb, as previously described (*46*). PBMCs were cultured in the presence of SARS-CoV-2 MPs (1 μg/ml) for 24 hours at 37°C. In addition, PBMCs were incubated with an equimolar amount of DMSO as a negative control and also CMV MP (1 μg/ml) as a positive control. After 24 hours, Golgi-Plug and Golgi-Stop were added to the culture for 4 hours, as described above. Cells were then washed and surface-stained for 30 min at 4°C in the dark and fixed with 1% of paraformaldehyde (Sigma-Aldrich, St. Louis, MO). Antibodies used in the ICS assay are listed in table S8, and a representative gating strategy of spike-specific CD4^+^ and CD8^+^ T cells using the ICS assay is shown in fig. S4.

Antigen-specific CD4^+^ and CD8^+^ T cells were measured as background (DMSO) subtracted data, with a minimal DMSO level set to 0.001%. Responses of >0.005% and a SI of >2 for CD4^+^ and CD8^+^ T cells were considered positive. The limit of quantification for antigen-specific CD4^+^ and CD8^+^ T cell responses (0.01%) was calculated using the median twofold standard deviation of all negative controls. As internal quality control to define interassay variation, the CMV-specific CD4^+^ and CD8^+^ T cell responses were evaluated for a SARS-CoV-2–unexposed donor included in each independent experiment. The antigen-specific response of CD4^+^ and CD8^+^ T cells against CMV were compared across experiments and revealed a CV of 14% for the antigen-specific stimulation with the CMV MP (fig. S4E).

The gates applied for the identification of CD4^+^ and CD8^+^ T cells producing cytokines were defined according to the cells cultured with DMSO for each individual as is shown in fig. S4. A Boolean analysis was performed to define the multifunctional profiles on FlowJo 10.7.1. The analysis included CD40L, GzB, IFNγ, IL-2, and TNFα gated on CD3^+^CD4^+^ cells and GzB, IFNγ, IL-2, and TNFα gated on CD3^+^CD8^+^ cells. The overall response to spike was defined as the sum of the background subtracted responses to each combination of individual cytokines. To define the multifunctional profiles of antigen-specific T cells, all positive background-subtracted data (>0.005% and a SI > 2 for CD4^+^ T cells and >0.002% and a SI > 2 for CD8^+^ T cells) was aggregated into a combined sum of antigen-specific CD4^+^ or CD8^+^ T cells on the basis of the number of functions. Values higher than the LOQ (0.01%) were considered for the analysis of the multifunctional antigen-specific T cell responses. The average of the relative CD4^+^ and CD8^+^ T cell response was calculated per group to define the proportion of multifunctional antigen-specific T cell responses (figs. S6 and S7 and tables S1 and S2).

### Statistical analysis

Data were analyzed using FlowJo 10.7.1. Statistical analyses were performed in GraphPad Prism 9.2, unless otherwise stated. The statistical details of the experiments are provided in the respective figure legends. Data plotted in linear scale were expressed as means ± standard deviations (SD). Data plotted in logarithmic scales were expressed as geometric means ± geometric standard deviations (SD). Mann-Whitney *U* or Wilcoxon tests were applied for unpaired or paired comparisons, respectively. Differences among age groups were evaluated using Kruskal-Wallis and Dunn’s post-test for multiple comparisons. Details pertaining to significance are also noted in the respective legends.

## Supplementary Material

20210914-1

## Supplementary Materials

### This PDF file includes:

Figs. S1 to S13 Tables S1 to S8 References

### Other Supplementary Material for this manuscript includes the following:

MDAR Reproducibility Checklist

## Notes

View/request a protocol for this paper from *Bio-protocol*.
