# Supplementary material for: Low-dose mRNA-1273 COVID-19 vaccine generates durable memory enhanced by cross-reactive T cells
Source: Science. 2021 Oct 22;374(6566):eabj9853. doi: 10.1126/science.abj9853 (PMC8542617; doi:10.1126/science.abj9853)
Supplement: 20210914-1 [file science.abj9853.v1.pdf]

Science

RESEARCH ARTICLES

Cite as: J. Mateus et al., Science 10.1126/science.abj9853 (2021).

Low-dose mRNA-1273 COVID-19 vaccine generates durable memory enhanced by cross-reactive T cells

Jose Mateus[1], Jennifer M. Dan[1,2]†, Zeli Zhang[1]†, Carolyn Rydyznski Moderbacher[1], Marshall Lammers[1], Benjamin Goodwin[1], Alessandro Sette[1,2]*, Shane Crotty[1,2]*, Daniela Weiskopf[1]*

[1]Center for Infectious Disease and Vaccine Research, La Jolla Institute for Immunology (LJI), La Jolla, CA 92037, USA. [2]Department of Medicine, Division of Infectious Diseases and Global Public Health, University of California, San Diego (UCSD), La Jolla, CA 92037, USA.

†These authors contributed equally to this work.

***Corresponding author. Email: alex@lji.org (A.S.); shane@lji.org (S.C.); daniela@lji.org (D.W.)**

Vaccine-specific CD4$^+$ T cell, CD8$^+$ T cell, binding antibody, and neutralizing antibody responses to the 25-μg Moderna mRNA-1273 vaccine were examined over 7 months post-immunization, including multiple age groups, with a particular interest in assessing whether pre-existing cross-reactive T cell memory impacts vaccine-generated immunity. Vaccine-generated spike-specific memory CD4$^+$ T cells 6 months post-boost were comparable in quantity and quality to COVID-19 cases, including the presence of T follicular helper cells and IFNγ-expressing cells. Spike-specific CD8$^+$ T cells were generated in 88% of subjects, with equivalent memory at 6 months post-boost compared to COVID-19 cases. Lastly, subjects with pre-existing cross-reactive CD4$^+$ T cell memory had increased CD4$^+$ T cell and antibody responses to the vaccine, demonstrating the biological relevance of SARS-CoV-2–cross-reactive CD4$^+$ T cells.

Understanding human immune responses to SARS-CoV-2 RNA vaccines is of interest for a panoply of reasons. mRNA vaccines have demonstrated impressive protection from COVID-19 (*1*–*7*). The COVID-19 vaccine mRNA-1273 encodes a stabilized SARS-CoV-2 full-length spike, developed as a collaboration between Moderna and the NIH Vaccine Research Center (*8*, *9*). Durability of immunity has been, and remains, a major unknown for mRNA vaccines in humans. Encouraging reports from both Pfizer/BioNTech and Moderna indicate protective immunity of 91% and 93% over the 6-month period after the second immunization (7 months after first immunization) (*10*, *11*), down modestly from the 95% maximal protection observed for each of those two vaccines within 1-2 months after two immunizations (*1*, *2*). Although neutralizing antibodies are a clear correlate of immunity after two immunizations (*12*), the underlying mechanisms remain unclear. Moreover, those mechanisms of immunity may change as the immune response develops (e.g., after a single immunization) or as immune memory changes composition (*13*–*15*). Direct measurements of immune memory compartments in humans are necessary to provide insights into these important topics.

Infection and vaccination are two different paths to immunity. Comparison of vaccine-generated immune memory to immune memory of persons infected with SARS-CoV-2 is of value, as studies have indicated natural immunity is 93% to 100% protective against symptomatic reinfection for 7-8 months (*16*–*19*), though natural immunity protection against certain variants of concern (VOCs) is likely to be lower (*20*). After SARS-CoV-2 infection, immunological memory has been observed for ≥8 months for CD4$^+$ T cells, CD8$^+$ T cells, memory B cells, and antibodies (*21*, *22*). The immune memory in response to SARS-CoV-2 infection exhibits a relatively gradual decline that partially stabilizes within 1 year (*23*–*26*). The 100-μg mRNA-1273 vaccination has been shown to induce durable antibody responses (*27*) but it is unknown whether immune memory to the mRNA-1273 vaccine months after immunization is similar or different than memory generated by SARS-CoV-2 infection. Additionally, both 25-μg- and 100-μg-dose mRNA-1273 vaccinations have been tested in clinical trials (*9*, *28*), with 100-μg mRNA-1273 proceeding toward licensure (*2*, *29*).

Pre-existing cross-reactive memory CD4$^+$ T cells that recognize SARS-CoV-2 have been found in ~50% of individuals pre-pandemic (*30*–*37*). There has been intense interest in understanding whether these pre-existing cross-reactive memory CD4$^+$ T cells, identified in vitro, are biologically relevant in vivo (*33*, *38*, *39*). One approach to test the relevance of such T cells in a controlled fashion is in the context of a vaccine trial, as individuals in a clinical trial are all exposed to a well-defined dose of antigen at a specific time. Additionally, exposure to a low antigen dose may be more sensitive to influence by cross-reactive memory. Thus, we examined immune responses to the 25-μg dose of the mRNA-1273 COVID-19 vaccine.

Spike-specific antibody elicited by the 25-μg mRNA-1273 vaccine dose over time

An open-label, age de-escalation phase 1 trial utilized the mRNA-1273 vaccine with 25-μg immunizations on days 1 and 29 (*9*, *28*), with blood samples collected on study day 1, 15, 43, and 209. SARS-CoV-2 spike–binding antibodies, receptor-binding domain (RBD)–binding antibodies, and SARS-CoV-2 pseudovirus (PSV) neutralization titers were determined (Fig. 1). Anti-spike and -RBD IgG were maintained at detectable levels for at least 7 months after the first vaccination, for 100% (33/33) of subjects (Fig. 1, A and B). RBD IgG was induced by one immunization in 94% (33/35) of subjects. This response rate increased to 100% (33/33) after the second immunization and was maintained for at least 6 months after the second vaccination. SARS-CoV-2 PSV neutralizing titers were detected in 29% (10/35) of subjects after one vaccination, 100% after two vaccinations (33/33), and 88% (29/33) maintained detectable neutralizing antibodies for at least 6 months after the second vaccination (Fig. 1C). All three antibody measurements demonstrated similar kinetics (Fig. 1, A to C) and were highly correlated (r = 0.89-0.90, fig. S1). Anti-spike IgG, anti-RBD IgG, and PSV titers at 7 months (study day 209; 181 days after the second immunization) were 6.8-fold, 9.5-fold, and 9.5-fold lower than peak titers, respectively. Similar fold changes were reported for 100-μg mRNA-1273 vaccination, indicating similar memory quality and durability (*40*).The 25-μg mRNA-1273 vaccine-generated antibodies were comparable to antibodies from SARS-CoV-2-infected subjects collected at a similar time post-exposure (7 months post-symptom onset (PSO), 170-195 days) (Fig. 1D). Thus, significant anti-spike IgG, anti-RBD IgG, and PSV-neutralizing antibodies were induced in response to two 25-μg mRNA-1273 vaccinations. These levels were maintained in 88% to 100% of vaccinees for at least 6 months after the second immunization and were comparable to those observed after infection with SARS-CoV-2.

Spike-specific CD4$^+$ T cells elicited by the 25-μg mRNA-1273 vaccine dose over time

SARS-CoV-2 spike-specific CD4$^+$ T cell responses were first measured utilizing a flow cytometry activation-induced marker (AIM) assay (Fig. 2A and fig. S2). On day 1, before vaccination, spike-specific CD4$^+$ T cells with a predominantly memory phenotype were detected in 49% of clinical trial subjects (17/35), demonstrating the presence of pre-existing SARS-CoV-2 spike–cross-reactive memory CD4$^+$ T cells, as discussed in the latter part of this report. Spike-specific CD4$^+$ T cell responses were observed after the first vaccination in 97% of subjects (34/35) (Fig. 2A). CMV-specific CD4$^+$ T cells were unchanged, as expected, indicating no bystander influence of the mRNA-1273 vaccination (fig. S3). The SARS-CoV-2 spike–specific CD4$^+$ T cell response rate increased to 100% (32/32) after the second vaccination and was maintained for at least 6 more months. Spike-specific memory CD4$^+$ T cell frequencies at 7 months were similar to those observed for COVID-19 cases (COVID-19 samples collected 170-195 days PSO) (Fig. 2B). Median mRNA-1273 vaccine-generated spike-specific CD4$^+$ T cell frequencies at all time points post-vaccination also exceeded CMV-specific CD4$^+$ T cell frequencies (Fig. 2A and fig. S3A).

T follicular helper (T_{FH}) cell differentiation and cytokine production by vaccine-generated spike-specific CD4$^+$ T cells were then assessed (Fig. 2, C to F). T_{FH} cells are the specialized subset of CD4$^+$ T cells required for B cell help and are critical for the generation of neutralizing antibodies in most conditions (*41*). Spike-specific circulating T_{FH} (cT_{FH}) cells were detected in 71% (25/35) and 75% (24/32) of subjects after the first and second vaccination, respectively (Fig. 2C, right panel). Spike-specific cT_{FH} cells were detectable in 94% of subjects overall (32/35). Different response kinetics were observed at the level of individual subjects (Fig. 2C, right panel). Spike-specific memory cT_{FH} cells were still detected in 63% of vaccinees 6 months after the second vaccination (20/32) (Fig. 2C). Vaccine-specific CD4$^+$ T cell cytokine profiles were determined by intracellular cytokine staining (ICS) (Fig. 2, D to F, and fig. S4). IFNγ$^+$ spike-specific CD4$^+$ T cells were detected in 89% (28/33), TNFα$^+$ in 97% (32/33), IL-2$^+$ in 100% (33/33), and GzB$^+$ in 76% (25/33) of subjects at day 43 (Fig. 2D). Little-to-no IL-4, IL-17A, or IL-10 was detected (fig. S5). Cytokine-producing spike-specific CD4$^+$ T cells (CD40L$^+$ cells producing IFNγ, TNFα, IL-2, and/or GzB) (Fig. 2E and fig. S6) were observed in 94% (33/35) and 100% (33/33) of subjects after the first and second vaccination, respectively, and were maintained for at least 6 months after the second vaccination (97%, 32/33 of the subjects) (Fig. 2E). Spike-specific CD4$^+$ T cells generated by the 25-μg mRNA-1273 vaccine exhibited multifunctionality comparable to that of CMV-specific cells (Fig. 2F, fig. S3C, and table S1). Thus, robust spike-specific CD4$^+$ T cells and T cell memory were generated by low-dose mRNA-1723 vaccine, with strong T_{FH} and T_H1 cell polarization advantageous for antiviral immunity.

Spike-specific CD8$^+$ T cells elicited by the 25-μg mRNA-1273 vaccine dose over time

SARS-CoV-2 spike-specific CD8$^+$ T cells were measured by AIM (CD69$^+$ and CD137$^+$, fig. S2) and were observed in 34% (12/35) and 53% (17/32) of subjects after the first and second 25-μg mRNA-1273 vaccination, respectively (Fig. 3A). Spike-specific CD8$^+$ T cells were detectable for >6 months after the second vaccination, with a response rate comparable to that observed for COVID-19 cases (COVID-19 samples collected 170-195 days PSO) (Fig. 3B). Next, SARS-CoV-2 spike-specific CD8$^+$ T cells were measured by ICS (IFNγ, TNFα, IL-2 or GzB) (Fig. 3C and fig. S4). The first immunization induced

significant spike-specific CD8+ T cell cytokine responses in 51% (18/35) of subjects (Fig. 3D and fig. S7), increasing to 70% (23/33) of subjects after the second vaccination (Fig. 3D). IFNγ+ spike-specific CD8+ T cells were detected in 70% (23/33), TNFα in 39% (13/33), and IL-2+ in 12% (4/33) of vaccinees at day 43 (Fig. 3C). Multiple positive- and negative-control samples and experimental conditions were used to demonstrate the specificity of the spike CD8+ T cells (Fig. 3 and figs. S2 and S4, F and G). Correlation between AIM and ICS methods was highly significant for both spike-specific CD4+ and CD8+ T cells ($P < 0.0001$) (fig. S8). The fraction of multifunctional spike-specific CD8+ T cells increased between first and second vaccination (three or more effector molecules expressed) (Fig. 3E and table S2). The most prevalent profile of CD8+ T cells with three functions was GzB+IFNγ+TNFα+ (fig. S7), similar to the profile seen in CMV-specific CD8+ T cells (Fig. 3D and fig. S9). Thus, 25-μg mRNA-1273 vaccination induces multifunctional spike-specific memory CD8+ T cells.

Anti-spike antibody and CD4+ and CD8+ T cell responses generated by 25-μg mRNA-1273 vaccination were multifunctional, durable, and comparable in magnitude to those induced by natural infection (Table 1) (*22*). A concern has been raised that vaccination may not induce adequate immune memory in the elderly (*42*). This vaccination cohort consisted of volunteers from three different age groups (*9*, *28*). Spike IgG and RBD IgG in the older groups (56-70 and >70 years) were reduced approximately twofold on day 209 (Fig. 4, A and B), similar to that reported for 100-μg mRNA-1273 vaccination (*40*). Spike-specific CD4+ or CD8+ T cells were not reduced in the older vaccinee groups compared to the 18–55-year-old age group. Memory CD4+ and CD8+ T cell frequencies at day 209 were at least as strong in the older age groups as the younger adults (Fig. 4, D and H). Thus, although the study size is underpowered for in-depth examination of the three age groups, a small reduction in antibody but not T cell memory was observed in older adults compared to younger adults.

Spike-specific immune responses elicited by the 100-μg mRNA-1273 vaccine dose

Over 100 million doses of the 100-μg mRNA-1273 vaccine have been administered in the USA to date. We compared immune responses between the 25-μg and 100-μg doses of mRNA-1273 (Fig. 4, I to K). Anti-spike IgG, anti-RBD IgG, and PSV-neutralizing titers were approximately twofold higher in 100-μg vaccinees compared to those who received the 25-μg dose (Fig. 4I), consistent with earlier reports (*9*, *28*, *40*). Spike-specific CD4+ T cells responses were ~1.4- to 2.0-fold higher in 100-μg vaccinees compared to 25-μg vaccinees (Fig. 4J). Furthermore, the spike-specific CD8+ T cell responses were comparable between the doses (Fig. 4K).

Pre-existing cross-reactive memory

Pre-existing cross-reactive memory T cells recognizing SARS-CoV-2 in vitro are found in many individuals (*30*–*37*). It was hypothesized that the existence of pre-existing spike-recognizing immune memory may modulate immune responses to infection or vaccination (*43*). To address this question, we separated our cohort as a function of whether each subject possessed pre-existing cross-reactive memory CD4+ T cells reactive against SARS-CoV-2 spike (Fig. 5, A and B). As noted above (Fig. 2A), pre-existing SARS-CoV-2 spike-specific CD4+ T cells were present in 49% (17/35) of the 25-μg mRNA-1273 vaccinees. After vaccination, spike-specific CD4+ T cells were significantly higher at day 15 in subjects with cross-reactive memory compared to subjects with no cross-reactive memory (2.3-fold; $P < 0.0001$, Fig. 5C). Spike-specific memory CD4+ T cell frequencies were also higher after the second vaccination in subjects with cross-reactive memory compared to those without ($P = 0.02$, Fig. 5), and remained higher for ≥6 months ($P = 0.01$) (Fig. 5C). The impact of pre-existing cross-reactive spike-specific CD4+ T cell memory was more than additive (Day 15, $P = 0.018$) (fig. S13), demonstrating that the spike-specific CD4+ T cell response to the vaccine was enhanced by cross-reactive memory. Higher frequencies of cytokine-positive spike-specific CD4+ T cells ($P = 0.0051$) (Fig. 5D) and multifunctional cells ($P = 0.02$) (Fig. 5E and fig. S10) were also observed after the first vaccination in individuals with cross-reactive memory. Pre-existing cross-reactive CD4+ T cells were observed in all three age groups (Fig. 4D). We did not detect pre-existing cross-reactive spike-specific CD8+ T cell memory (Figs. 3, A and D, and 4, G and H), and observed no modulation of vaccine CD8+ T cell responses by pre-existing CD4+ T cell memory (fig. S11). Cross-reactive memory CD4+ T cells recognizing SARS-CoV-2 non-spike epitopes were present (fig. S12), as expected (*31*, *34*). The non-spike-cross-reactive memory CD4+ T cell frequencies remained unchanged over 7 months and were not modulated by mRNA-1273 vaccination (fig. S12), consistent with the vaccine containing only spike antigen and not causing bystander activation. Thus, pre-existing cross-reactive CD4+ T cell memory can influence mRNA-1723 vaccine-generated CD4+ T cell responses.

T_{FH} cells in subjects with and without cross-reactive memory were of particular interest, because of their relevance in antibody responses. Frequencies of spike-specific cT_{FH} cells 2.6-fold higher were observed on day 15 in vaccinees with pre-existing cross-reactive memory ($P = 0.0023$, Fig. 5F). Likewise, significantly higher levels of anti-spike IgG ($P = 0.02$) (Fig. 5G) and anti-RBD IgG ($P = 0.047$) (Fig. 5H) were detected on day 15 in vaccinees with pre-existing cross-reactive memory. The group with pre-existing cross-reactive CD4+ T cell memory demonstrated higher SARS-CoV-2 neutralizing titers 7 months post-vaccination compared to the group without pre-existing cross-reactive CD4+ T cell memory ($P = 0.04$,

Fig. 5I). Thus, a coordinated increase in spike-specific cT_{FH} responses, anti-spike IgG, and anti-RBD IgG are detected after a single vaccination in subjects with pre-existing cross-reactive memory. Furthermore, 6 months after the second vaccination, higher frequencies of vaccine-specific memory $CD4^+$ T cells and higher titers of SARS-CoV-2 neutralizing antibodies are present in individuals who have pre-existing cross-reactive $CD4^+$ T cell memory.

Concluding remarks

The SARS-CoV-2 mRNA vaccines have been extraordinary successes. It is important to better understand the immunology of these vaccines in order to better appreciate (i) the mechanisms of protective immunity provided by the vaccines, (ii) the durability of immune memory generated by the vaccines (and thus infer trajectories of protective immunity), and (iii) the immunological features of these vaccines that may be relevant for vaccine design against other pathogens. Here, studying 35 vaccinated subjects out to 7 months from the initial immunization, we found that two dose 25-μg mRNA-1273 vaccination generated immune memory against spike comparable to that of SARS-CoV-2 infection for antibodies, $CD4^+$ T cells, and $CD8^+$ T cells. Furthermore, immune responses were significantly enhanced by the presence of pre-existing cross-reactive $CD4^+$ T cell memory.

We consistently found spike-specific memory $CD4^+$ T cells in vaccinated subjects 6 months after second dose 25-μg mRNA-1273 immunization. Less than a twofold difference in spike-specific $CD4^+$ T cell frequencies was observed between peak and 6 months post-boost, indicative of durable vaccine T cell memory. Spike-specific memory $CD4^+$ T cell frequencies were also similar between low-dose mRNA-1273 vaccinated persons and COVID-19 cases. The vaccine-generated cells also exhibited an antiviral functional profile, including substantial T_{FH} cells and IFNγ expression, and the presence of multi-cytokine-expressing cells in proportions similar to CMV-specific memory $CD4^+$ T cells.

Uncertainty has surrounded whether the mRNA-1273 vaccine elicited effector and memory $CD8^+$ T cells in humans (*9*, *44*, *45*). Here we report, at the peak of the immune response, spike-specific $CD8^+$ T cells by AIM or ICS assays were detected in 88% of subjects receiving low-dose mRNA-1273, which is a $CD8^+$ T cell response rate equivalent to that of COVID-19 cases (*21*, *22*, *26*, *31*). We speculate that absence of detection of spike-specific $CD8^+$ T cells in some studies reflected the stringency of the experimental conditions used. Here, allowance for 24 hours of antigen stimulation revealed vaccine-generated $CD8^+$ T cells in most individuals. Using peptide–MHC multimers, $CD8^+$ T cells have been observed to the Pfizer/BioNTech BNT162b2 vaccine (*44*). Moreover, $CD8^+$ T cells are found 2 months after second immunization with BNT162b2 (*44*). Here, spike-specific memory $CD8^+$ T cells were detected 6 months after the second immunization with 25-μg mRNA-1273. These mRNA-1273 vaccine-generated memory $CD8^+$ T cells were detected in 67% of subjects and were not dissimilar in magnitude to spike-specific memory $CD8^+$ T cells in COVID-19 cases. Limitations of this study include the relatively small sample size and limited cell availability. Overall, the data show that $CD4^+$ and $CD8^+$ T cell memory are generated by both low-dose and 100-μg dose COVID-19 mRNA-1273 vaccine.

Low-dose RNA vaccines have potential advantages for future needs and applications such as dose sparing. Low-dose immunization is also less reactogenic (*9*, *44*), which may also be appealing in contexts of multi-dose regimens. It is of interest to consider different vaccine doses across age groups, or high- versus low-risk groups, but a better understanding of immune memory to different doses is key for such considerations. Data reported here are encouraging demonstrations of the potential of RNA vaccines to generate durable T cell and antibody immune memory, including at lower vaccine doses.

Pre-existing immunity in the form of cross-reactive memory $CD4^+$ T cells impacted immune responses to the RNA COVID-19 vaccine in this cohort. This indicates that cross-reactive memory T_{FH} cells may both accelerate B cell priming and antibody responses to a new antigen, and also increase robustness of long-term humoral immunity, as evidenced by the higher neutralizing antibody titers. Both total spike-specific $CD4^+$ T cells and spike-specific T_{FH} cells were enhanced after one immunization in persons with cross-reactive memory, suggesting that the spike cross-reactive memory $CD4^+$ T cells are recalled upon vaccination and impact the $CD4^+$ T cell repertoire. By contrast, $CD8^+$ T cell responses were unchanged, suggesting that $CD4^+$ T cell help to $CD8^+$ T cells may not be a primary limiting factor under these RNA vaccination conditions. Our findings of pre-existing immunity enhancing spike-specific $CD4^+$ T cell, T_{FH}, and antibody responses after immunization with an RNA vaccine do not represent the exact scenario of SARS-CoV-2 infection. It therefore remains unresolved whether pre-existing T cells have a biological function during human SARS-CoV-2 infection (*33*). Nevertheless, these data provide evidence that the cross-reactive $CD4^+$ T cells are biologically relevant in the context of vaccination. Thus, it is plausible that the presence and magnitude of cross-reactive memory T cells could accelerate the speed and magnitude of $CD4^+$ T cell and antibody responses to SARS-CoV-2 infection, compared to persons who have undetectable levels of cross-reactive memory T cells. Moreover, early T cell responses have been linked to less severe COVID-19 clinical outcomes (*46*, *47*). In conclusion, these findings show substantial immune responses and immune memory to a low-dose RNA vaccine and indicate biological relevance of cross-reactive memory T cells.

Materials and methods

Human subjects

Samples from the phase 1 25-μg mRNA-1273 SARS-CoV-2 trial
A total of 140 peripheral blood samples were obtained from 35 participants who received the 25-μg dose mRNA-1273 SARS-CoV-2 vaccine in a dose escalation, open-label phase 1 trial (mRN-1273 ClinicalTrials.gov number NCT04283461) (*9*). Participants received two injections of the trial vaccine 28 days apart between March and April of 2020 at one of the two study sites, Kaiser Permanente Washington Health Research Institute in Seattle or at the Emory University School of Medicine in Atlanta. Peripheral blood mononuclear cell (PBMC) samples used in this study were collected at the start of the trial (day 1), 14 days after each vaccination (day 15 ± 2 and 43 ± 2) and 7 months after the first dose (day 209 ± 7).

Participants were grouped into three age groups ranging from 18 to 55 (n = 15), 56 to 70 (n = 10), and >70 years (n = 10) of age (table S3). Two participants from the 18-55 group received only one dose of the 25-μg mRNA-1273 doses. The day 40 and day 209 samples (both collected post second dose) were excluded from analysis for these two vaccinees. Both sexes were represented (20:15, M:F) and the average age of the participants was 58 years. Participants identified as white (n = 34), Hispanic (n = 1) or mixed race (Asian/Hispanic, n = 1.) An overview of samples analyzed in this study is provided in table S3.

The trial was reviewed and approved by the Advarra institutional review board as previously published (*9*). All experiments performed at the La Jolla Institute (LJI) were approved by the institutional review boards (IRB) of the La Jolla Institute (IRB#: VD-214). Collection and processing of vaccinee PBMC samples was performed at one of the two study location sites as previously described (*9*).

Samples from convalescent COVID-19 donors
To compare levels of immune memory responses induced by 25-μg mRNA-1273 SARS-CoV-2 vaccination to immune memory responses induced by natural infection with SARS-CoV-2, we collected blood from individuals that experienced natural infection with SARS-CoV-2. We matched the 7-month (209-day) postvaccination samples with samples from convalescent donors collected on average 181 days (range 170-195) post symptoms onset (PSO). Assuming an average incubation period of 12 days until symptom onset the timepoint post exposure or vaccination is comparable between the cohorts (*48*). To further match ethnicities between the cohorts, we selected 13 samples from Caucasian donors and one sample from a Hispanic donor. Convalescent donors were California residents, who were either referred to the study by a health care provider or self-referred. In the overall cohort, both sexes were represented (10:4, M:F) and the average age of the donors was 35 years (±16.53). Details of this COVID-19 convalescent cohort are listed in table S4. All of the convalescent donors experienced mild illness, defined as patients with a SARS-CoV-2 positive test who have never been hospitalized (*46*). Seropositivity against SARS-CoV-2 was confirmed by ELISA, as describe below. At the time of enrollment, all COVID-19 convalescent donors provided informed consent to participate in the present and future studies.

100-μg mRNA-1273 vaccinees
In addition, we included a cohort of individuals vaccinated locally in San Diego, California, who received the emergency use authorization (EUA)–approved 100-μg mRNA-1273 SARS-CoV-2 vaccine. Samples from 20 vaccinees collected 42 ± 6 days after first immunization (15 days after second immunization) were compared with samples from the 25-μg mRNA-1273 cohort on day 43 ± 2. To match ethnicities between the cohorts, we selected 12 samples from Caucasian donors, 4 samples from Asian donors, and 4 samples from Hispanic donors. In the overall cohort, both sexes were represented (6:14, M:F) and the average age of the donors was 48 years (±14.45). Details of this cohort are listed in table S5. At the time of enrollment, all 100-μg mRNA-1273 SARS-CoV-2 vaccinees provided informed consent to participate in the present and future studies.

Peripheral blood mononuclear cells (PBMCs) and plasma isolation
Whole blood samples from convalescent COVID-19 donors and 100-μg mRNA-1273 vaccinees were collected at La Jolla Institute in heparin-coated blood bags and centrifuged for 15 min at 803*g* to separate the cellular fraction and plasma. The plasma was then carefully removed from the cell pellet and stored at −20°C. PBMCs were isolated by density-gradient sedimentation using Ficoll-Paque (Lymphoprep, Nycomed Pharma, Oslo, Norway) as previously described (*22*, *31*). Isolated PBMCs were cryopreserved in cell recovery media containing 10% dimethyl sulfoxide (DMSO) (Gibco), supplemented with 10% heat-inactivated fetal bovine serum (FBS; Hyclone Laboratories, Logan UT), and stored in liquid nitrogen until used in the assays.

Antibody measurements

SARS-CoV-2 ELISAs
SARS-CoV-2 ELISA titers in vaccinated and convalescent samples were determined as previously described (*22*, *31*, *46*, *49*). Endpoint titers (ET) were plotted for each sample, using background subtracted data, with ET calculated at the dilution giving a reading above the OD cutoff of 0.1. Negative and positive controls were used to standardize each assay and

normalize across experiments. A positive control standard was created by pooling plasma from six convalescent COVID-19 donors to normalize between experiments. The limit of detection (LOD) was defined as 1:3 for IgG. The limit of quantification (LOQ) for vaccinated individuals was established based on uninfected subjects, using plasma from healthy donors never exposed to or vaccinated against SARS-CoV-2.

Pseudovirus (PSV)–neutralization assay

The PSV-neutralization assays of vaccinated and convalescent samples were performed as previously described (*22*). Neutralization titers or inhibition dose 50 (ID_{50}) were calculated using the One-Site Fit Log IC50 model in Prism 9.2 (GraphPad). As internal quality control to define the inter-assay variation, three samples were included across the PSV-neutralization assays. Samples that did not reach 50% inhibition at the lowest serum dilution of 1:20 were considered to be non-neutralizing and the values were set to 19. PSV neutralization titers were performed as two independent experiments on different days with two replicates per experiment. Results were comparable between experiments and results from the first experiment are graphed. We included the WHO International Reference Panel for anti-SARS-Cov2 immunoglobulin (20/268) to calibrate our PSV neutralization titers. The ID_{50} of WHO-High and WHO-Mid were measured by four independent experiments with two replicates per experiment. The geometric means (GMTs) of ID_{50} of WHO-High and WHO-Mid were 2658 and 364, respectively, by our PSV neutralization assay. The WHO assigned neutralization activity unitage of 1473 and 210 IU/ml for the WHO-High and WHO-Mid standards. The calibration factor was thus calculated as ((2658/1473) + (364/ 210))/ 2 = 1.766. The GMTs of PSV neutralization ID_{50} in 25 μg and 100 μg mRNA-1273 vaccinees at day 43 were 997 and 2,000, respectively in our figures. The WHO IU calibrated neutralization ID_{50} (cID_{50}) GMTs in 25 μg mRNA-1273 vaccinees at day 43 would be 565 IU (997/1.766), and for 100 μg mRNA-1273 vaccinees at day 43 would be 1,133 IU (2000/1.766). The limit of detection was calculated as 10.7 IU (19/1.766).

Peptide megapools (MP)

We have previously developed the MP approach to allow simultaneous testing of a large number of epitopes (*22*, *31*, *46*, *50*). Here three MPs to evaluate the antigen-specific T cell response against SARS-CoV-2 were used, as described below. Cytomegalovirus (CMV) MP was used as a control against a ubiquitous pathogen in the experiments.

SARS-CoV-2 MPs

To characterize SARS-CoV-2–specific T cell response, we utilized three MPs previously described (*31*, *43*). First, we used a spike MP of 253 overlapping peptides spanning the entire sequence of the spike protein. As this peptide pool consists of peptides with a length of 15 amino acids, both $CD4^+$ and $CD8^+$ T cells have the capacity to recognize this MP (*51*). In addition, to confirm that the spike-specific $CD8^+$ T cell response observed in the mRNA-1273 vaccinees is also induced in the absence of the spike-specific $CD4^+$ T cell response, we performed some experiments with an optimal MP of HLA Class I epitopes (CD8-S MP). This CD8-S MP consists of 197 9- and 10-mers derived spike peptides that have previously been described to be recognized by $CD8^+$ T cells in SARS-CoV-2 exposed donors (*22*, *31*, *46*, *52*). Lastly, we used a predicted SARS-CoV-2 specific MP (CD4-R) to evaluate the non-spike response or the remainder of the SARS-CoV-2 genome of 246 HLA Class-II $CD4^+$ epitopes previously described (*31*). We have previously shown that these MPs are suitable to stimulate T cell responses from either COVID-19 exposed or SARS-CoV-2-unexposed individuals (*31*, *34*).

Cytomegalovirus (CMV) MP

As a control, we utilized a MP of 313 experimentally defined epitopes. This CMV MP consists of HLA Class I and Class II epitopes and $CD4^+$ and $CD8^+$ T cells have the capacity to recognize this MP, as have been previously published (*50*).

Flow cytometry assays

Activation-induced markers (AIM) assay

Antigen-specific $CD4^+$ T cells were measured as a percentage of AIM^+ ($OX40^+CD137^+$) $CD4^+$ and ($CD69^+CD137^+$) $CD8^+$ T cells after stimulation of PBMCs from mRNA-1273 vaccinees and COVID-19 convalescent donors with peptide MPs. Antigen-specific circulating T follicular helper (cT_{FH}) cells ($CXCR5^+OX40^+CD40L^+$, as percentage of $CD4^+$ T cells) were defined by the AIM assay.

Prior to addition of MPs, cells were blocked at 37°C for 15 min with 0.5 μg/ml of anti-CD40 mAb (Miltenyi Biotec). Then, cells were incubated at 37°C for 24 hours in the presence of fluorescently labeled anti-chemokine receptor antibodies (anti-CCR4, -CCR6, -CCR7, -CXCR3, and -CXCR5) and SARS-CoV-2 MPs (1 μg/ml) or CMV MP (1 μg/ml) in 96-well U-bottom plates, as previously described (*31*, *46*). In addition, PBMCs were incubated with an equimolar amount of DMSO as negative control and with phytohemagglutinin (5 μg/ml) (PHA, Roche) as a positive control.

For the surface stain, 1×10^6 PBMCs were resuspended in PBS, incubated with BD human FC block (BD Biosciences, San Diego, CA) and the LIVE/DEAD marker in the dark for 15 min and washed with PBS. Then, an antibody mix containing the rest of the surface antibodies was added directly to cells and incubated for 60 min at 4°C in the dark. Following surface staining, cells were then washed twice with PBS

containing 3% FBS (FACS buffer). All samples were acquired on a Cytek Aurora (Cytek Biosciences, Fremont, CA). A list of antibodies used in this panel can be found in table S6 and a representative gating strategy of spike-specific $CD4^+$ and $CD8^+$ T cells using the AIM assay is shown in fig. S2.

Antigen-specific $CD4^+$ and $CD8^+$ T cells were measured as background (DMSO)–subtracted data, with a minimal DMSO level set to 0.005%. Response >0.02% and a stimulation index (SI) >2 for $CD4^+$ and >0.03% and SI >3 for $CD8^+$ T cells were considered positive. The LOQ for antigen-specific $CD4^+$ T cell responses (0.03%) and antigen-specific $CD8^+$ T cell responses (0.06%) was calculated using the median twofold standard deviation of all negative controls. As an internal quality control to define inter-assay variation, the CMV-specific $CD4^+$ and $CD8^+$ T cell responses were evaluated for a SARS-CoV-2–unexposed donor included in each independent experiments. The antigen-specific response against CMV and the response to the positive control was compared across experiments and revealed a coefficient of variation (CV) between 10 to 13% for the antigen-specific stimulation with the CMV-MP and a CV between 6 to 12% for mitogenic stimulation with PHA (fig. S2).

Intracellular cytokine staining (ICS) assay

To optimize spike-specific detection of cytokine-producing $CD4^+$ and $CD8^+$ T cells, we experimented with different incubation times (5, 6, and 24 hours) in the presence of GolgiPlug containing brefeldin A and GolgiStop containing monensin (BD Biosciences, San Diego, CA) for an additional 1, 3 or 4 hours, respectively. To establish optimal conditions for the ICS assay, we evaluated the IFNγ-producing $CD4^+$ and $CD8^+$ T cells in 100-μg mRNA-1273 vaccinees (n = 4) and COVID-19 convalescent donors (n = 4) (table S7). The highest signal of IFNγ-producing $CD4^+$ and $CD8^+$ T cells was detected after 24+4 hours incubation in both, vaccinees and convalescent donors. Thus, we chose 24 hours as the best condition to identify spike-specific $CD4^+$ and $CD8^+$ T cells producing intracellular cytokines.

Prior to the addition of MPs, cells were blocked at 37°C for 15 min with 0.5 μg/ml of anti-CD40 mAb, as previously described (*46*). PBMCs were cultured in the presence of SARS-CoV-2 MPs (1 μg/ml) for 24 hours at 37°C. In addition, PBMCs were incubated with an equimolar amount of DMSO as negative control and also CMV MP (1 μg/ml) as a positive control. After 24 hours, Golgi-Plug and Golgi-Stop were added to the culture for 4 hours, as described above. Cells were then washed and surface stained for 30 min at 4°C in the dark and fixed with 1% of paraformaldehyde (Sigma-Aldrich, St. Louis, MO). Antibodies used in the ICS assay are listed in table S8 and a representative gating strategy of spike-specific $CD4^+$ and $CD8^+$ T cells using the ICS assay is shown in fig. S4.

Antigen-specific $CD4^+$ and $CD8^+$ T cells were measured as background (DMSO) subtracted data, with a minimal DMSO level set to 0.001%.

Responses >0.005% and a SI >2 for $CD4^+$ and $CD8^+$ T cells was considered positive. The limit of quantification for antigen-specific $CD4^+$ and $CD8^+$ T cell responses (0.01%) was calculated using the median twofold standard deviation of all negative controls. As internal quality control to define inter assay variation, the CMV-specific $CD4^+$ and $CD8^+$ T cell responses were evaluated for a SARS-CoV-2-unexposed donor included in each independent experiment. The antigen-specific response of $CD4^+$ and $CD8^+$ T cells against CMV were compared across experiments and revealed a CV of 14% for the antigen-specific stimulation with the CMV MP (fig. S4E).

The gates applied for the identification of $CD4^+$ and $CD8^+$ T cells producing cytokines were defined according to the cells cultured with DMSO for each individual as is shown in fig. S4. A Boolean analysis was performed to define the multifunctional profiles on FlowJo 10.7.1. The analysis included CD40L, GzB, IFNγ, IL-2, and TNFα gated on $CD3^+CD4^+$ cells and GzB, IFNγ, IL-2, and TNFα gated on $CD3^+CD8^+$ cells. The overall response to spike was defined as the sum of the background subtracted responses to each combination of individual cytokines. To define the multifunctional profiles of antigen-specific T cells, all positive background-subtracted data (>0.005% and a SI >2 for $CD4^+$ T cells and >0.002% and a SI >2 for $CD8^+$ T cells) was aggregated into a combined sum of antigen-specific $CD4^+$ or $CD8^+$ T cells based on the number of functions. Values higher than the LOQ (0.01%) were considered for the analysis of the multifunctional antigen-specific T cell responses. The average of the relative $CD4^+$ and $CD8^+$ T cell response was calculated per group to define the proportion of multifunctional antigen-specific T cell responses (figs. S6 and S7 and tables S1 and S2).

Statistical analysis

Data was analyses using FlowJo 10.7.1. Statistical analyses were performed in GraphPad Prism 9.2, unless otherwise stated. The statistical details of the experiments are provided in the respective figure legends. Data plotted in linear scale were expressed as means ± standard deviations (SD). Data plotted in logarithmic scales were expressed as geometric means ± geometric standard deviations (SD). Mann–Whitney *U* or Wilcoxon tests were applied for unpaired or paired comparisons, respectively. Differences among age groups were evaluated using Kruskal–Wallis and Dunn's posttest for multiple comparisons. Details pertaining to significance are also noted in the respective legends.

REFERENCES AND NOTES

1. F. P. Polack, S. J. Thomas, N. Kitchin, J. Absalon, A. Gurtman, S. Lockhart, J. L. Perez, G. Pérez Marc, E. D. Moreira, C. Zerbini, R. Bailey, K. A. Swanson, S. Roychoudhury, K. Koury, P. Li, W. V. Kalina, D. Cooper, R. W. Frenck Jr., L. L. Hammitt, Ö. Türeci, H. Nell, A. Schaefer, S. Ünal, D. B. Tresnan, S. Mather, P. R. Dormitzer, U. Şahin, K. U. Jansen, W. C. Gruber; C4591001 Clinical Trial Group, Safety and efficacy of the BNT162b2 mRNA Covid-19 vaccine. *N. Engl. J. Med.* **383**, 2603–2615 (2020). doi:10.1056/NEJMoa2034577 Medline
2. L. R. Baden, H. M. El Sahly, B. Essink, K. Kotloff, S. Frey, R. Novak, D. Diemert, S. A. Spector, N. Rouphael, C. B. Creech, J. McGettigan, S. Khetan, N. Segall, J. Solis, A. Brosz, C. Fierro, H. Schwartz, K. Neuzil, L. Corey, P. Gilbert, H. Janes, D. Follmann, M. Marovich, J. Mascola, L. Polakowski, J. Ledgerwood, B. S. Graham, H. Bennett, R. Pajon, C. Knightly, B. Leav, W. Deng, H. Zhou, S. Han, M. Ivarsson, J. Miller, T. Zaks; COVE Study Group, Efficacy and safety of the mRNA-1273 SARS-CoV-2 vaccine. *N. Engl. J. Med.* **384**, 403–416 (2021). doi:10.1056/NEJMoa2035389 Medline
3. R. L. Soiza, C. Scicluna, E. C. Thomson, Efficacy and safety of COVID-19 vaccines in older people. *Age Ageing* **50**, 279–283 (2021). doi:10.1093/ageing/afaa274 Medline
4. C. Pawlowski, P. Lenehan, A. Puranik, V. Agarwal, A. J. Venkatakrishnan, M. J. M. Niesen, J. C. O'Horo, A. Virk, M. D. Swift, A. D. Badley, J. Halamka, V. Soundararajan, FDA-authorized mRNA COVID-19 vaccines are effective per real-world evidence synthesized across a multi-state health system. *Med* **2**, 979–992.e8 (2021). doi:10.1016/j.medj.2021.06.007 Medline
5. S. Amit, G. Regev-Yochay, A. Afek, Y. Kreiss, E. Leshem, Early rate reductions of SARS-CoV-2 infection and COVID-19 in BNT162b2 vaccine recipients. *Lancet* **397**, 875–877 (2021). doi:10.1016/S0140-6736(21)00448-7 Medline
6. R. W. Frenck Jr., N. P. Klein, N. Kitchin, A. Gurtman, J. Absalon, S. Lockhart, J. L. Perez, E. B. Walter, S. Senders, R. Bailey, K. A. Swanson, H. Ma, X. Xu, K. Koury, W. V. Kalina, D. Cooper, T. Jennings, D. M. Brandon, S. J. Thomas, Ö. Türeci, D. B. Tresnan, S. Mather, P. R. Dormitzer, U. Şahin, K. U. Jansen, W. C. Gruber; C4591001 Clinical Trial Group, Safety, immunogenicity, and efficacy of the BNT162b2 Covid-19 vaccine in adolescents. *N. Engl. J. Med.* **385**, 239–250 (2021). doi:10.1056/NEJMoa2107456 Medline
7. V. J. Hall, S. Foulkes, A. Saei, N. Andrews, B. Oguti, A. Charlett, E. Wellington, J. Stowe, N. Gillson, A. Atti, J. Islam, I. Karagiannis, K. Munro, J. Khawam, M. A. Chand, C. S. Brown, M. Ramsay, J. Lopez-Bernal, S. Hopkins; SIREN Study Group, COVID-19 vaccine coverage in health-care workers in England and effectiveness of BNT162b2 mRNA vaccine against infection (SIREN): A prospective, multicentre, cohort study. *Lancet* **397**, 1725–1735 (2021). doi:10.1016/S0140-6736(21)00790-X Medline
8. D. Wrapp, N. Wang, K. S. Corbett, J. A. Goldsmith, C.-L. Hsieh, O. Abiona, B. S. Graham, J. S. McLellan, Cryo-EM structure of the 2019-nCoV spike in the prefusion conformation. *Science* **367**, 1260–1263 (2020). doi:10.1126/science.abb2507 Medline
9. L. A. Jackson, E. J. Anderson, N. G. Rouphael, P. C. Roberts, M. Makhene, R. N. Coler, M. P. McCullough, J. D. Chappell, M. R. Denison, L. J. Stevens, A. J. Pruijssers, A. McDermott, B. Flach, N. A. Doria-Rose, K. S. Corbett, K. M. Morabito, S. O'Dell, S. D. Schmidt, P. A. Swanson 2nd, M. Padilla, J. R. Mascola, K. M. Neuzil, H. Bennett, W. Sun, E. Peters, M. Makowski, J. Albert, K. Cross, W. Buchanan, R. Pikaart-Tautges, J. E. Ledgerwood, B. S. Graham, J. H. Beigel; mRNA-1273 Study Group, An mRNA vaccine against SARS-CoV-2 — Preliminary report. *N. Engl. J. Med.* **383**, 1920–1931 (2020). doi:10.1056/NEJMoa2022483 Medline
10. S. J. Thomas, E. D. Moreira Jr., N. Kitchin, J. Absalon, A. Gurtman, S. Lockhart, J. L. Perez, G. Pérez Marc, F. P. Polack, C. Zerbini, R. Bailey, K. A. Swanson, X. Xu, S. Roychoudhury, K. Koury, S. Bouguermouh, W. V. Kalina, D. Cooper, R. W. Frenck Jr., L. L. Hammitt, Ö. Türeci, H. Nell, A. Schaefer, S. Ünal, Q. Yang, P. Liberator, D. B. Tresnan, S. Mather, P. R. Dormitzer, U. Şahin, W. C. Gruber, K. U. Jansen; C4591001 Clinical Trial Group, Six month safety and efficacy of the BNT162b2 mRNA COVID-19 vaccine. medRxiv 2021.07.28.21261159 [Preprint]. 28 July 2021. https://doi.org/10.1101/2021.07.28.21261159.
11. Moderna, "Moderna Reports Second Quarter Fiscal Year 2021 Financial Results and Provides Business Updates," press release (5 August 2021); https://investors.modernatx.com/news-releases/news-release-details/moderna-reports-second-quarter-fiscal-year-2021-financial.
12. P. B. Gilbert, D. C. Montefiori, A. McDermott, Y. Fong, D. Benkeser, W. Deng, H. Zhou, C. R. Houchens, K. Martins, L. Jayashankar, F. Castellino, B. Flach, B. C. Lin, S. O'Connell, C. McDanal, A. Eaton, M. Sarzotti-Kelsoe, Y. Lu, C. Yu, B. Borate, L. W. P. van der Laan, N. Hejazi, C. Huynh, J. Miller, H. M. El Sahly, L. R. Baden, M. Baron, L. De La Cruz, C. Gay, S. Kalams, C. F. Kelley, M. Kutner, M. P. Andrasik, J. G. Kublin, L. Corey, K. M. Neuzil, L. N. Carpp, R. Pajon, D. Follmann, R. O. Donis, R. A. Koup, Immune correlates analysis of the mRNA-1273 COVID-19 vaccine efficacy trial. medRxiv 2021.08.09.21261290 [Preprint]. 12 August 2021. https://doi.org/10.1101/2021.08.09.21261290.
13. D. S. Khoury, D. Cromer, A. Reynaldi, T. E. Schlub, A. K. Wheatley, J. A. Juno, K. Subbarao, S. J. Kent, J. A. Triccas, M. P. Davenport, Neutralizing antibody levels are highly predictive of immune protection from symptomatic SARS-CoV-2 infection. *Nat. Med.* **27**, 1205–1211 (2021). doi:10.1038/s41591-021-01377-8 Medline
14. A. Tauzin, M. Nayrac, M. Benlarbi, S. Y. Gong, R. Gasser, G. Beaudoin-Bussières, N. Brassard, A. Laumaea, D. Vézina, J. Prévost, S. P. Anand, C. Bourassa, G. Gendron-Lepage, H. Medjahed, G. Goyette, J. Niessl, O. Tastet, L. Gokool, C. Morrisseau, P. Arlotto, L. Stamatatos, A. T. McGuire, C. Larochelle, P. Uchil, M. Lu, W. Mothes, G. De Serres, S. Moreira, M. Roger, J. Richard, V. Martel-Laferrière, R. Duerr, C. Tremblay, D. E. Kaufmann, A. Finzi, A single dose of the SARS-CoV-2 vaccine BNT162b2 elicits Fc-mediated antibody effector functions and T cell responses. *Cell Host Microbe* **29**, 1137–1150.e6 (2021). doi:10.1016/j.chom.2021.06.001 Medline
15. A. Sette, S. Crotty, Adaptive immunity to SARS-CoV-2 and COVID-19. *Cell* **184**, 861–880 (2021). doi:10.1016/j.cell.2021.01.007 Medline
16. L. J. Abu-Raddad, H. Chemaitelly, P. Coyle, J. A. Malek, A. A. Ahmed, Y. A. Mohamoud, S. Younuskunju, H. H. Ayoub, Z. Al Kanaani, E. Al Kuwari, A. A. Butt, A. Jeremijenko, A. H. Kaleeckal, A. N. Latif, R. M. Shaik, H. F. Abdul Rahim, G. K. Nasrallah, H. M. Yassine, M. G. Al Kuwari, H. E. Al Romaihi, M. H. Al-Thani, A. Al Khal, R. Bertollini, SARS-CoV-2 antibody-positivity protects against reinfection for at least seven months with 95% efficacy. *EClinicalMedicine* **35**, 100861 (2021). doi:10.1016/j.eclinm.2021.100861 Medline
17. S. F. Lumley, D. O'Donnell, N. E. Stoesser, P. C. Matthews, A. Howarth, S. B. Hatch, B. D. Marsden, S. Cox, T. James, F. Warren, L. J. Peck, T. G. Ritter, Z. de Toledo, L. Warren, D. Axten, R. J. Cornall, E. Y. Jones, D. I. Stuart, G. Screaton, D. Ebner, S. Hoosdally, M. Chand, D. W. Crook, A.-M. O'Donnell, C. P. Conlon, K. B. Pouwels, A. S. Walker, T. E. A. Peto, S. Hopkins, T. M. Walker, K. Jeffery, D. W. Eyre; Oxford University Hospitals Staff Testing Group, Antibody status and incidence of SARS-CoV-2 infection in health care workers. *N. Engl. J. Med.* **384**, 533–540 (2021). doi:10.1056/NEJMoa2034545 Medline
18. V. J. Hall, S. Foulkes, A. Charlett, A. Atti, E. J. M. Monk, R. Simmons, E. Wellington, M. J. Cole, A. Saei, B. Oguti, K. Munro, S. Wallace, P. D. Kirwan, M. Shrotri, A. Vusirikala, S. Rokadiya, M. Kall, M. Zambon, M. Ramsay, T. Brooks, C. S. Brown, M. A. Chand, S. Hopkins,; SIREN Study Group, SARS-CoV-2 infection rates of antibody-positive compared with antibody-negative health-care workers in England: A large, multicentre, prospective cohort study (SIREN). *Lancet* **397**, 1459–1469 (2021). doi:10.1016/S0140-6736(21)00675-9 Medline
19. A. Leidi, F. Koegler, R. Dumont, R. Dubos, M. E. Zaballa, G. Piumatti, M. Coen, A. Berner, P. Darbellay Farhoumand, P. Vetter, N. Vuilleumier, L. Kaiser, D. Courvoisier, A. S. Azman, I. Guessous, S. Stringhini; SEROCoV-POP study group, Risk of reinfection after seroconversion to SARS-CoV-2: A population-based propensity-score matched cohort study. *Clin. Infect. Dis.* ciab495 (2021). doi:10.1093/cid/ciab495 Medline
20. N. R. Faria, T. A. Mellan, C. Whittaker, I. M. Claro, D. D. S. Candido, S. Mishra, M. A. E. Crispim, F. C. S. Sales, I. Hawryluk, J. T. McCrone, R. J. G. Hulswit, L. A. M. Franco, M. S. Ramundo, J. G. de Jesus, P. S. Andrade, T. M. Coletti, G. M. Ferreira, C. A. M. Silva, E. R. Manuli, R. H. M. Pereira, P. S. Peixoto, M. U. G. Kraemer, N. Gaburo Jr., C. D. C. Camilo, H. Hoeltgebaum, W. M. Souza, E. C. Rocha, L. M. de Souza, M. C. de Pinho, L. J. T. Araujo, F. S. V. Malta, A. B. de Lima, J. D. P. Silva, D. A. G. Zauli, A. C. S. Ferreira, R. P. Schnekenberg, D. J. Laydon, P. G. T. Walker, H. M. Schlüter, A. L. P. Dos Santos, M. S. Vidal, V. S. Del Caro, R. M. F. Filho, H. M. Dos Santos, R. S. Aguiar, J. L. Proença-Modena, B. Nelson, J. A. Hay, M. Monod, X. Miscouridou, H. Coupland, R. Sonabend, M. Vollmer, A. Gandy, C. A. Prete Jr., V. H. Nascimento, M. A. Suchard, T. A. Bowden, S. L. K. Pond, C.-H. Wu, O. Ratmann, N. M. Ferguson, C. Dye, N. J. Loman, P. Lemey, A. Rambaut, N. A. Fraiji, M. D. P. S.

S. Carvalho, O. G. Pybus, S. Flaxman, S. Bhatt, E. C. Sabino, Genomics and epidemiology of the P.1 SARS-CoV-2 lineage in Manaus, Brazil. *Science* **372**, 815–821 (2021). doi:10.1126/science.abh2644 Medline
21. J. Zuo, A. C. Dowell, H. Pearce, K. Verma, H. M. Long, J. Begum, F. Aiano, Z. Amin-Chowdhury, K. Hoschler, T. Brooks, S. Taylor, J. Hewson, B. Hallis, L. Stapley, R. Borrow, E. Linley, S. Ahmad, B. Parker, A. Horsley, G. Amirthalingam, K. Brown, M. E. Ramsay, S. Ladhani, P. Moss, Robust SARS-CoV-2-specific T cell immunity is maintained at 6 months following primary infection. *Nat. Immunol.* **22**, 620–626 (2021). doi:10.1038/s41590-021-00902-8 Medline
22. J. M. Dan, J. Mateus, Y. Kato, K. M. Hastie, E. D. Yu, C. E. Faliti, A. Grifoni, S. I. Ramirez, S. Haupt, A. Frazier, C. Nakao, V. Rayaprolu, S. A. Rawlings, B. Peters, F. Krammer, V. Simon, E. O. Saphire, D. M. Smith, D. Weiskopf, A. Sette, S. Crotty, Immunological memory to SARS-CoV-2 assessed for up to 8 months after infection. *Science* **371**, eabf4063 (2021). doi:10.1126/science.abf4063 Medline
23. R. J. Boyton, D. M. Altmann, Risk of SARS-CoV-2 reinfection after natural infection. *Lancet* **397**, 1161–1163 (2021). doi:10.1016/S0140-6736(21)00662-0 Medline
24. C. Li, D. Yu, X. Wu, H. Liang, Z. Zhou, Y. Xie, T. Li, J. Wu, F. Lu, L. Feng, M. Mao, L. Lin, H. Guo, S. Yue, F. Wang, Y. Peng, Y. Hu, Z. Wang, J. Yu, Y. Zhang, J. Lu, H. Ning, H. Yang, D. Fu, Y. He, D. Zhou, T. Du, K. Duan, D. Dong, K. Deng, X. Zou, Y. Zhang, R. Zhou, Y. Gao, X. Zhang, X. Yang, Twelve-month specific IgG response to SARS-CoV-2 receptor-binding domain among COVID-19 convalescent plasma donors in Wuhan. *Nat. Commun.* **12**, 4144 (2021). doi:10.1038/s41467-021-24230-5 Medline
25. Z. Wang, F. Muecksch, D. Schaefer-Babajew, S. Finkin, C. Viant, C. Gaebler, H.-H. Hoffmann, C. O. Barnes, M. Cipolla, V. Ramos, T. Y. Oliveira, A. Cho, F. Schmidt, J. Da Silva, E. Bednarski, L. Aguado, J. Yee, M. Daga, M. Turroja, K. G. Millard, M. Jankovic, A. Gazumyan, Z. Zhao, C. M. Rice, P. D. Bieniasz, M. Caskey, T. Hatziioannou, M. C. Nussenzweig, Naturally enhanced neutralizing breadth against SARS-CoV-2 one year after infection. *Nature* **595**, 426–431 (2021). doi:10.1038/s41586-021-03696-9 Medline
26. K. W. Cohen, S. L. Linderman, Z. Moodie, J. Czartoski, L. Lai, G. Mantus, C. Norwood, L. E. Nyhoff, V. V. Edara, K. Floyd, S. C. De Rosa, H. Ahmed, R. Whaley, S. N. Patel, B. Prigmore, M. P. Lemos, C. W. Davis, S. Furth, J. B. O'Keefe, M. P. Gharpure, S. Gunisetty, K. Stephens, R. Antia, V. I. Zarnitsyna, D. S. Stephens, S. Edupuganti, N. Rouphael, E. J. Anderson, A. K. Mehta, J. Wrammert, M. S. Suthar, R. Ahmed, M. J. McElrath, Longitudinal analysis shows durable and broad immune memory after SARS-CoV-2 infection with persisting antibody responses and memory B and T cells. *Cell Rep. Med.* **2**, 100354 (2021). doi:10.1016/j.xcrm.2021.100354 Medline
27. A. Pegu, S. O'Connell, S. D. Schmidt, S. O'Dell, C. A. Talana, L. Lai, J. Albert, E. Anderson, H. Bennett, K. S. Corbett, B. Flach, L. Jackson, B. Leav, J. E. Ledgerwood, C. J. Luke, M. Makowski, M. C. Nason, P. C. Roberts, M. Roederer, P. A. Rebolledo, C. A. Rostad, N. G. Rouphael, W. Shi, L. Wang, A. T. Widge, E. S. Yang, J. H. Beigel, B. S. Graham, J. R. Mascola, M. S. Suthar, A. B. McDermott, N. A. Doria-Rose; mRNA-1273 Study Group, Durability of mRNA-1273 vaccine-induced antibodies against SARS-CoV-2 variants. *Science* eabj4176 (2021). doi:10.1126/science.abj4176 Medline
28. E. J. Anderson, N. G. Rouphael, A. T. Widge, L. A. Jackson, P. C. Roberts, M. Makhene, J. D. Chappell, M. R. Denison, L. J. Stevens, A. J. Pruijssers, A. B. McDermott, B. Flach, B. C. Lin, N. A. Doria-Rose, S. O'Dell, S. D. Schmidt, K. S. Corbett, P. A. Swanson 2nd, M. Padilla, K. M. Neuzil, H. Bennett, B. Leav, M. Makowski, J. Albert, K. Cross, V. V. Edara, K. Floyd, M. S. Suthar, D. R. Martinez, R. Baric, W. Buchanan, C. J. Luke, V. K. Phadke, C. A. Rostad, J. E. Ledgerwood, B. S. Graham, J. H. Beigel; mRNA-1273 Study Group, Safety and immunogenicity of SARS-CoV-2 mRNA-1273 vaccine in older adults. *N. Engl. J. Med.* **383**, 2427–2438 (2020). doi:10.1056/NEJMoa2028436 Medline
29. US Food and Drug Administration, "Vaccines and Related Biological Products Advisory Committee Meeting Presentation," 22 October 2020.
30. J. Braun, L. Loyal, M. Frentsch, D. Wendisch, P. Georg, F. Kurth, S. Hippenstiel, M. Dingeldey, B. Kruse, F. Fauchere, E. Baysal, M. Mangold, L. Henze, R. Lauster, M. A. Mall, K. Beyer, J. Röhmel, S. Voigt, J. Schmitz, S. Miltenyi, I. Demuth, M. A. Müller, A. Hocke, M. Witzenrath, N. Suttorp, F. Kern, U. Reimer, H. Wenschuh, C. Drosten, V. M. Corman, C. Giesecke-Thiel, L. E. Sander, A. Thiel, SARS-CoV-2-reactive T cells in healthy donors and patients with COVID-19. *Nature* **587**, 270–274 (2020). doi:10.1038/s41586-020-2598-9 Medline
31. A. Grifoni, D. Weiskopf, S. I. Ramirez, J. Mateus, J. M. Dan, C. R. Moderbacher, S. A. Rawlings, A. Sutherland, L. Premkumar, R. S. Jadi, D. Marrama, A. M. de Silva, A. Frazier, A. F. Carlin, J. A. Greenbaum, B. Peters, F. Krammer, D. M. Smith, S. Crotty, A. Sette, Targets of T cell responses to SARS-CoV-2 coronavirus in humans with COVID-19 disease and unexposed individuals. *Cell* **181**, 1489–1501.e15 (2020). doi:10.1016/j.cell.2020.05.015 Medline
32. N. Le Bert, A. T. Tan, K. Kunasegaran, C. Y. L. Tham, M. Hafezi, A. Chia, M. H. Y. Chng, M. Lin, N. Tan, M. Linster, W. N. Chia, M. I.-C. Chen, L.-F. Wang, E. E. Ooi, S. Kalimuddin, P. A. Tambyah, J. G.-H. Low, Y.-J. Tan, A. Bertoletti, SARS-CoV-2-specific T cell immunity in cases of COVID-19 and SARS, and uninfected controls. *Nature* **584**, 457–462 (2020). doi:10.1038/s41586-020-2550-z Medline
33. M. Lipsitch, Y. H. Grad, A. Sette, S. Crotty, Cross-reactive memory T cells and herd immunity to SARS-CoV-2. *Nat. Rev. Immunol.* **20**, 709–713 (2020). doi:10.1038/s41577-020-00460-4 Medline
34. J. Mateus, A. Grifoni, A. Tarke, J. Sidney, S. I. Ramirez, J. M. Dan, Z. C. Burger, S. A. Rawlings, D. M. Smith, E. Phillips, S. Mallal, M. Lammers, P. Rubiro, L. Quiambao, A. Sutherland, E. D. Yu, R. da Silva Antunes, J. Greenbaum, A. Frazier, A. J. Markmann, L. Premkumar, A. de Silva, B. Peters, S. Crotty, A. Sette, D. Weiskopf, Selective and cross-reactive SARS-CoV-2 T cell epitopes in unexposed humans. *Science* **370**, 89–94 (2020). doi:10.1126/science.abd3871 Medline
35. B. J. Meckiff, C. Ramírez-Suástegui, V. Fajardo, S. J. Chee, A. Kusnadi, H. Simon, S. Eschweiler, A. Grifoni, E. Pelosi, D. Weiskopf, A. Sette, F. Ay, G. Seumois, C. H. Ottensmeier, P. Vijayanand, Imbalance of regulatory and cytotoxic SARS-CoV-2-reactive $CD4^+$ T cells in COVID-19. *Cell* **183**, 1340–1353.e16 (2020). doi:10.1016/j.cell.2020.10.001 Medline
36. A. Sette, S. Crotty, Pre-existing immunity to SARS-CoV-2: The knowns and unknowns. *Nat. Rev. Immunol.* **20**, 457–458 (2020). doi:10.1038/s41577-020-0389-z Medline
37. D. Weiskopf, K. S. Schmitz, M. P. Raadsen, A. Grifoni, N. M. A. Okba, H. Endeman, J. P. C. van den Akker, R. Molenkamp, M. P. G. Koopmans, E. C. M. van Gorp, B. L. Haagmans, R. L. de Swart, A. Sette, R. D. de Vries, Phenotype and kinetics of SARS-CoV-2-specific T cells in COVID-19 patients with acute respiratory distress syndrome. *Sci. Immunol.* **5**, eabd2071 (2020). doi:10.1126/sciimmunol.abd2071 Medline
38. E. M. Anderson, E. C. Goodwin, A. Verma, C. P. Arevalo, M. J. Bolton, M. E. Weirick, S. Gouma, C. M. McAllister, S. R. Christensen, J. Weaver, P. Hicks, T. B. Manzoni, O. Oniyide, H. Ramage, D. Mathew, A. E. Baxter, D. A. Oldridge, A. R. Greenplate, J. E. Wu, C. Alanio, K. D'Andrea, O. Kuthuru, J. Dougherty, A. Pattekar, J. Kim, N. Han, S. A. Apostolidis, A. C. Huang, L. A. Vella, L. Kuri-Cervantes, M. B. Pampena, M. R. Betts, E. J. Wherry, N. J. Meyer, S. Cherry, P. Bates, D. J. Rader, S. E. Hensley; UPenn COVID Processing Unit, Seasonal human coronavirus antibodies are boosted upon SARS-CoV-2 infection but not associated with protection. *Cell* **184**, 1858–1864.e10 (2021). doi:10.1016/j.cell.2021.02.010 Medline
39. M. Sagar, K. Reifler, M. Rossi, N. S. Miller, P. Sinha, L. F. White, J. P. Mizgerd, Recent endemic coronavirus infection is associated with less-severe COVID-19. *J. Clin. Invest.* **131**, e143380 (2021). doi:10.1172/JCI143380 Medline
40. N. Doria-Rose, M. S. Suthar, M. Makowski, S. O'Connell, A. B. McDermott, B. Flach, J. E. Ledgerwood, J. R. Mascola, B. S. Graham, B. C. Lin, S. O'Dell, S. D. Schmidt, A. T. Widge, V.-V. Edara, E. J. Anderson, L. Lai, K. Floyd, N. G. Rouphael, V. Zarnitsyna, P. C. Roberts, M. Makhene, W. Buchanan, C. J. Luke, J. H. Beigel, L. A. Jackson, K. M. Neuzil, H. Bennett, B. Leav, J. Albert, P. Kunwar; mRNA-1273 Study Group, Antibody persistence through 6 months after the second dose of mRNA-1273 vaccine for Covid-19. *N. Engl. J. Med.* **384**, 2259–2261 (2021). doi:10.1056/NEJMc2103916 Medline
41. S. Crotty, T follicular helper cell biology: A decade of discovery and diseases. *Immunity* **50**, 1132–1148 (2019). doi:10.1016/j.immuni.2019.04.011 Medline
42. C. E. Gustafson, C. Kim, C. M. Weyand, J. J. Goronzy, Influence of immune aging on vaccine responses. *J. Allergy Clin. Immunol.* **145**, 1309–1321 (2020). doi:10.1016/j.jaci.2020.03.017 Medline
43. A. Grifoni, J. Sidney, Y. Zhang, R. H. Scheuermann, B. Peters, A. Sette, A sequence homology and bioinformatic approach can predict candidate targets for immune responses to SARS-CoV-2. *Cell Host Microbe* **27**, 671–680.e2 (2020). doi:10.1016/j.chom.2020.03.002 Medline
44. U. Sahin, A. Muik, I. Vogler, E. Derhovanessian, L. M. Kranz, M. Vormehr, J. Quandt, N. Bidmon, A. Ulges, A. Baum, K. E. Pascal, D. Maurus, S. Brachtendorf, V. Lörks,

J. Sikorski, P. Koch, R. Hilker, D. Becker, A.-K. Eller, J. Grützner, M. Tonigold, C. Boesler, C. Rosenbaum, L. Heesen, M.-C. Kühnle, A. Poran, J. Z. Dong, U. Luxemburger, A. Kemmer-Brück, D. Langer, M. Bexon, S. Bolte, T. Palanche, A. Schultz, S. Baumann, A. J. Mahiny, G. Boros, J. Reinholz, G. T. Szabó, K. Karikó, P.-Y. Shi, C. Fontes-Garfias, J. L. Perez, M. Cutler, D. Cooper, C. A. Kyratsous, P. R. Dormitzer, K. U. Jansen, Ö. Türeci, BNT162b2 vaccine induces neutralizing antibodies and poly-specific T cells in humans. *Nature* **595**, 572–577 (2021). doi:10.1038/s41586-021-03653-6 Medline

45. K. S. Corbett, B. Flynn, K. E. Foulds, J. R. Francica, S. Boyoglu-Barnum, A. P. Werner, B. Flach, S. O'Connell, K. W. Bock, M. Minai, B. M. Nagata, H. Andersen, D. R. Martinez, A. T. Noe, N. Douek, M. M. Donaldson, N. N. Nji, G. S. Alvarado, D. K. Edwards, D. R. Flebbe, E. Lamb, N. A. Doria-Rose, B. C. Lin, M. K. Louder, S. O'Dell, S. D. Schmidt, E. Phung, L. A. Chang, C. Yap, J. M. Todd, L. Pessaint, A. Van Ry, S. Browne, J. Greenhouse, T. Putman-Taylor, A. Strasbaugh, T.-A. Campbell, A. Cook, A. Dodson, K. Steingrebe, W. Shi, Y. Zhang, O. M. Abiona, L. Wang, A. Pegu, E. S. Yang, K. Leung, T. Zhou, I.-T. Teng, A. Widge, I. Gordon, L. Novik, R. A. Gillespie, R. J. Loomis, J. I. Moliva, G. Stewart-Jones, S. Himansu, W.-P. Kong, M. C. Nason, K. M. Morabito, T. J. Ruckwardt, J. E. Ledgerwood, M. R. Gaudinski, P. D. Kwong, J. R. Mascola, A. Carfi, M. G. Lewis, R. S. Baric, A. McDermott, I. N. Moore, N. J. Sullivan, M. Roederer, R. A. Seder, B. S. Graham, Evaluation of the mRNA-1273 vaccine against SARS-CoV-2 in nonhuman primates. *N. Engl. J. Med.* **383**, 1544–1555 (2020). doi:10.1056/NEJMoa2024671 Medline
46. C. Rydyznski Moderbacher, S. I. Ramirez, J. M. Dan, A. Grifoni, K. M. Hastie, D. Weiskopf, S. Belanger, R. K. Abbott, C. Kim, J. Choi, Y. Kato, E. G. Crotty, C. Kim, S. A. Rawlings, J. Mateus, L. P. V. Tse, A. Frazier, R. Baric, B. Peters, J. Greenbaum, E. Ollmann Saphire, D. M. Smith, A. Sette, S. Crotty, Antigen-specific adaptive immunity to SARS-CoV-2 in acute COVID-19 and associations with age and disease severity. *Cell* **183**, 996–1012.e19 (2020). doi:10.1016/j.cell.2020.09.038 Medline
47. A. T. Tan, M. Linster, C. W. Tan, N. Le Bert, W. N. Chia, K. Kunasegaran, Y. Zhuang, C. Y. L. Tham, A. Chia, G. J. D. Smith, B. Young, S. Kalimuddin, J. G. H. Low, D. Lye, L.-F. Wang, A. Bertoletti, Early induction of functional SARS-CoV-2-specific T cells associates with rapid viral clearance and mild disease in COVID-19 patients. *Cell Rep.* **34**, 108728 (2021). doi:10.1016/j.celrep.2021.108728 Medline
48. S. A. Lauer, K. H. Grantz, Q. Bi, F. K. Jones, Q. Zheng, H. R. Meredith, A. S. Azman, N. G. Reich, J. Lessler, The incubation period of coronavirus disease 2019 (COVID-19) from publicly reported confirmed cases: Estimation and application. *Ann. Intern. Med.* **172**, 577–582 (2020). doi:10.7326/M20-0504 Medline
49. F. Amanat, D. Stadlbauer, S. Strohmeier, T. H. O. Nguyen, V. Chromikova, M. McMahon, K. Jiang, G. A. Arunkumar, D. Jurczyszak, J. Polanco, M. Bermudez-Gonzalez, G. Kleiner, T. Aydillo, L. Miorin, D. S. Fierer, L. A. Lugo, E. M. Kojic, J. Stoever, S. T. H. Liu, C. Cunningham-Rundles, P. L. Felgner, T. Moran, A. García-Sastre, D. Caplivski, A. C. Cheng, K. Kedzierska, O. Vapalahti, J. M. Hepojoki, V. Simon, F. Krammer, A serological assay to detect SARS-CoV-2 seroconversion in humans. *Nat. Med.* **26**, 1033–1036 (2020). doi:10.1038/s41591-020-0913-5 Medline
50. S. Carrasco Pro, J. Sidney, S. Paul, C. Lindestam Arlehamn, D. Weiskopf, B. Peters, A. Sette, Automatic generation of validated specific epitope sets. *J. Immunol. Res.* **2015**, 763461 (2015). doi:10.1155/2015/763461 Medline
51. M. F. Kotturi, B. Peters, F. Buendia-Laysa Jr., J. Sidney, C. Oseroff, J. Botten, H. Grey, M. J. Buchmeier, A. Sette, The CD8$^+$ T-cell response to lymphocytic choriomeningitis virus involves the L antigen: Uncovering new tricks for an old virus. *J. Virol.* **81**, 4928–4940 (2007). doi:10.1128/JVI.02632-06 Medline
52. A. Grifoni, J. Sidney, R. Vita, B. Peters, S. Crotty, D. Weiskopf, A. Sette, SARS-CoV-2 human T cell epitopes: Adaptive immune response against COVID-19. *Cell Host Microbe* **29**, 1076–1092 (2021). doi:10.1016/j.chom.2021.05.010 Medline

ACKNOWLEDGMENTS

This work used samples from the phase 1 mRNA-1273 study (NCT04283461) (*9*, *28*). The study was conducted in collaboration with ModernaTX and funding for the manufacture of mRNA-1273 phase 1 material was provided by the Coalition for Epidemic Preparedness Innovation. We would like to thank E.O. Saphire for providing the spike plasmids and the LJI Clinical Core for healthy donor enrollment and blood sample procurement. **Funding:** The mRNA-1273 phase 1 study was sponsored and primarily funded by the National Institute of Allergy and Infectious Diseases (NIAID), National Institutes of Health (NIH), Bethesda, MD; in part with federal funds from the NIAID under grant awards UM1AI148373, to Kaiser Washington; UM1AI148576, UM1AI148684, and NIH P51 OD011132, to Emory University; NIH AID AI149644, and contract award HHSN272201500002C, to Emmes. This work was funded by the NIH NIAID under awards AI142742 (Cooperative Centers for Human Immunology) (A.S., S.C.) and NIH contract Nr. 75N9301900065 (D.W., A.S.). This work was additionally supported in part by LJI Institutional Funds and the NIAID under K08 award AI135078 (J.M.D.) **Author contributions:** Conceptualization: A.S., S.C., D.W.; Methodology: J.M., J.M.D., Z.Z., C.R.M., M.L., B.G., D.W.; Formal analysis: J.M., J.M.D., Z.Z., A.S., S.C., D.W.; Investigation: J.M., A.S., S.C., D.W.; Funding acquisition: A.S., S.C., D.W.; Writing: J.M., J.M.D., Z.Z., A.S., S.C., D.W.; Supervision: A.S., S.C., D.W. **Competing interests:** A.S. is a consultant for Gritstone Bio, Flow Pharma, Arcturus Therapeutics, ImmunoScape, CellCarta, Oxford Immunotec, and Avalia Immunotherapies. S.C has consulted for Avalia Immunotherapies, Roche, and GSK. LJI has filed for patent protection for various aspects of T cell epitope and vaccine design work. All other authors declare no conflict of interest. **Data and materials availability:** Epitope pools utilized in this paper are available to the scientific community upon request and execution of a material transfer agreement (MTA). All other data are available in the main text or the supplementary materials. This work is licensed under a Creative Commons Attribution 4.0 International (CC BY 4.0) license, which permits unrestricted use, distribution, and reproduction in any medium, provided the original work is properly cited. To view a copy of this license, visit https://creativecommons.org/licenses/by/4.0/. This license does not apply to figures/photos/artwork or other content included in the article that is credited to a third party; obtain authorization from the rights holder before using such material.

SUPPLEMENTARY MATERIALS

https://science.org/doi/10.1126/science.abj9853
Figs. S1 to S13
Tables S1 to S8
References
MDAR Reproducibility Checklist
Data S1

16 June 2021; accepted 7 September 2021
Published online 14 September 2021
10.1126/science.abj9853

Table 1. Spike-specific immune responses detected in 25 μg mRNA-1273 vaccinees. ELISA, enzyme-linked immunosorbent assay; AIM, activation induced markers; ICS, intracellular cytokine staining.

Component	Assay	Days post-vaccination			
		1	15 ± 2	43 ± 2	209 ± 7
	Antibodies				
Anti-spike IgG	ELISA	0	86%	100%	100%
Anti-RBD IgG	ELISA	3%	94%	100%	100%
Neutralizing	Neutralization	0	29%	100%	88%
	T cells				
Spike-specific $CD4^+$ T cells	AIM*	49%	97%	100%	97%
	ICS†	34%	94%	100%	97%
	Total‡	49%	97%	100%	97%
Spike-specific $CD8^+$ T cells	AIM*	0	34%	53%	34%
	ICS†	0	51%	70%	54%
	Total‡	0	69%	88%	67%

*Antigen-specific T cells using AIM were measured as a percentage $CD4^+$ T expressing $OX40^+CD137^+$ and $CD8^+$ T cells expressing $CD69^+CD137^+$ after stimulation of PBMC with spike overlapping peptides spanning the entire protein. †Antigen-specific T cells using ICS were measured as a percentage $CD4^+$ T expressing CD40L or producing IFNγ, TNFα, IL-2, or GzB; and $CD8^+$ T cells producing IFNγ, TNFα, IL-2, or GzB after stimulation of PBMC with spike overlapping peptides spanning the entire protein. ‡The overall spike-specific T cell response was calculated based on the AIM and ICS results per donor and time-point.

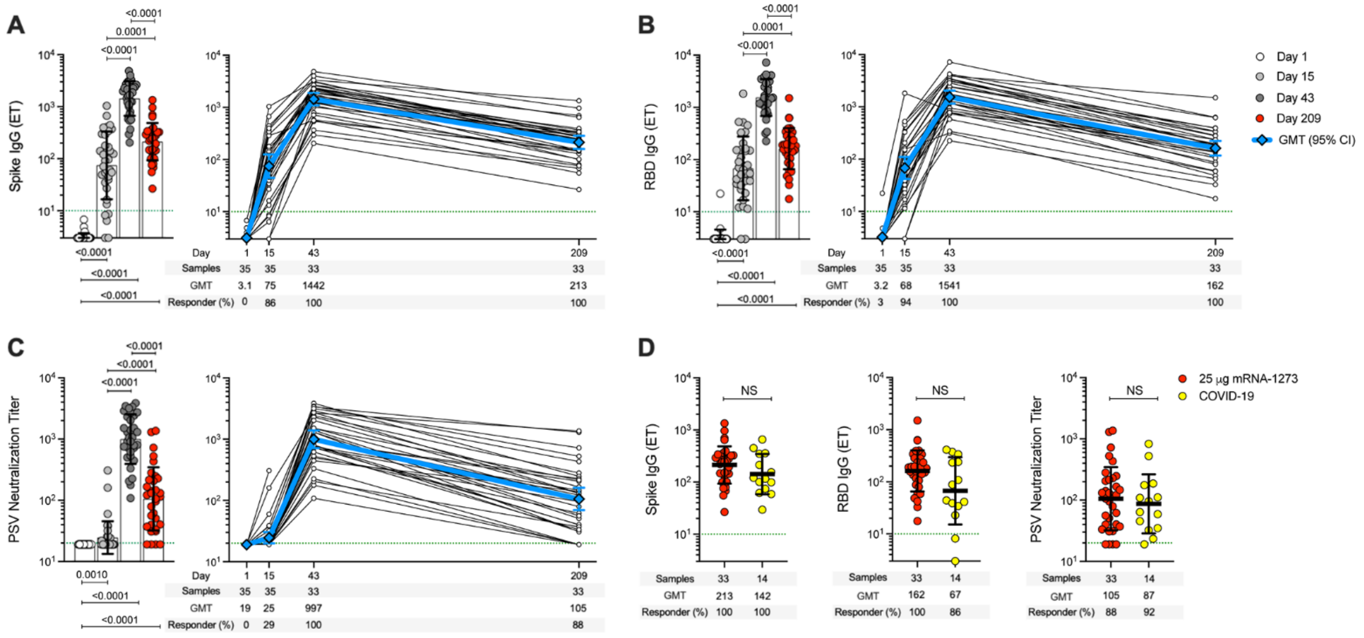

Fig. 1. Spike antibodies induced by 25-μg mRNA-1273 vaccination. Participants received two injections of the 25-μg mRNA-1273 vaccine, 28 days apart. PBMC samples were collected on day 1, day 15 ± 2 (2 weeks post first dose), day 43 ± 2 (2 weeks post second dose), and day 209 ± 7 days (6 months post second dose). (**A**) Longitudinal anti-SARS-CoV-2 spike IgG binding titers, (**B**) Longitudinal anti-SARS-CoV-2 RBD IgG binding titers, and (**C**) Longitudinal SARS-CoV-2 spike pseudovirus-neutralizing titers (PSV). (**D**) Comparison of anti-spike IgG, anti-RBD IgG, and PSV-neutralizing titers induced by two doses of 25-μg mRNA-1273 vaccine at day 209 ± 7 (n = 33) and COVID-19 convalescent donors at 170-195 days PSO (n = 14). Dotted green lines indicate the limit of quantification (LOQ). The bars in (A), (B), (C), and (D) indicate the geometric mean titers (GMT) and geometric SD for anti-spike IgG (Endpoint titers, ET), anti-RBD IgG (ET), and PSV-neutralizing titers, respectively. Data were analyzed for statistical significance using Wilcoxon signed-rank test [(A), (B), and (C)] and Mann–Whitney *U* test (D). NS, non-significant. Background-subtracted and log data analyzed in all cases.

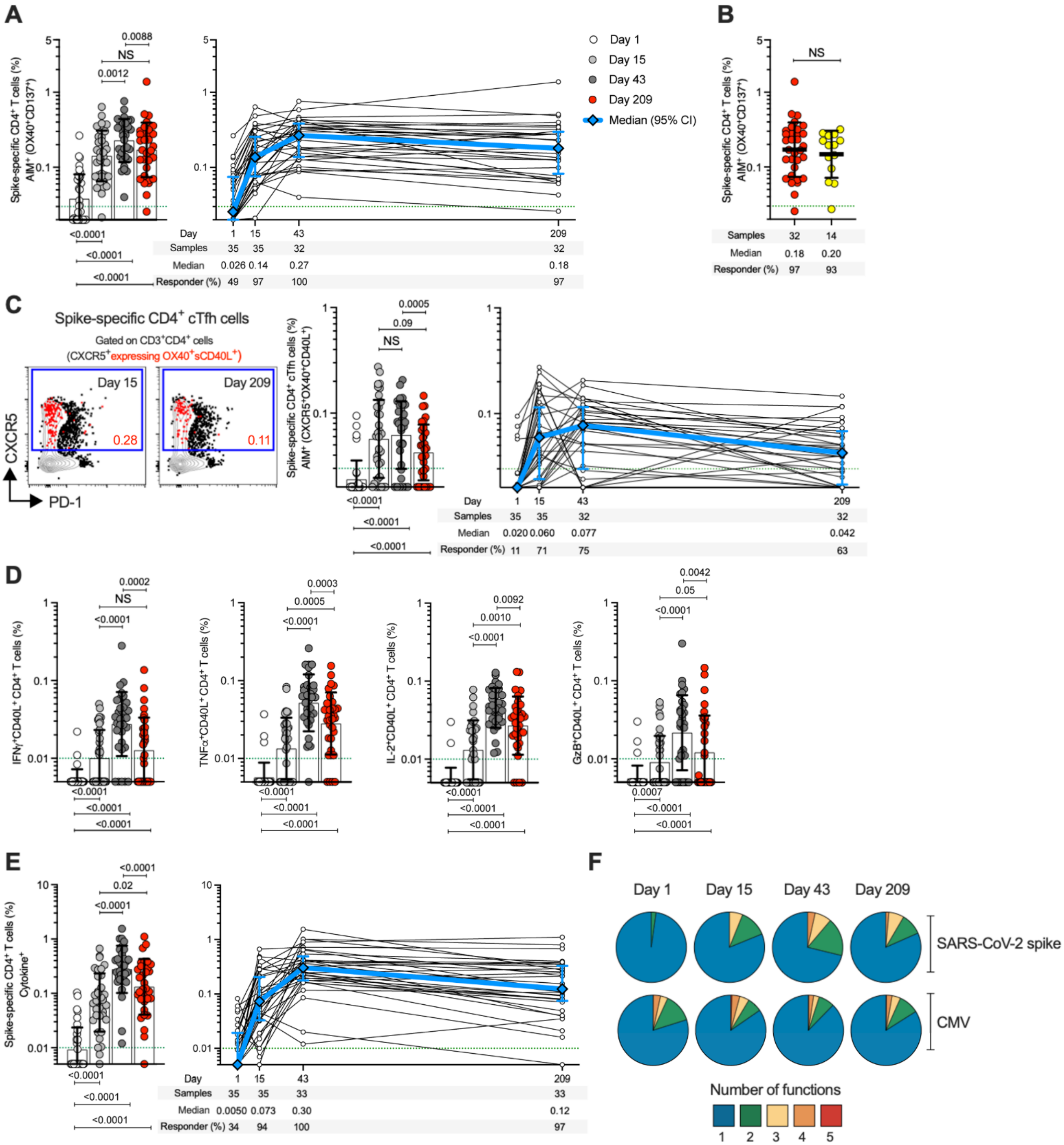
A
B
C
D
E
F
Day 1
Day 15
Day 43
Day 209
Median (95% CI)
Spike-specific CD4+ T cells (%) AIM+ (OX40+CD137+)
Day 1 15 43 209
Samples 35 35 32 32
Median 0.026 0.14 0.27 0.18
Responder (%) 49 97 100 97
Samples 32 14
Median 0.18 0.20
Responder (%) 97 93
Spike-specific CD4+ cTfh cells
Gated on CD3+CD4+ cells
(CXCR5+expressing OX40+sCD40L+)
Day 15
Day 209
CXCR5
PD-1
Spike-specific CD4+ cTfh cells (%) AIM+ (CXCR5+OX40+CD40L+)
Day 1 15 43 209
Samples 35 35 32 32
Median 0.020 0.060 0.077 0.042
Responder (%) 11 71 75 63
IFNγ+CD40L+ CD4+ T cells (%)
TNFα+CD40L+ CD4+ T cells (%)
IL-2+CD40L+ CD4+ T cells (%)
GzB+CD40L+ CD4+ T cells (%)
Spike-specific CD4+ T cells (%) Cytokine+
Day 1 15 43 209
Samples 35 35 33 33
Median 0.0050 0.073 0.30 0.12
Responder (%) 34 94 100 97
SARS-CoV-2 spike
CMV
Number of functions
1 2 3 4 5

Fig. 2. mRNA-1273 vaccination induces durable and multifunctional spike-specific $CD4^+$ T cell responses. (**A**) Longitudinal spike-specific $CD4^+$ T cells in mRNA-1273 vaccinees measured by AIM. Spike-specific $CD4^+$ T cells quantified by AIM (surface $OX40^+CD137^+$) after stimulation with spike megapool (MP) in mRNA-1273 vaccinees (see fig. S2 for gating strategy). (**B**) Comparison of spike-specific AIM^+ $CD4^+$ T cell frequencies between 25-μg mRNA-1273 vaccine at day 209 ± 7 (red circles, n = 32) and COVID-19 convalescent donors at 170-195 days PSO (yellow circles, n = 14). (**C**) Quantitation of spike-specific circulating T follicular helper (cT_{FH}) cells ($CXCR5^+OX40^+$surface $CD40L^+$, as percentage of $CD4^+$ T cells) after stimulation with spike MP. Representative examples of spike-specific cT_{FH} cells (red), overlaid on total $CD4^+$ T cells, at days 15 ± 2 and 209 ± 7. (**D**) Spike-specific $CD4^+$ T cells expressing intracellular CD40L (iCD40L) and producing IFNγ, TNFα, IL-2, or granzyme B (GzB) in mRNA-1273 vaccinees. (**E**) Longitudinal spike-specific $CD4^+$ $cytokine^+$ T cells expressing iCD40L or producing IFNγ, TNFα, IL-2, or GzB in 25-μg mRNA-1273 vaccinees (see fig. S4 for gating strategy). Dotted green lines indicate the limit of quantification (LOQ). Day 1 = white, day 15 ± 2 = light gray, day 43 ± 2 = dark gray, day 209 ± 7 = red. The bars in (A), (B), (C), (D), and (E) indicate the geometric mean and geometric SD in the analysis of the spike-specific $CD4^+$ T cell frequencies. (**F**) Longitudinal multifunctional spike-specific $CD4^+$ T cells in mRNA-1273 vaccinees. Proportions of multifunctional activity profiles of the spike-specific $CD4^+$ T cells from mRNA-1273 vaccinees evaluated on days 1, 15 ± 2, 43 ± 2, and 209 ± 7. The blue, green, yellow, orange, and red colors in the pie charts depict the production of one, two, three, four, and five functions, respectively (see figs. S4 and S6 for details). Data were analyzed for statistical significance using Wilcoxon signed-rank test [(A), (C), (D), and (E)] and Mann–Whitney *U* test (B). NS, non-significant. Background-subtracted and log data analyzed in all cases.

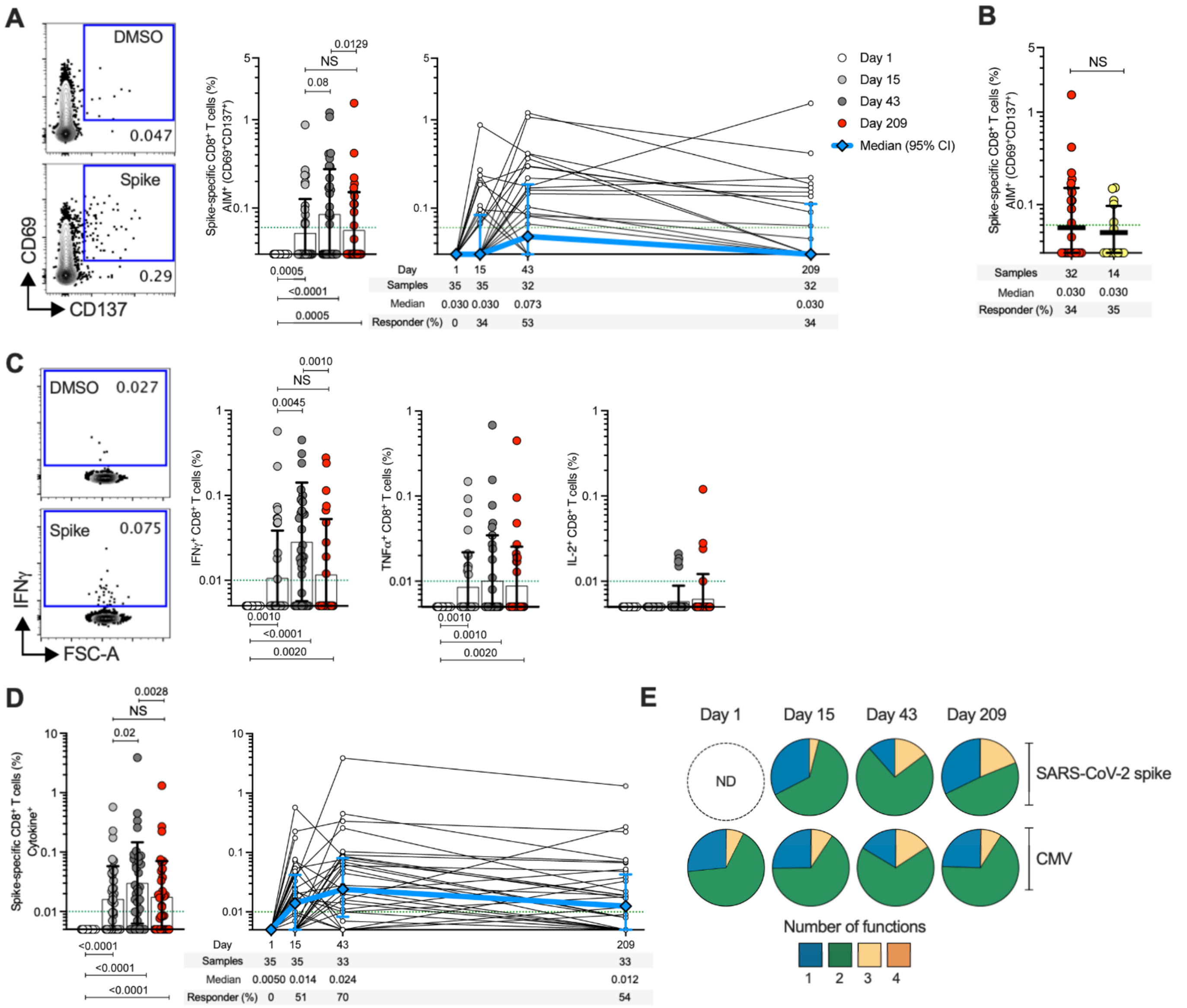

Fig. 3. mRNA-1273 vaccination induces multifunctional spike-specific CD8+ T cells. (**A**) Longitudinal spike-specific CD8+ T cells in mRNA-1273 vaccinees measured by AIM (surface CD69+CD137+). (Left panel) Representative examples of flow cytometry plots of spike-specific CD8+ T cells compared to DMSO control (see fig. S2 for gating strategy). (Right panel) Spike-specific CD8+ T cells quantified. (**B**) Comparison of spike-specific AIM+ CD8+ T cell frequencies between 25-μg mRNA-1273 vaccinees at day 209 ± 7 (n = 32) and COVID-19 convalescent donors at 170-195 days PSO (n = 14). (**C**) Spike-specific CD8+ T cells producing IFNγ, TNFα, or IL-2 by intracellular cytokine staining (ICS) in 25-μg mRNA-1273 vaccinees. (**D**) Longitudinal spike-specific CD8+ cytokine+ T cells producing IFNγ, TNFα, IL-2 or GzB in 25-μg mRNA-1273 vaccinees (see fig. S4 for gating strategy). Dotted green lines indicate the limit of quantification (LOQ). The bars in (A) to (D) indicate the geometric mean and geometric SD. Day 1 = white, day 15 ± 2 = light gray, day 43 ± 2 = dark gray, day 209 ± 7 = red. (**E**) Multifunctional activity profiles of spike-specific CD8+ T cells from 25-μg mRNA-1273 vaccinees, evaluated for IFNγ, TNFα, IL-2, or GzB (see figs. S4 and S8 for details). The blue, green, yellow, and orange colors in the pie charts depict the production of one, two, three, and four functions, respectively. Data were analyzed for statistical significance using Wilcoxon signed-rank test [(A), (C), and (D)] and Mann–Whitney *U* test (B). NS, non-significant. Background-subtracted and log data analyzed in all cases.

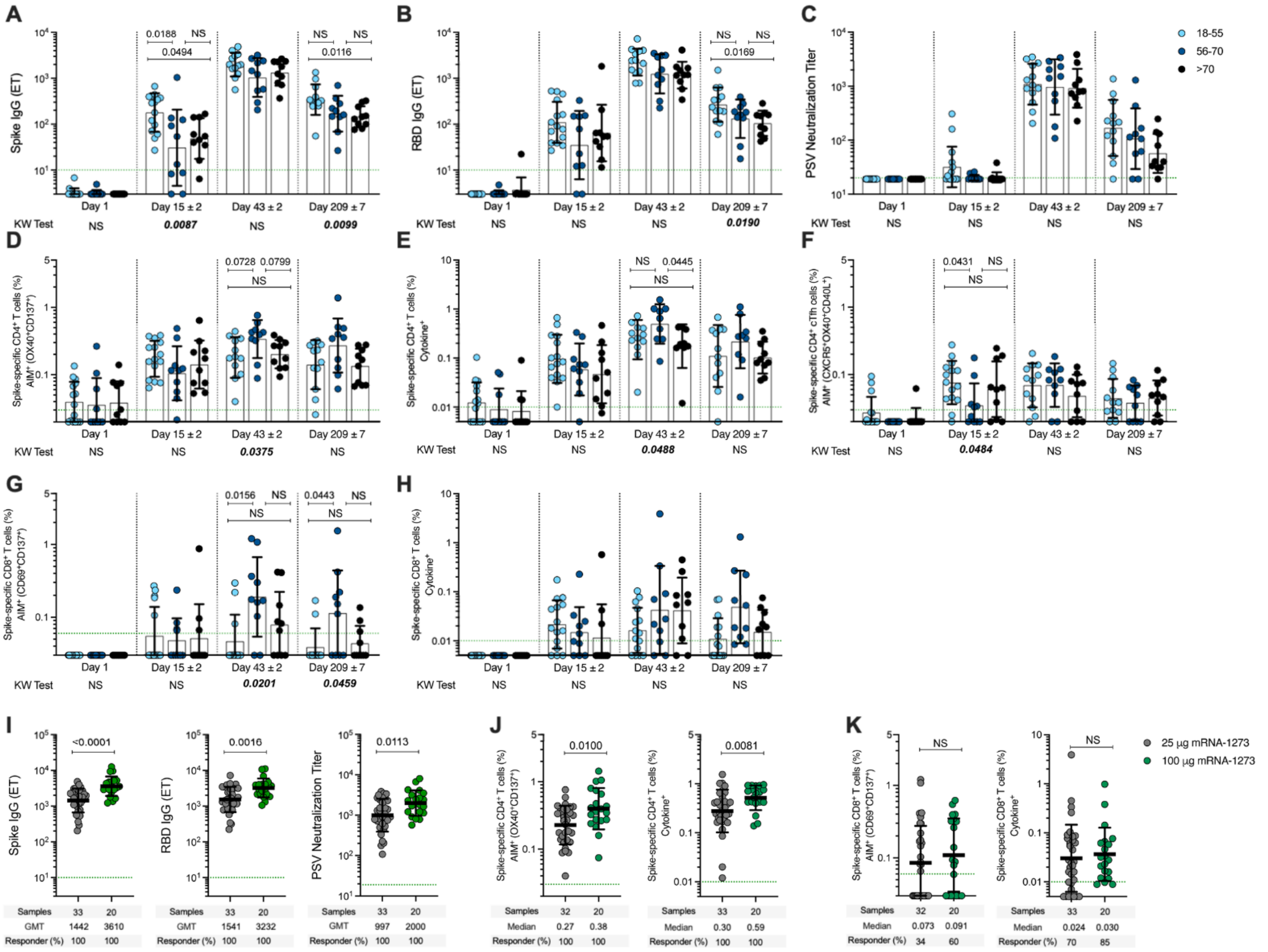

Fig. 4. Spike-specific antibody and T cell responses induced by mRNA-1273 vaccination. (**A** to **H**) Immune responses to 25-μg mRNA-1273 vaccination in three adult age groups: 18-55 (light blue symbols), 56-70 (dark blue), and over 70 years of age (black). [(A) to (C)] Anti-spike IgG, anti-RBD IgG, and PSV-neutralizing titers. [(D) and (E)] Spike-specific CD4$^+$ T cells by AIM (D) or ICS (E). (F) Spike-specific cT$_{FH}$ cells. [(G) and (H)] Spike-specific CD8$^+$ T cells by AIM (G) or ICS (H). Data were analyzed for statistical significance using non-parametric ANOVA Kruskal–Wallis (KW) test and Dunn's post-test for multiple comparisons. The *P*-values plotted on the bottom show the KW test results and the *P*-values plotted on the top show the post-test analysis comparing age groups. (**I**) Anti-SARS-CoV-2 spike IgG, RBD IgG, and PSV-neutralizing titers in 25-μg and 100-μg mRNA-1273 vaccinees at day 43 (two weeks post second dose). (**J**) Spike-specific CD4$^+$ AIM$^+$ (left) and cytokine$^+$ (right) T cells in mRNA-1273 vaccinees. (**K**) Spike-specific CD8$^+$ AIM$^+$ (left) and cytokine$^+$ (right) T cells in mRNA-1273 vaccinees. Dotted green lines indicate the limit of quantification (LOQ). The bars indicate geometric mean and geomean SD. Data in (I) to (K) were analyzed for statistical significance using Mann–Whitney *U* test. NS, non-significant. Background-subtracted and log data analyzed in all cases.

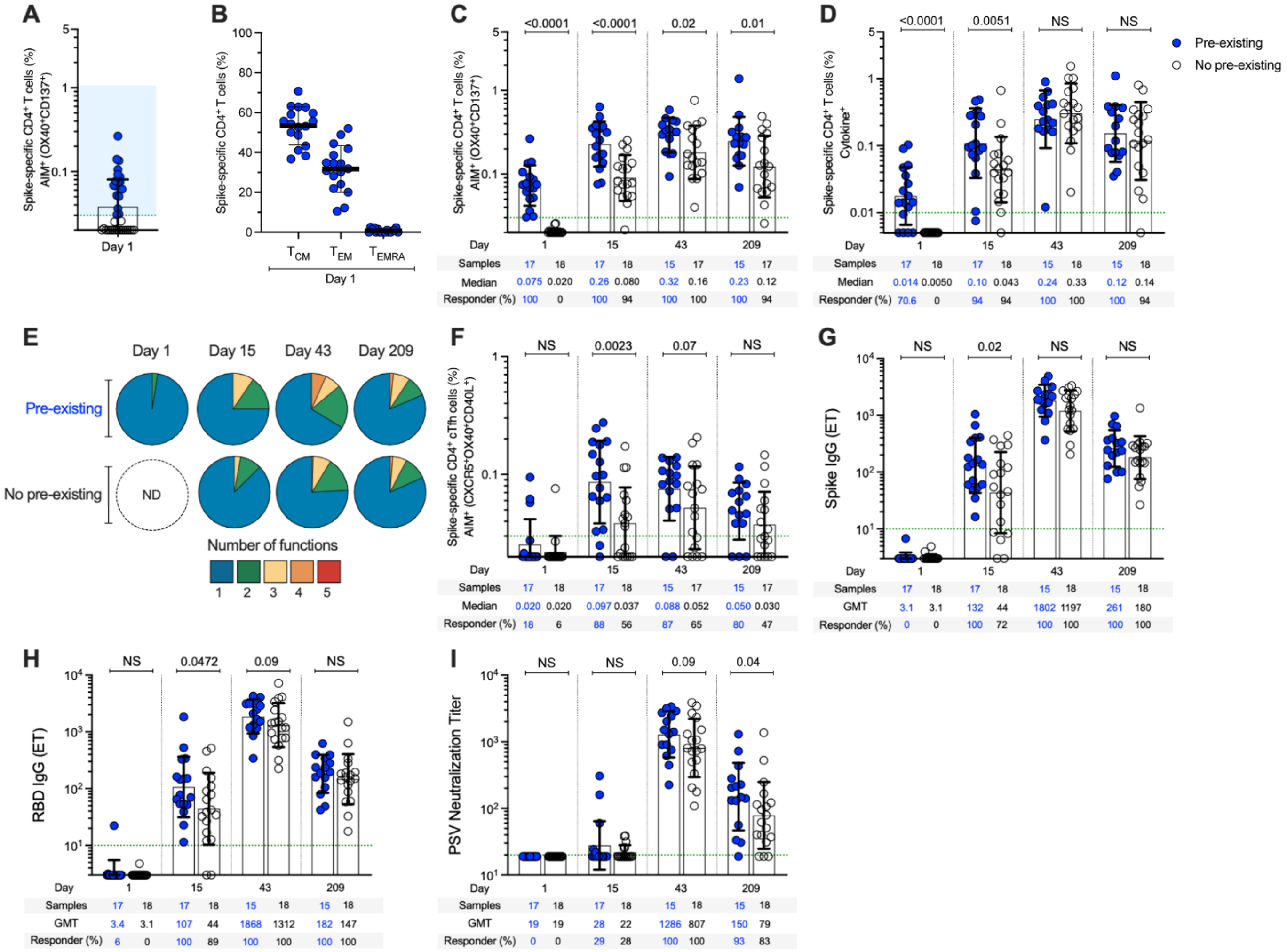

Fig. 5. Pre-existing anti-spike immunity modulates T cell and antibody responses. (**A**) Pre-existing spike-specific CD4+ AIM+ T cells at day 1 (see Fig. 2 for details). (**B**) Memory phenotype of pre-existing spike-specific CD4+ AIM+ T cells from (A). (**C**) Spike-specific CD4+ AIM+ T cells in mRNA-1273 vaccinees with ("pre-existing", blue) and without ("no pre-existing", white) pre-existing cross-reactive spike-reactive memory CD4+ T cells evaluated on days 1, 15 ± 2, 43 ± 2, and 209 ± 7 post-immunization (see fig. S13 for details). (**D**) Spike-specific CD4+ cytokine+ T cells in mRNA-1273 vaccinees with and without pre-existing cross-reactive memory CD4+ T cells. (**E**) Proportions of multifunctional spike-specific CD4+ T cells in mRNA-1273 vaccinees with ("pre-existing") and without ("no pre-existing") pre-existing cross-reactive spike-reactive memory CD4+ T cells evaluated on days 1, 15 ± 2, 43 ± 2, and 209 ± 7 post-immunization (see fig. S10 for details). (**F**) Spike-specific cT_{FH} cells (as percentage of CD4+ T cells); (**G**) Anti-spike IgG; (**H**) Anti-RBD IgG; and (**I**) SARS-CoV-2 PSV-neutralizing titers in mRNA-1273 vaccinees without and with pre-existing cross-reactive spike-reactive memory CD4+ T cells. Dotted green lines indicate the limit of quantification (LOQ). The bars in (A), (C), (D), (E), (F), (G), (H), and (I) indicate the geometric mean and geomean SD in the analysis of the antibody levels or spike-specific CD4+ and CD8+ T cells in mRNA-1273 vaccinees with ("pre-existing") and without ("no pre-existing") pre-existing cross-reactive spike-reactive memory CD4+ T cells evaluated on days 1, 15 ± 2, 43 ± 2, and 209 ± 7 post-immunization. The bars in B indicate the mean and SD in the analysis of the memory phenotype of spike-specific CD4+ T cells. Data were analyzed for statistical significance using Mann–Whitney *U* test. NS, non-significant, ND, non-detectable. Background-subtracted and log data analyzed in all cases.
