# Supplementary material for: Low-dose mRNA-1273 COVID-19 vaccine generates durable memory enhanced by cross-reactive T cells
Source: Science. 2021 Oct 22;374(6566):eabj9853. doi: 10.1126/science.abj9853 (PMC8542617; doi:10.1126/science.abj9853)
Supplement: Supplementary file 3 — MDAR Reproducibility Checklist [file science.abj9853_mdar_reproducibility_checklist.pdf]

Materials Design Analysis Reporting (MDAR) Checklist for Authors

The MDAR framework establishes a minimum set of requirements in transparent reporting applicable to studies in the life sciences (see Statement of Task: doi:10.31222/osf.io/9sm4x.). The MDAR checklist is a tool for authors, editors and others seeking to adopt the MDAR framework for transparent reporting in manuscripts and other outputs. Please refer to the MDAR Elaboration Document for additional context for the MDAR framework.

Materials

**Antibodies**	**Yes (indicate where provided: page no/section/legend)**	**n/a**
For commercial reagents, provide supplier name, catalogue number and RRID, if available.	Supplemental material file:<br>- Table S6.<br>- Table S8	

**Cell materials**	**Yes (indicate where provided: page no/section/legend)**	**n/a**
**Cell lines:** Provide species information, strain. Provide accession number in repository **OR** supplier name, catalog number, clone number, **OR** RRID	Materials and methods section, Pseudovirus (PSV) Neutralization Assay	
**Primary cultures:** Provide species, strain, sex of origin, genetic modification status.		N/A

**Experimental animals**	**Yes (indicate where provided: page no/section/legend)**	**n/a**
**Laboratory animals:** Provide species, strain, sex, age, genetic modification status. Provide accession number in repository **OR** supplier name, catalog number, clone number, **OR** RRID		N/A
**Animal observed in or captured from the field:** Provide species, sex and age where possible		N/A
**Model organisms:** Provide Accession number in repository (where relevant) **OR** RRID		N/A

**Plants and microbes**	**Yes (indicate where provided: page no/section/legend)**	**n/a**
**Plants:** provide species and strain, unique accession number if available, and source (including location for collected wild specimens)		N/A
**Microbes:** provide species and strain, unique accession number if available, and source		N/A

**Human research participants**	**Yes (indicate where provided: page no/section/legend)**	**n/a**
Identify authority granting ethics approval (IRB or equivalent committee(s), provide reference number for approval.	Material and methods section:<br>- Samples from the phase I trial of 25-µg mRNA-1273 SARS-CoV-2	
Provide statement confirming informed consent obtained from study participants.	Material and methods section:<br>- Samples from the phase I trial of 25-µg mRNA-1273 SARS-CoV-2<br>- Samples from convalescent COVID-19 donors<br>- 100-µg mRNA-1273 vaccinees	
Report on age and sex for all study participants.	Material and methods section:<br>- Samples from the phase I trial of 25-µg mRNA-1273 SARS-CoV-2<br>- Samples from convalescent COVID-19 donors<br>- 100-µg mRNA-1273 vaccinees<br>- Table S3<br>- Table S4<br>- Table S5<br>- Table S7	

Design

Study protocol	Yes (indicate where provided: page no/section/legend)	n/a
For clinical trials, provide the trial registration number **OR** cite DOI in manuscript.	Material and methods section	

Laboratory protocol	Yes (indicate where provided: page no/section/legend)	n/a
Provide DOI or other citation details if detailed step-by-step protocols are available.	The Materials and methods section includes the detailed description of each protocol used in the study. Further, references supporting the protocols were included.	

Experimental study design (statistics details)	Yes (indicate where provided: page no/section/legend)	n/a
State whether and how the following have been done, **or** if they were not carried out.		
Sample size determination		N/A
Randomisation		N/A
Blinding		N/A
Inclusion/exclusion criteria		N/A

Sample definition and in-laboratory replication	Yes (indicate where provided: page no/section/legend)	n/a
State number of times the experiment was replicated in laboratory	The replicates for each in vitro experiment and the numbers of samples used in each experiment are described in the Materials and methods section	
Define whether data describe technical or biological replicates	The number of individuals (either 25-µg vaccinees, 100-µg vaccinees, and COVID-19 convalescent donors) included in each experiment is described in each figure, figure legend and the Materials and methods section. Also, details of each cohort are provided in Tables S3, S4, S5 and S7.	

Ethics	Yes (indicate where provided: page no/section/legend)	n/a
Studies involving human participants: State details of authority granting ethics approval (IRB or equivalent committee(s), provide reference number for approval.	Material and methods section:<br>- Samples from the phase I trial of 25-µg mRNA-1273 SARS-CoV-2	
Studies involving experimental animals: State details of authority granting ethics approval (IRB or equivalent committee(s), provide reference number for approval.		N/A
Studies involving specimen and field samples: State if relevant permits obtained, provide details of authority approving study; if none were required, explain why.		N/A

Dual Use Research of Concern (DURC)	Yes (indicate where provided: page no/section/legend)	n/a
If study is subject to dual use research of concern, state the authority granting approval and reference number for the regulatory approval		N/A

Analysis

Attrition	Yes (indicate where provided: page no/section/legend)	n/a
State if sample or data point from the analysis is excluded, and whether the criteria for exclusion were determined and specified in advance.	Supplementary material file, Table S3	

Statistics	Yes (indicate where provided: page no/section/legend)	n/a
Describe statistical tests used and justify choice of tests.	Materials and methods section<br>Each figure legend also described the statistics used in the data analysis in the figure panels.	

Data Availability	Yes (indicate where provided: page no/section/legend)	n/a
State whether newly created datasets are available, including protocols for access or restriction on access.	Experimental data are provided in the supplemental data.<br>Protocols are described in Material and methods section.	
If data are publicly available, provide accession number in repository or DOI or URL.		N/A
If publicly available data are reused, provide accession number in repository or DOI or URL, where possible.		N/A

Code Availability	Yes (indicate where provided: page no/section/legend)	n/a
For all newly generated code and software essential for replicating the main findings of the study:		
State whether the code or software is available.		N/A
If code is publicly available, provide accession number in repository, or DOI or URL.		N/A

Reporting

Adherence to community standards	Yes (indicate where provided: page no/section/legend)	n/a
MDAR framework recommends adoption of discipline-specific guidelines, established and endorsed through community initiatives. Journals have their own policy about requiring specific guidelines and recommendations to complement MDAR.		
State if relevant guidelines (eg., ICMJE, MIBBI, ARRIVE) have been followed, and whether a checklist (eg., CONSORT, PRISMA, ARRIVE) is provided with the manuscript.		N/A
