# Supplementary material for: Low-dose mRNA-1273 COVID-19 vaccine generates durable memory enhanced by cross-reactive T cells
Source: Science. 2021 Oct 22;374(6566):eabj9853. doi: 10.1126/science.abj9853 (PMC8542617; doi:10.1126/science.abj9853)
Supplement: Supplementary file 4 — Data S1 [file science.abj9853_data_s1.zip › science.abj9853_data_s1_caption.docx]

**Data S1.** Spike-specific antibody and T cell data in low-dose vaccines (.xlsx file).
