# Supplementary material for: Low-dose mRNA-1273 COVID-19 vaccine generates durable memory enhanced by cross-reactive T cells
Source: Science. Author manuscript; Available in PMC 2021 Oct 25. (PMC8542617; doi:10.1126/science.abj9853)
Supplement: Supplementary file 1 [file NIHMS1743942-supplement-supplementary_material.pdf]

Science
AAAS

Supplementary Materials for

Low-dose mRNA-1273 COVID-19 vaccine generates durable memory enhanced by cross-reactive T cells

Jose Mateus *et al.*

Corresponding authors: Alessandro Sette, alex@lji.org; Shane Crotty, shane@lji.org; Daniela Weiskopf, daniela@lji.org

DOI: 10.1126/science.abj9853

The PDF file includes:

Figs. S1 to S13
Tables S1 to S8
References

Other Supplementary Material for this manuscript includes the following:

MDAR Reproducibility Checklist
Data S1

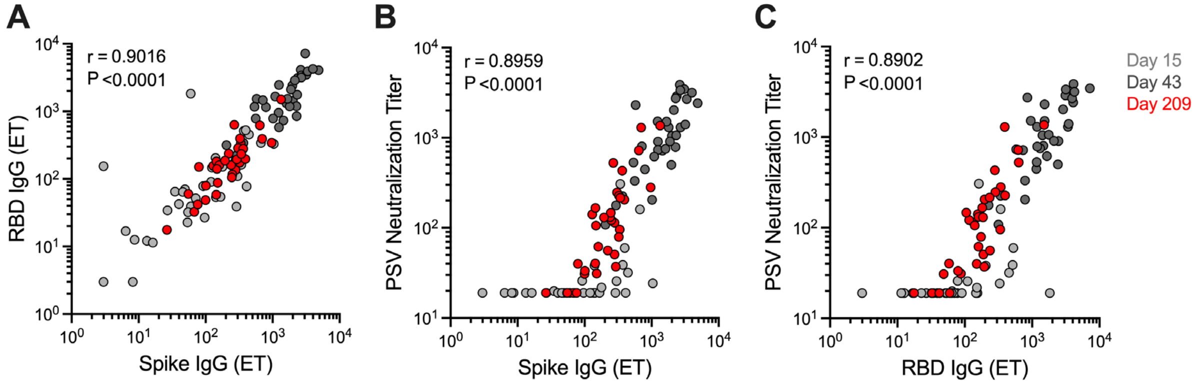

Fig. S1. Correlation of antibody responses in mRNA-1273 vaccinees.
(A) Correlation of anti-spike IgG and RBD IgG titers in mRNA-1273 vaccinees on days 15±2, 43±2, and 209±7. **(B)** Correlation of anti-spike IgG and PSV neutralization titers in mRNA-1273 vaccinees on days 15±2, 43±2, and 209±7. **(C)** Correlation of anti-RBD IgG and PSV neutralization titers in mRNA-1273 vaccinees on days 15±2, 43±2, and 209±7. Background-subtracted and log data analyzed in all cases. Day 15±2 = light gray, day 43±2 = dark gray, day 209±7 = red. Data were analyzed for statistical significance using Spearman's rank correlation test.

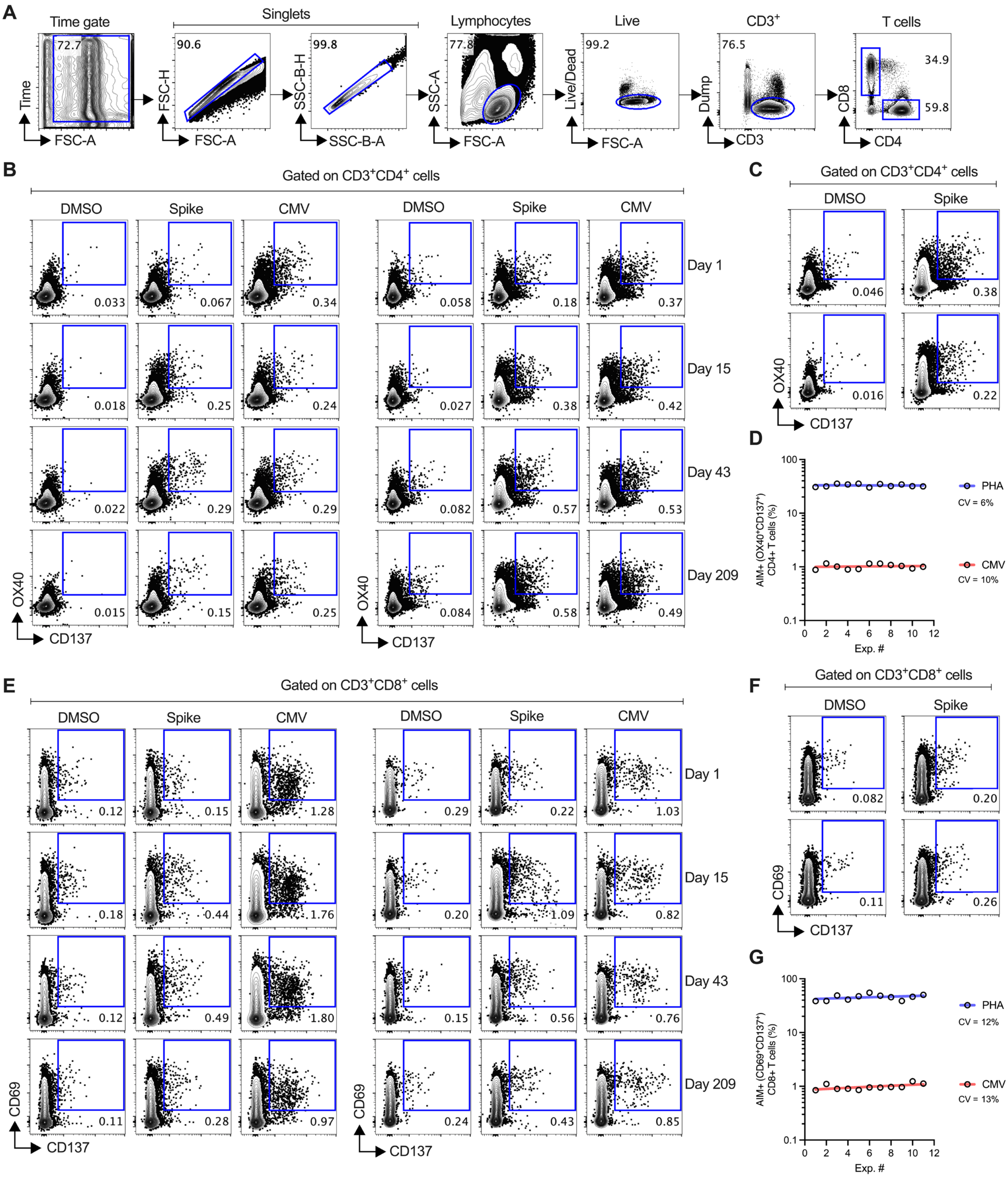
A
Time gate
Singlets
Lymphocytes
Live
CD3+
T cells
B
Gated on CD3+CD4+ cells
DMSO
Spike
CMV
Day 1
Day 15
Day 43
Day 209
OX40
CD137
C
D
PHA
CV = 6%
CMV
CV = 10%
Exp. #
E
Gated on CD3+CD8+ cells
CD69
F
G
CV = 12%
CV = 13%

Fig. S2. Assessment of spike-specific T cells using an Activation Induced Marker (AIM) assay.
(A) Representative strategies to define $CD3^+CD4^+$ and $CD3^+CD8^+$ cells by AIM assay. **(B and C)** Representative flow cytometry plots of spike-specific $CD4^+$ T cells ($OX40^+CD137^+$, after overnight stimulation with spike megapool (MP) or CMV MP), compared to negative control (DMSO). Representative examples from two mRNA-1273 vaccinees (B) and two COVID-19 convalescent subjects (C). **(D)** CMV-specific $CD4^+$ T cell responses evaluated for a SARS-CoV-2-unexposed donor in the AIM experiments. This sample was included as internal quality control to define inter-assay variation. The antigen-specific response against CMV (red line) and the response to the positive control (PHA; blue line) was compared across the eleven independent experiments and revealed a coefficient of variation (CV) of 6% for mitogenic stimulation with PHA and a CV of 10% for the antigen-specific stimulation with the CMV MP. **(E and F)** Representative flow cytometry plots of spike-specific $CD8^+$ T cells ($CD69^+CD137^+$, after overnight stimulation with spike MP or CMV MP), compared to DMSO. Representative examples from two mRNA-1273 vaccinees (E) and two COVID-19 convalescent subjects (F). **(G)** CMV-specific $CD8^+$ T cell responses evaluated for a SARS-CoV-2-unexposed donor in the AIM experiments. This sample was included as internal quality control to define inter-assay variation. Similar to the strategy used for the AIM^+ $CD4^+$ T cells, the antigen-specific response against CMV (red line) and the response to the positive control (PHA; blue line) were compared across the eleven independent experiments and revealed a CV of 12% for mitogenic stimulation with PHA and a CV of 13% for the antigen-specific stimulation with the CMV MP. Background-subtracted and log data analyzed in D and G.

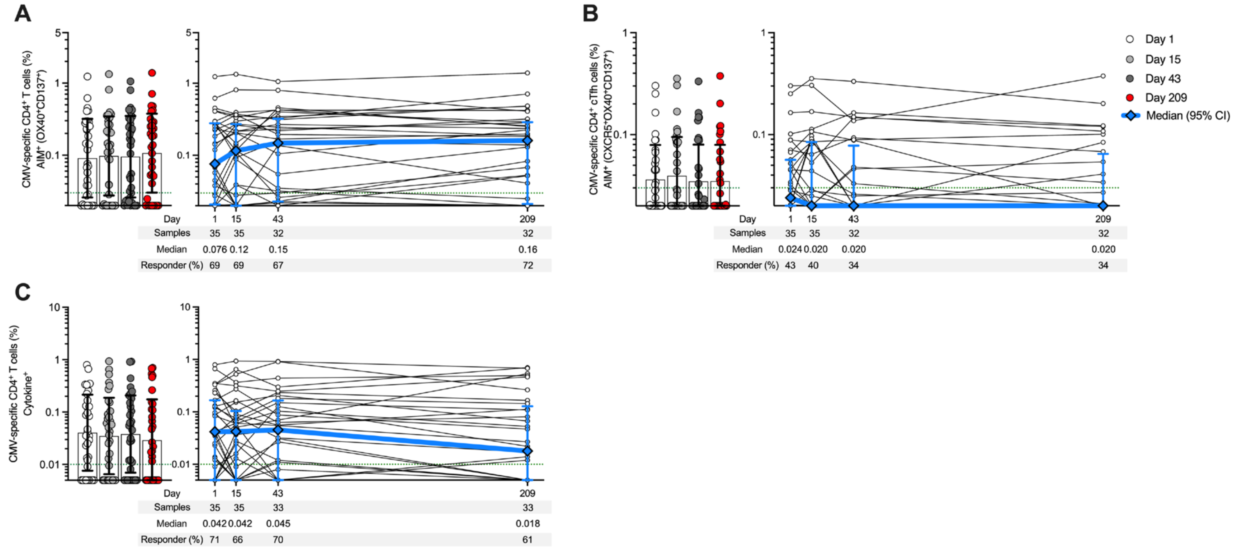

Fig. S3. CMV-specific CD4$^+$ T cell responses in mRNA-1273 vaccinees.

(A) Longitudinal CMV-specific CD4$^+$ AIM$^+$ T cells in mRNA-1273 vaccinees. Percentage of background subtracted CMV-specific CD4$^+$ T cells quantified by AIM (OX40$^+$CD137$^+$) after stimulation with CMV MP in mRNA-1273 vaccinees. **(B)** Quantitation of CMV-specific circulating T follicular helper (cT$_{FH}$) cells (CXCR5$^+$OX40$^+$sCD40L$^+$, as percentage of CD4$^+$ T cells) after overnight stimulation with CMV MP. **(C)** Longitudinal CMV-specific CD4$^+$ cytokine$^+$ T cells expressing iCD40L or producing IFNγ, TNFα, IL-2 or GzB in mRNA-1273 vaccinees. Percentage of background subtracted CMV-specific CD4$^+$ T cells quantified by ICS after stimulation with the CMV MP in mRNA-1273 vaccinees. A Boolean gating strategy was used to define the frequencies of CD4$^+$ T cells producing either expressing iCD40L or producing IFNγ, TNFα, IL-2, or GzB. The dotted green line indicates limit of quantification (LOQ). The bars in A, B, and C indicate the geometric mean and geometric SD in the analysis of the CMV-specific CD4$^+$ T cell frequencies on days 1, 15±2, 43±2, and 209±7 postimmunization. The bottom panels in A, B, and C show the samples included in each group, the median of the frequencies, and the percentage of responders. Day 1 = white, day 15±2 = light gray, day 43±2 = dark gray, day 209 7 = red. Data were analyzed for statistical significance using Spearman's rank correlation test. Background-subtracted and log data analyzed in all cases.

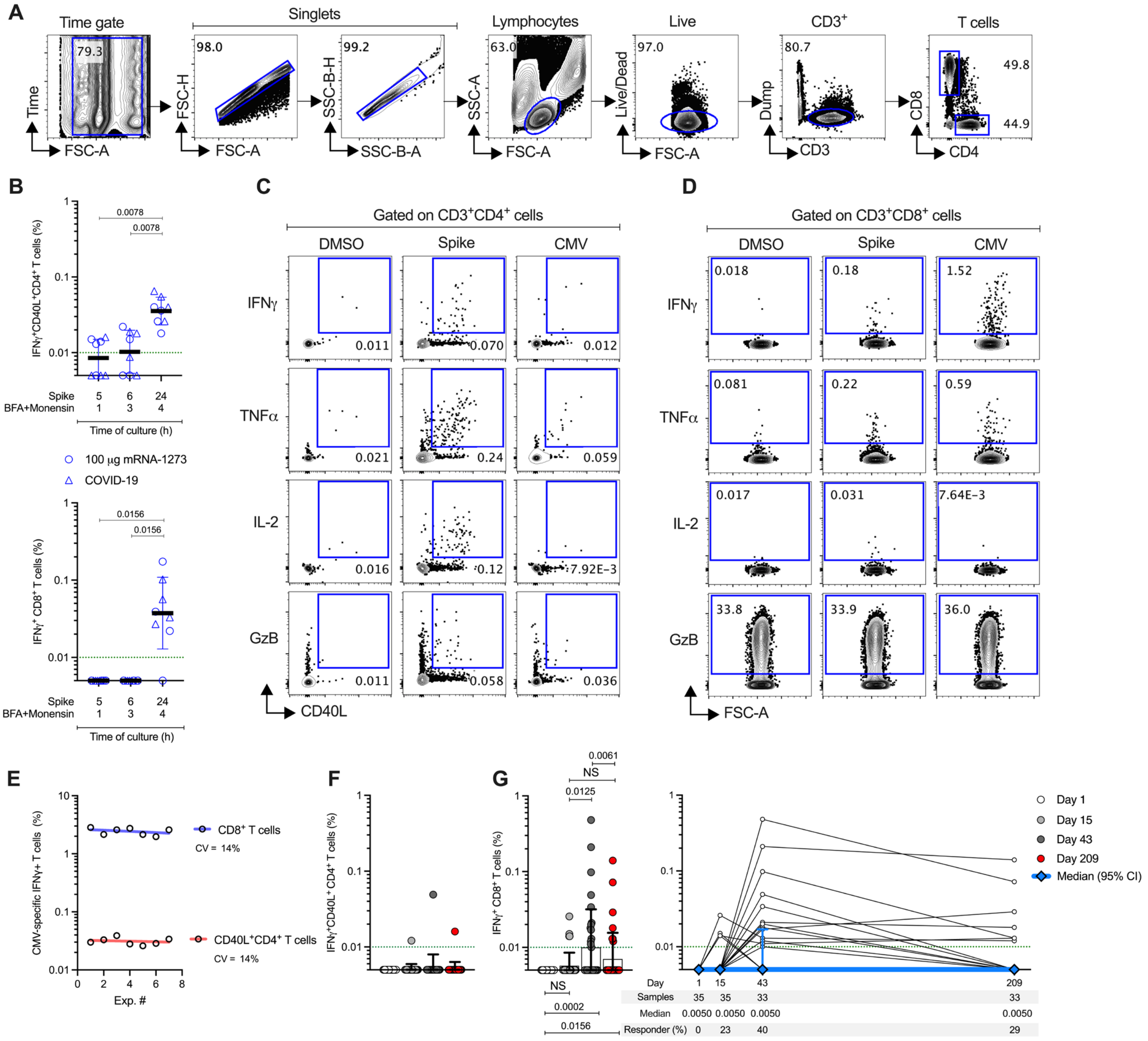

Fig. S4. Assessment of spike-specific T cells using an Intracellular Cytokine (ICS) assay.
(A) Representative strategies to define CD3$^+$CD4$^+$ and CD3$^+$CD8$^+$ cells by the ICS assay. **(B)** Spike-specific CD4$^+$ and CD8$^+$ T cells producing IFNγ at 5, 6, and 24 hours of stimulation with spike MP. To optimize spike-specific detection of cytokine-producing CD4$^+$ and CD8$^+$ T cells, we experimented with different incubation times (5, 6, and 24 hours) with the addition of brefeldin A (BFA) and monensin (1, 3, and 4 hours, respectively). We evaluated the IFNγ-producing CD4$^+$ and CD8$^+$ T cells in 100-µg mRNA-1273 vaccinees (n=4) and COVID-19 convalescent donors (n=4) (See **table S7** for details). The highest signal of IFNγ-producing T cells was detected after 24+4 hours for both spike-specific CD4$^+$ and CD8$^+$ T cells (upper and lower panel, respectively) in vaccinees and convalescent donors. Thus, we chose 24 hours as the best condition to identify spike-specific CD4$^+$ and CD8$^+$ T cells producing cytokines. **(C-D)** Representative flow cytometry plots of spike-specific CD4$^+$CD40L$^+$ or CD8$^+$ T cells producing IFNγ, TNFα, IL-2, and GzB after overnight stimulation with spike MP or CMV MP, compared to the negative control (DMSO). Representative examples from one mRNA-1273 vaccinees at day 43±2. **(E)** CMV-specific CD4$^+$CD40L$^+$ and CD8$^+$ T cell responses evaluated for a SARS-CoV-2-unexposed donor in the ICS experiments. This sample was included as internal quality control to define the variation inter-assay. The CMV-specific CD40L$^+$CD4$^+$ and CD8$^+$ T cell response measured by IFNγ-production was compared across the seven independent experiments and revealed a coefficient of variation (CV) of 14% for CD4$^+$ and CD8$^+$ T cells. **(F-G)** Spike-specific CD8$^+$ T cell responses with an optimal pool of Class-I epitopes (CD8 spike MP) in mRNA-1273 vaccinees. To confirm that the spike-specific CD8$^+$ T cell response

observed in mRNA-1273 vaccinees is also induced in the absence of the spike-specific $CD4^+$ T cell response, we incubated PBMCs with the CD8 spike MP to evaluate the IFNγ-producing T cells, as described above. As excepted, CD8 spike MP does not induce $CD4^+$ T cell response at any timepoint postimmunization in the 25-μg mRNA-1273 vaccinees (F). Only in 1/35 (0.35%) donor was detected the $CD4^+$ T cell response using the CD8 spike MP (CD8-S MP), consisting of 9 and 10-mers, Class-I restricted epitopes. (G) Spike-specific $CD8^+$ T cell response using the optimal MP was detectable in 23% (8/35) of the subjects after the first immunization. The spike-specific $CD8^+$ T cell response was significantly increased after the second immunization on days 43 and 209 and detected in 40% (14/35) and 29% (10/35) of the subjects, respectively. This response follows similar kinetics of what had been seen in spike-specific $CD8^+$ T cells producing IFNγ in response to the overlapping spike MP consisting of overlapping 15-mers (Fig. 3). The dotted green line indicates limit of quantification (LOQ). The bars in B, F, and G indicate the geometric mean and geometric SD in the analysis of the spike-specific $CD4^+$ and $CD8^+$ T cell frequencies on days 1, 15±2, 43±2, and 209±7 postimmunization. The bottom panel in G shows the samples included in each group, the median of the frequencies, and the percentage of responders. Day 1 = white, day 15±2 = light gray, day 43±2 = dark gray, day 209±7 = red. Data were analyzed for statistical significance using (B) Mann–Whitney *U* test and (G) Wilcoxon signed-rank test. Background-subtracted and log data analyzed in all cases. Background-subtracted and log data analyzed in B, E, F, and G.

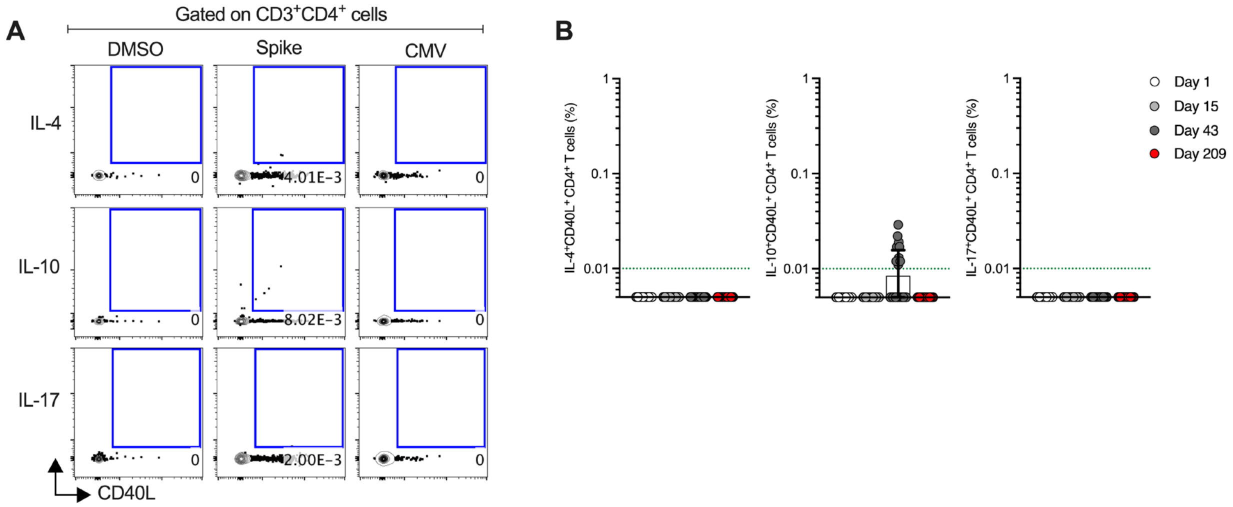

Fig. S5. Spike-specific CD4$^+$ T cells producing IL-4, IL-10, and IL-17 in mRNA-1273 vaccinees.
(A) Representative examples of flow cytometry plots of spike-specific CD4$^+$CD40L$^+$ producing IL-4, IL-10, and IL-17 after overnight stimulation with spike MP or CMV MP, compared to the negative control (DMSO). Representative examples from one mRNA-1273 vaccinees at day 43 ± 2. **(B)** Longitudinal spike-specific CD4$^+$CD40L$^+$ T cells producing IL-4, IL-10, and IL-17 in mRNA-1273 vaccinees. No IL-4- or IL-17-producing CD4$^+$ T cells in response to spike MP were detected at any timepoint postimmunization in 25-µg mRNA-1273 vaccinees. Spike-specific IL-10-producing CD4$^+$ T cell response was detected in 36% (12/33) of the subjects on day 43 but was undetectable in other timepoints. The dotted green line indicates limit of quantification (LOQ). The bars in B indicate the geometric mean and geometric SD in the analysis of the spike-specific CD4$^+$ T cell frequencies on days 1, 15±2, 43 2, and 209±7 postimmunization. Day 1 = white, day 15±2 = light gray, day 43±2 = dark gray, day 209±7 = red. Background-subtracted and log data analyzed in all cases. Background-subtracted and log data analyzed in all cases.

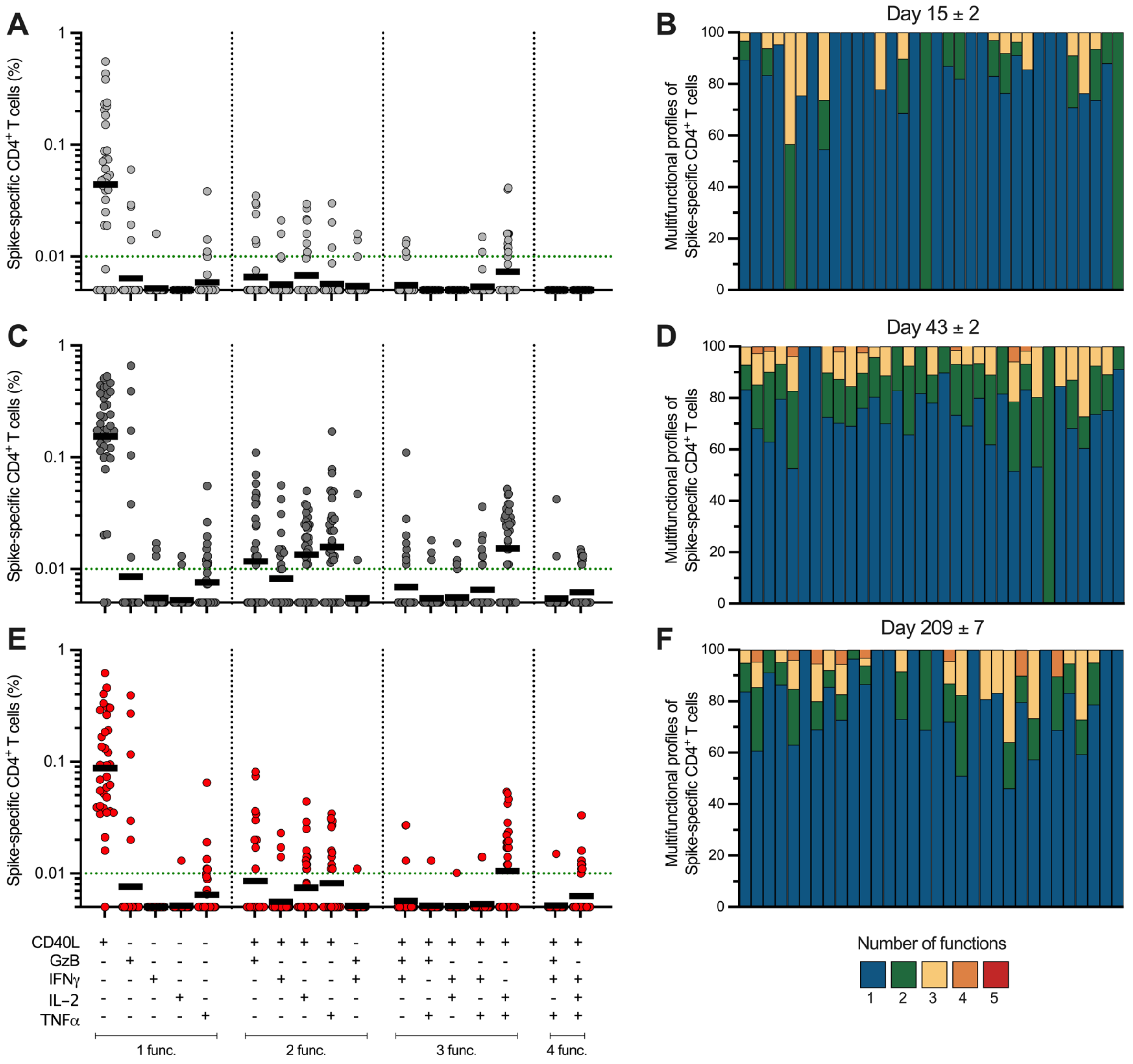

Fig. S6. Multifunctional profiles of spike-specific CD4$^+$ T cells in mRNA-1273 vaccinees.
(A, C, E) Predominant multifunctional profiles of spike-specific CD4$^+$ T cells with one, two, three, and four functions were analyzed in mRNA-1273 vaccinees on days 15±2 (A), 43±2 (C), and 209±7 (E) postimmunization. A Boolean analysis was carried out to define the multifunctional profiles on FlowJo 10.7.1 and the analysis included CD40L, GzB, IFNγ, IL-2, and TNFα gated on CD3$^+$CD4$^+$ cells (See **fig. S4**). The overall response to spike was defined as the sum of the background subtracted responses to each combination of individual cytokines. Responses >0.005% and a SI>2 for CD4$^+$ T cells was considered positive. Functional profiles detected in at least one individual are shown in A, C, and E. The dotted green line indicates limit of quantification (LOQ). The bars show the geometric mean and geometric SD of the spike-specific CD4$^+$ T cells. **(B, D, F)** Proportion of multifunctional spike-specific CD4$^+$ T cells with one, two, three, and four functions in mRNA-1273 vaccinees on days 15±2 (B), 43±2 (D), and 209±7 (F) postimmunization. Each bar shows the proportion of multifunctional Ag-specific CD4$^+$ T cells detected per donor. The blue, green, yellow, and orange colors in the pie charts depict the production of one, two, three, and four functions, respectively. Data showed in B, D, and F were used to calculate spike-specific CD4$^+$ T cells producing one, two, three, and four functions on days 15±2 (B), 43±2 (D), and 209±7 (F) postimmunization. Values are shown in **table S1** and plot in **Fig. 2F**. Background-subtracted and log data analyzed in all cases.

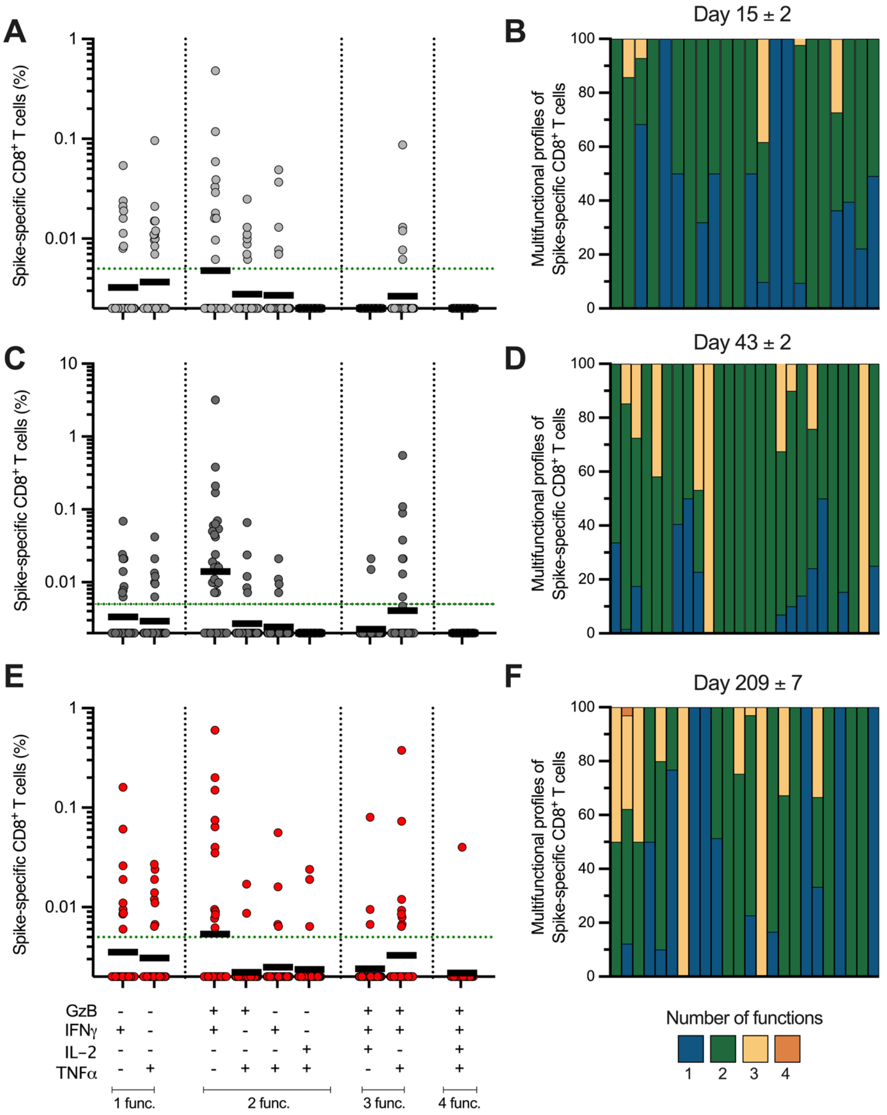

Fig. S7. Multifunctional profiles of spike-specific CD8$^+$ T cells in mRNA-1273 vaccinees.
(A, C, E) Predominant multifunctional profiles of spike-specific CD8$^+$ T cells with one, two, three, and four functions were analyzed in mRNA-1273 vaccinees on days 15±2 (A), 43±2 (C), and 209±7 (E) postimmunization. A Boolean analysis was carried out to define the multifunctional profiles on FlowJo 10.7.1 and the analysis included GzB, IFNγ, IL-2, and TNFα gated on CD3$^+$CD8$^+$ cells (See **fig. S4**). The overall response to spike was defined as the sum of the background subtracted responses to each combination of individual cytokines. Responses >0.005% and a SI>2 for CD8$^+$ T cells was considered positive. Functional profiles detected in at least one individual are shown in A, C, and E. The dotted green line indicates limit of quantification (LOQ). The bars show the geometric mean and geometric SD of the spike-specific CD8$^+$ T cells. **(B, D, F)** Proportion of multifunctional spike-specific CD8$^+$ T cells with one, two, three, and four functions in mRNA-1273 vaccinees on days 15±2 (B), 43±2 (D), and 209±7 (F) postimmunization. Each bar shows the proportion of multifunctional Ag-specific CD8$^+$ T cells detected per donor. The blue, green, yellow, and orange colors in the pie charts depict the production of one, two, three, and four functions, respectively. Data showed in B, D, and F were used to calculate spike-specific CD8$^+$ T cells producing one, two, three, and four functions on days 15±2 (B), 43±2 (D), and 209±7 (F) postimmunization. Values are shown in **table S2** and plot in **Fig. 3E**. Background-subtracted and log data analyzed in all cases.

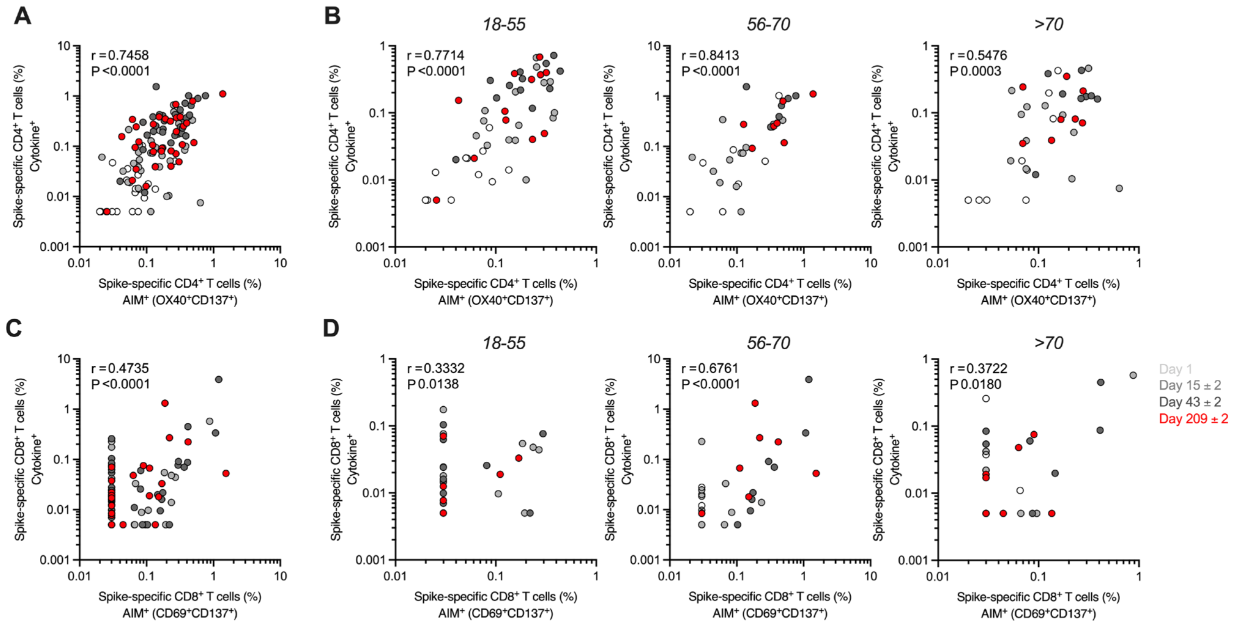

Fig. S8. Correlation of spike-specific CD4$^+$ and CD8$^+$ T cell response detected by AIM and ICS assays in mRNA-1273 vaccinees.

(A) Correlation of spike-specific CD4$^+$ T cell response using AIM and ICS assays in mRNA-1273 vaccinees. **(B)** Correlation of spike-specific CD4$^+$ T cell response using AIM and ICS assays in mRNA-1273 vaccinees by age groups. AIM$^+$CD4$^+$ T cell response was defined based on the double-expression of OX40$^+$CD137$^+$ (See **Fig. S2**) and ICS$^+$CD4$^+$ T cell response was defined based on the expression of iCD40L and the production of IFNγ, TNFα, IL-2, and GzB (See **Fig. S4**). **(C)** Correlation of spike-specific CD8$^+$ T cell response using AIM and ICS assays in mRNA-1273 vaccinees. **(D)** Correlation of spike-specific CD8$^+$ T cell response using AIM and ICS assays in mRNA-1273 vaccinees by age groups. AIM$^+$CD8$^+$ T cell response was defined based on the double-expression of CD69$^+$CD137$^+$ (See **Fig. S2**) and ICS$^+$CD8$^+$ T cell response was defined based on the production of IFNγ, TNFα, IL-2 or GzB (See **Fig. S4**). The response was analyzed on PBMCs after an overnight stimulation with overlapping spike MP as described in the Materials and Methods section. Background-subtracted and log data analyzed in all cases. Day 1 = white, day 15 ± 2 = light gray, day 43 ± 2 = dark gray, day 209 ± 7 = red. Data were analyzed for statistical significance using Spearman's rank correlation test.

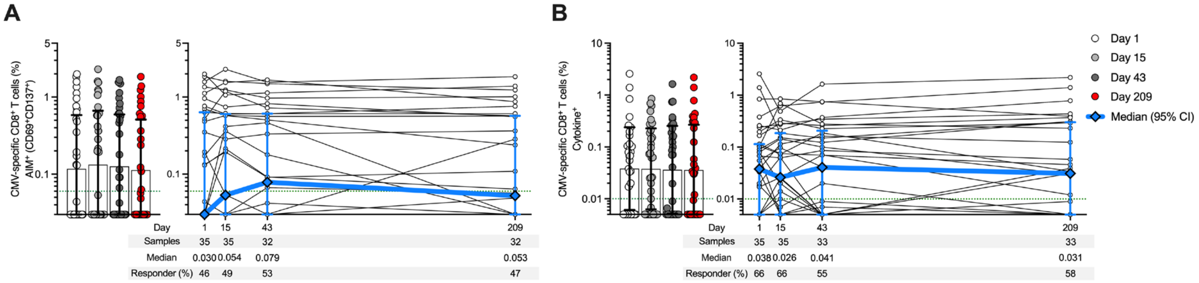

Fig. S9. CMV-specific CD8⁺ T cell responses in mRNA-1273 vaccinees.

(A) Longitudinal CMV-specific $CD8^+$ AIM^+ T cells in mRNA-1273 vaccinees. Percentage of background subtracted CMV-specific $CD8^+$ T cells quantified by AIM ($CD69^+CD137^+$) after stimulation with CMV MP in mRNA-1273 vaccinees (See **fig. S2**). **(B)** Longitudinal CMV-specific $CD8^+$ $cytokine^+$ T cells producing IFNγ, TNFα, IL-2 or GzB in mRNA-1273 vaccinees. Percentage of background subtracted CMV-specific $CD8^+$ T cells quantified by ICS after stimulation with the CMV MP in mRNA-1273 vaccinees (See **fig. S4**). A Boolean gating strategy was used to define the frequencies of $CD8^+$ T cells producing IFNγ, TNFα, IL-2 or GzB. The dotted green line indicates limit of quantification (LOQ). The bars in A and B indicate the geometric mean and geometric SD in the analysis of the CMV-specific $CD8^+$ T cell frequencies on days 1, 15±2, 43±2, and 209±7 postimmunization. The bottom panels in A and B show the samples included in each group, the median of the frequencies, and the percentage of responders. Day 1 = white, day 15±2 = light gray, day 43±2 = dark gray, day 209±7 = red. Background-subtracted and log data analyzed in all cases.

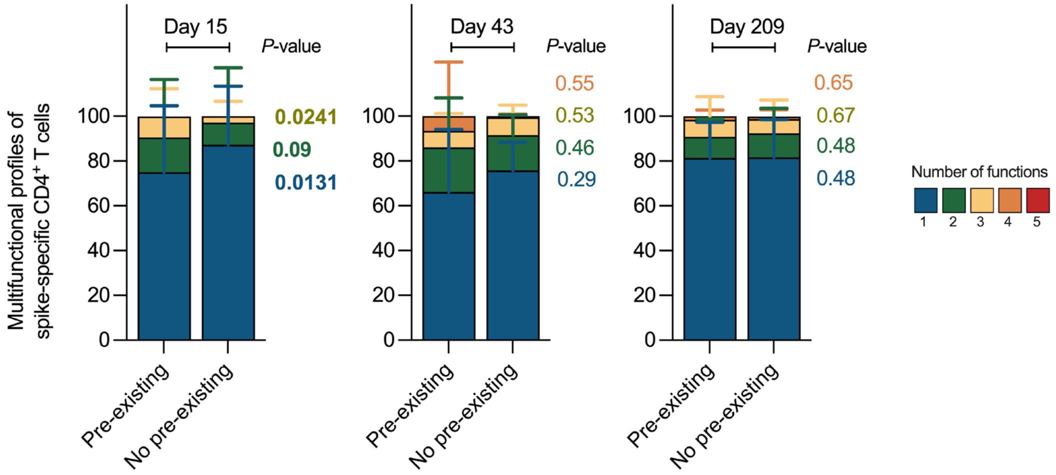

Fig. S10. Multifunctional spike-specific CD4$^+$ T cells in individuals with and without pre-existing spike immunity.

Comparison of multifunctional profiles of spike-specific CD4$^+$ T cells in individuals with and without pre-existing spike immunity on days 15±2, 43±2, and 209±7 postimmunization. The blue, green, yellow, and orange colors in the stacked bar charts depict the production of one, two, three, and four functions, respectively. Data were analyzed for statistical significance using the Mann–Whitney *U* test. *P*-values coded by colors shown the comparison between individuals with and without pre-existing spike immunity based on the number of functions of spike-specific CD4$^+$ T cells.

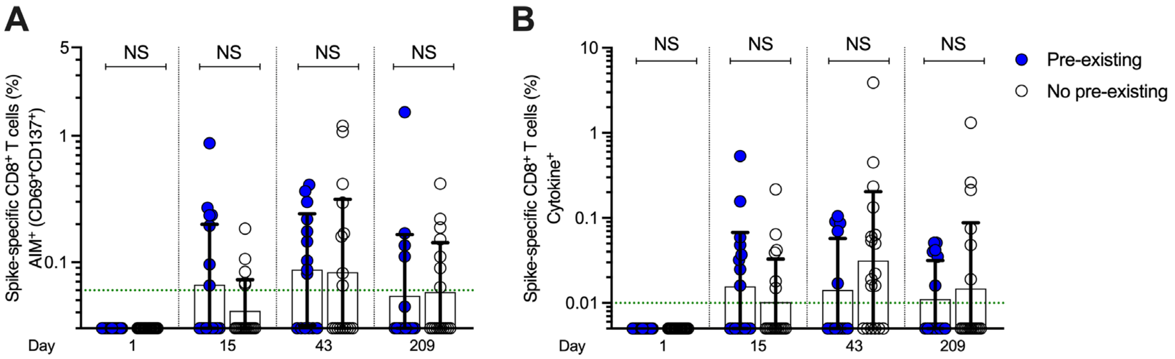

Fig. S11. Spike-specific CD8$^+$ T cell response is not modulated by the pre-existing spike immunity.
(A) Spike-specific CD8$^+$ AIM$^+$ T cells in mRNA-1273 vaccinees with and without pre-existing spike immunity evaluated on days 1, 15±2, 43±2, and 209±7 postimmunization. **(B)** Spike-specific CD8$^+$ cytokine$^+$ T cells in mRNA-1273 vaccinees with and without pre-existing spike immunity evaluated on days 1, 15±2, 43±2, and 209±7 postimmunization. The dotted green line indicates limit of quantification (LOQ). The bars in A and B indicate the geometric mean and geometric SD of the spike-specific CD8$^+$ T cells on days 1, 15±2, 43±2, and 209±7 postimmunization. Vaccinees with pre-existing spike immunity = blue, vaccinees without pre-existing spike immunity = clear. Data were analyzed for statistical significance using Mann–Whitney *U* test. NS, non-significant. Background-subtracted and log data analyzed in all cases.

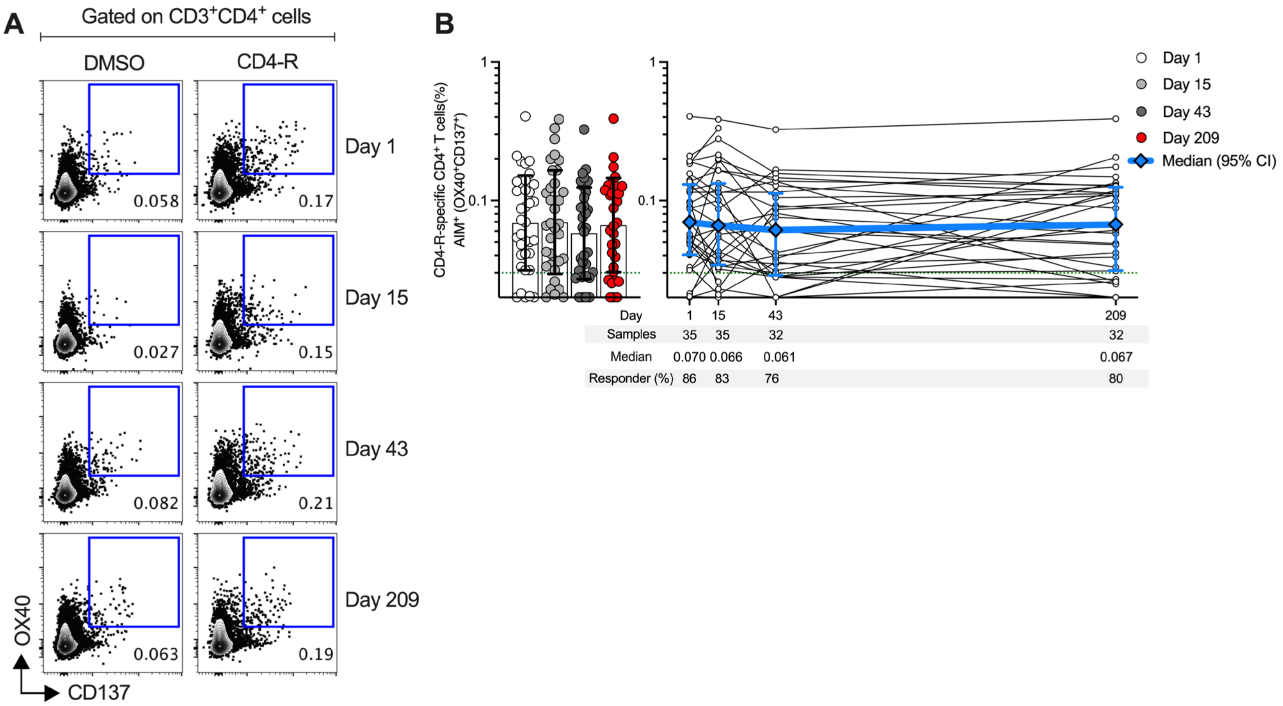

Fig. S12. Non-spike-specific CD4⁺ T cells in mRNA-1273 vaccinees.

(A) Representative flow cytometry plots of CD4-R-specific CD4⁺ T cells (OX40⁺CD137⁺, after overnight stimulation with CD4-R megapool (MP), compared to negative control (DMSO). Representative examples from one mRNA-1273 vaccinee. **(B)** Longitudinal CD4-R-specific CD4⁺ AIM⁺ T cells in mRNA-1273 vaccinees. The green line indicates limit of quantification (LOQ). The bar in B indicates the geometric mean and geometric SD in the analysis of the CD4-R-specific CD4⁺ T cells on days 1, 15 ± 2, 43 ± 2, and 209 ± 7 postimmunization. The bottom panel in B shows the samples included in each group, the median of the frequencies, and the percentage of responders. Day 1 = white, day 15±2 = light gray, day 43±2 = dark gray, day 209±7 = red. Background-subtracted and log data analyzed in all cases.

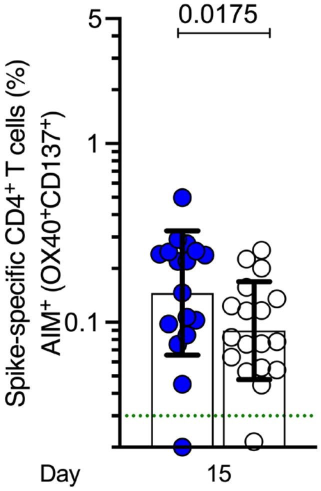

Fig. S13. Spike-specific CD4$^+$ AIM$^+$ T cells in mRNA-1273 vaccinees are higher in individuals with pre-existing spike immunity at day 15±2 post-immunization.

Pre-existing immunity levels detected at day 1 were subtracted from the spike-specific CD4$^+$ AIM$^+$ T cells detected in mRNA-1273 vaccinees at day 15 in each donor. The vaccine response observed in the group with pre-existing immunity is still significantly higher that the group without pre-existing CD4$^+$ T cells (P=0.018). This demonstrates that the spike-specific CD4$^+$ AIM$^+$ T cells in mRNA-1273 vaccinees is enhanced in individuals with pre-existing spike immunity at day 15 post-immunization. The dotted green line indicates the limit of quantification (LOQ). The bars indicate the geometric mean and geometric SD in the analysis of the spike-specific CD4$^+$ T cells on day 15±2 postimmunization. Vaccinees with pre-existing spike immunity = blue, vaccinees without pre-existing spike immunity = clear. Data were analyzed for statistical significance using a Mann–Whitney U test. Background-subtracted and log data analyzed in all cases.

Table S1. Multifunctional spike-specific $CD4^+$ with one, two, three, and four functions detected in mRNA-1273 vaccinees on days 15±2, 43±2, and 209±7[a].

Functions	Day 15±2[b] (Mean±SD)	Day 43±2[b] (Mean±SD)	Day 209±7[b] (Mean±SD)
One	81.2±28.2	71.1±21.6	81.6±16.1
Two	12.7±25	17.7±16.6	10±9.59
Three	6.12±10.3	7.69±6.55	7±9.38
Four	0	3.48±16.8	1.39±2.85
Five	0	0	0

[a]Data plotted in Fig. 2F.
[b]Values calculated from fig. S6B, D, and F.

Table S2. Multifunctional spike-specific CD8$^+$ with one, two, three, and four functions detected in mRNA-1273 vaccinees on days 15±2, 43±2, and 209±7[a].

Functions	Day 15±2[b] (Mean±SD)	Day 43±2[b] (Mean±SD)	Day 209±7[b] (Mean±SD)
One	32.58±35.06	11.55±16.17	32.21±40.8
Two	63.36±35.09	73.73±29.99	48.97±38.46
Three	4.05±10.01	14.72±28.28	18.69±30.24
Four	0	0	0.127±0.6223

[a]Data plotted in Fig. 3E.
[b]Values calculated from fig. S8B, D, and F.

Table S3. Characteristics of the 25-μg mRNA-1273 vaccinees.

Characteristic	25-μg mRNA-1273 cohort	Age groups		
		18-55[a]	56-70[a]	>70[a]
Donors, n	35[c]	15	10	10
Gender, n (%)				
Male	20 (57%)	9 (60%)	3 (30%)	8 (80%)
Female	15 (43%)	6 (40%)	7 (70%)	2 (20%)
Age, years (mean ± SD)	58.4±19.1	36.7±7.9	65.8±4.5	72.8±1.2
Race or ethnicity, n (%)[b]				
Asian	1	0	0	1 (10)
White	34	15 (100)	10 (100)	9 (90)
Hispanic or latino	2	1 (7)	0	1 (10)

[a]Jackson, LA; et al., and Anderson, EJ; et al. reported the characteristics of the participants previously (*9, 28*).
[b]Race or ethnic group could include more than one category per donor.
[c]Two donors who did not receive the second dose were excluded from the analysis of the immune responses on days 43 and 209.

Table S4. Characteristics of COVID-19 convalescent donors.

Characteristic	COVID-19 convalescent donors
Donors, n	14
Gender, n (%)	
Male	4 (71%)
Female	10 (29%)
Age, years (mean ± SD)	34.5 ± 16.53
Peak disease severity[a] (%)	
Mild	100%
Race or ethnicity, n (%)[b]	
Asian	0
White	13 (93%)
Hispanic or latino	1 (7%)
SARS-CoV-2 PCR positivity, n (%)	
Positive	14 (100%)
Negative	0
Days PSO at collection, Median (range)	181 (170-195)

PSO, post-symptom onset.

[a]COVID-19 disease severity was defined based on the NIH ordinal scale as previously described (*46*).

[b]Race or ethnic group could include more than one category per donor.

Table S5. Characteristics of 100-μg mRNA-1273 vaccinees.

Characteristic		100-μg mRNA-1273 vaccinees
Donors, n		20
Gender, n (%)		
	Male	6 (30%)
	Female	14 (70%)
Age, year, median (range)		45.5 (36-81)
Race or ethnicity, n (%)[a]		
	Asian	4 (20%)
	White	12 (60%)
	Hispanic or Latino	4 (20%)
	Unknown	0
Days at collection, median (range)		
	Post-vaccination after first immunization[b]	42 (32-55)

[a]Race or ethnic group could include more than one category per donor.

[b]Individuals received the second dose of 100-μg mRNA-1273 15 days before collection.

Table S6. Reagents used for AIM assays.

Reagent	Clone (Source)	Catalog. No.	Dilution
Live/Dead Blue	(ThermoFisher)	L23105	1:1000
CCR6-BUV496	11A9 (BD Biosciences)	612948	1:200
CXCR5-BV421	J252D4 (Biolegend)	356920	1:200
CXCR3-BV605	G025H7 (Biolegend)	353728	1:200
CCR7-BV711	G043H7 (Biolegend)	353228	1:200
CCR4-PE-Cy7	L291H4 (Biolegend)	359410	1:1000
CD3-BUV395	UCHT1 (BD Biosciences)	563546	1:1000
ICOS-BUV563	DX29 (BD Biosciences)	741421	1:200
CD137-BUV737	4B4-1 (BD Biosciences)	741861	1:100
CD8-BUV805	SK1 (BD Biosciences)	612889	1:1000
CD16-BV510	3G8 (Biolegend)	302048	1:1000
CD14-BV510	63D3 (Biolegend)	367124	1:1000
CD20-BV510	2H7 (Biolegend)	302340	1:1000
CD45RA-BV570	HI100 (Biolegend)	304132	1:1000
CD38-BV650	HB-7 (Biolegend)	356620	1:200
PD-1-BV785	EH12.2H7 (Biolegend)	329930	1:200
CD69-FITC	FN50 (Biolegend)	310904	1:200
CD4-cFluor b548	SK3 (Cytek Biosciences)	R7-20043	1:500
CD95-BB700	DX2 (BD Biosciences)	566542	1:500
PDL-1-PE	29E.2A3 (Biolegend)	329706	1:1000
CD40L-PE-Dazzle594	24-31 (Biolegend)	310840	1:200
OX40-APC	Ber-Act35 (Biolegend)	350008	1:100
HLA-DR-APC-R700	G46-6 (BD Biosciences)	565127	1:500
CD25-APC-Fire 750	BC96 (Biolegend)	302642	1:100

Table S7. Additional cohort of COVID-19 convalescent donors and 100-μg mRNA-1273 vaccinees included in the ICS assay[a].

Characteristic	COVID-19 convalescent donors	100-μg mRNA-1273 vaccinees
Donors, n	4	4
Gender, n (%)		
Male	2 (50%)	0
Female	2 (50%)	4 (100%)
Age, year, median (range)	51 (37-67)	49.5 (41-59)
Race or ethnicity, n (%)[b]		
Asian	0	2 (50%)
White	3 (75%)	2 (50%)
Hispanic or Latino	0	0
Unknown	1 (25%)	0
SARS-CoV-2 PCR or anti-spike IgG positivity, n (%)		
Positive	4 (100%)	-
Negative	0	-
Days at collection, median (range)		
Post-symptom onset	91 (47-136)	-
Post-vaccination after first immunization[c]	-	41 (40-47)

[a]See fig. S4 for additional information.

[b]Race or ethnic group could include more than one category per donor.

[c]Individuals received the second dose of 100-μg mRNA-1273 15 days before collection.

Table S8. Reagents used for ICS assays.

Reagent	Clone (Source)	Catalog. No.	Dilution
Live/Dead Blue	(ThermoFisher)	L23105	1:1000
CD3-BUV395	UCHT1 (BD Biosciences)	563546	1:100
CD8-BUV805	SK1 (BD Biosciences)	612889	1:100
CD16-BV510	3G8 (Biolegend)	302048	1:200
CD14-BV510	63D3 (Biolegend)	367124	1:200
CD20-BV510	2H7 (Biolegend)	302340	1:200
CD45RA-BV570	HI100 (Biolegend)	304132	1:50
CD4-cFluor b548	SK3 (Cytek Biosciences)	R7-20043	1:25
CD95-PE-Cy5	DX2 (BD Biosciences)	559773	1:50
CCR7-PE-Cy7	G043H7 (Biolegend)	353226	1:100
IL-4-BUV737	MP4-25D2 (BD Biosciences)	612835	1:200
IL-17-BV785	BL168 (Biolegend)	512338	1:100
IFNγ-FITC	4S.B3 (ThermoFisher)	11-7319-82	1:500
IL-2-BB700	MQ1-17H12 (BD Biosciences)	566405	1:200
IL-10 -PE-Dazzle594	JES3-19F1 (Biolegend)	506812	1:100
TNFα-eFluor450	Mab11 (ThermoFisher)	48-7349-42	1:200
Granzyme B-AF647	GB11 (BD Biosciences)	560212	1:50
CD40L-PerCP-ef710	24-31 (ThermoFisher)	46-1548-42	1:50

References and Notes

1. F. P. Polack, S. J. Thomas, N. Kitchin, J. Absalon, A. Gurtman, S. Lockhart, J. L. Perez, G. Pérez Marc, E. D. Moreira, C. Zerbini, R. Bailey, K. A. Swanson, S. Roychoudhury, K. Koury, P. Li, W. V. Kalina, D. Cooper, R. W. Frenck Jr., L. L. Hammitt, Ö. Türeci, H. Nell, A. Schaefer, S. Ünal, D. B. Tresnan, S. Mather, P. R. Dormitzer, U. Şahin, K. U. Jansen, W. C. Gruber; C4591001 Clinical Trial Group, Safety and efficacy of the BNT162b2 mRNA Covid-19 vaccine. *N. Engl. J. Med.* **383**, 2603–2615 (2020). doi:10.1056/NEJMoa2034577 Medline

2. L. R. Baden, H. M. El Sahly, B. Essink, K. Kotloff, S. Frey, R. Novak, D. Diemert, S. A. Spector, N. Rouphael, C. B. Creech, J. McGettigan, S. Khetan, N. Segall, J. Solis, A. Brosz, C. Fierro, H. Schwartz, K. Neuzil, L. Corey, P. Gilbert, H. Janes, D. Follmann, M. Marovich, J. Mascola, L. Polakowski, J. Ledgerwood, B. S. Graham, H. Bennett, R. Pajon, C. Knightly, B. Leav, W. Deng, H. Zhou, S. Han, M. Ivarsson, J. Miller, T. Zaks; COVE Study Group, Efficacy and safety of the mRNA-1273 SARS-CoV-2 vaccine. *N. Engl. J. Med.* **384**, 403–416 (2021). doi:10.1056/NEJMoa2035389 Medline

3. R. L. Soiza, C. Scicluna, E. C. Thomson, Efficacy and safety of COVID-19 vaccines in older people. *Age Ageing* **50**, 279–283 (2021). doi:10.1093/ageing/afaa274 Medline

4. C. Pawlowski, P. Lenehan, A. Puranik, V. Agarwal, A. J. Venkatakrishnan, M. J. M. Niesen, J. C. O'Horo, A. Virk, M. D. Swift, A. D. Badley, J. Halamka, V. Soundararajan, FDA-authorized mRNA COVID-19 vaccines are effective per real-world evidence synthesized across a multi-state health system. *Med* **2**, 979–992.e8 (2021). doi:10.1016/j.medj.2021.06.007 Medline

5. S. Amit, G. Regev-Yochay, A. Afek, Y. Kreiss, E. Leshem, Early rate reductions of SARS-CoV-2 infection and COVID-19 in BNT162b2 vaccine recipients. *Lancet* **397**, 875–877 (2021). doi:10.1016/S0140-6736(21)00448-7 Medline

6. R. W. Frenck Jr., N. P. Klein, N. Kitchin, A. Gurtman, J. Absalon, S. Lockhart, J. L. Perez, E. B. Walter, S. Senders, R. Bailey, K. A. Swanson, H. Ma, X. Xu, K. Koury, W. V. Kalina, D. Cooper, T. Jennings, D. M. Brandon, S. J. Thomas, Ö. Türeci, D. B. Tresnan, S. Mather, P. R. Dormitzer, U. Şahin, K. U. Jansen, W. C. Gruber; C4591001 Clinical Trial Group, Safety, immunogenicity, and efficacy of the BNT162b2 Covid-19 vaccine in adolescents. *N. Engl. J. Med.* **385**, 239–250 (2021). doi:10.1056/NEJMoa2107456 Medline

7. V. J. Hall, S. Foulkes, A. Saei, N. Andrews, B. Oguti, A. Charlett, E. Wellington, J. Stowe, N. Gillson, A. Atti, J. Islam, I. Karagiannis, K. Munro, J. Khawam, M. A. Chand, C. S. Brown, M. Ramsay, J. Lopez-Bernal, S. Hopkins; SIREN Study Group, COVID-19 vaccine coverage in health-care workers in England and effectiveness of BNT162b2 mRNA vaccine against infection (SIREN): A prospective, multicentre, cohort study. *Lancet* **397**, 1725–1735 (2021). doi:10.1016/S0140-6736(21)00790-X Medline

8. D. Wrapp, N. Wang, K. S. Corbett, J. A. Goldsmith, C.-L. Hsieh, O. Abiona, B. S. Graham, J. S. McLellan, Cryo-EM structure of the 2019-nCoV spike in the prefusion conformation. *Science* **367**, 1260–1263 (2020). doi:10.1126/science.abb2507 Medline

9. L. A. Jackson, E. J. Anderson, N. G. Rouphael, P. C. Roberts, M. Makhene, R. N. Coler, M. P. McCullough, J. D. Chappell, M. R. Denison, L. J. Stevens, A. J. Pruijssers, A.

McDermott, B. Flach, N. A. Doria-Rose, K. S. Corbett, K. M. Morabito, S. O'Dell, S. D. Schmidt, P. A. Swanson 2nd, M. Padilla, J. R. Mascola, K. M. Neuzil, H. Bennett, W. Sun, E. Peters, M. Makowski, J. Albert, K. Cross, W. Buchanan, R. Pikaart-Tautges, J. E. Ledgerwood, B. S. Graham, J. H. Beigel; mRNA-1273 Study Group, An mRNA vaccine against SARS-CoV-2 — Preliminary report. *N. Engl. J. Med.* **383**, 1920–1931 (2020). doi:10.1056/NEJMoa2022483 Medline

10. S. J. Thomas, E. D. Moreira Jr., N. Kitchin, J. Absalon, A. Gurtman, S. Lockhart, J. L. Perez, G. Pérez Marc, F. P. Polack, C. Zerbini, R. Bailey, K. A. Swanson, X. Xu, S. Roychoudhury, K. Koury, S. Bouguermouh, W. V. Kalina, D. Cooper, R. W. Frenck Jr., L. L. Hammitt, Ö. Türeci, H. Nell, A. Schaefer, S. Ünal, Q. Yang, P. Liberator, D. B. Tresnan, S. Mather, P. R. Dormitzer, U. Şahin, W. C. Gruber, K. U. Jansen; C4591001 Clinical Trial Group, Six month safety and efficacy of the BNT162b2 mRNA COVID-19 vaccine. medRxiv 2021.07.28.21261159 [Preprint]. 28 July 2021. https://doi.org/10.1101/2021.07.28.21261159.

11. Moderna, "Moderna Reports Second Quarter Fiscal Year 2021 Financial Results and Provides Business Updates," press release (5 August 2021); https://investors.modernatx.com/news-releases/news-release-details/moderna-reports-second-quarter-fiscal-year-2021-financial.

12. P. B. Gilbert, D. C. Montefiori, A. McDermott, Y. Fong, D. Benkeser, W. Deng, H. Zhou, C. R. Houchens, K. Martins, L. Jayashankar, F. Castellino, B. Flach, B. C. Lin, S. O'Connell, C. McDanal, A. Eaton, M. Sarzotti-Kelsoe, Y. Lu, C. Yu, B. Borate, L. W. P. van der Laan, N. Hejazi, C. Huynh, J. Miller, H. M. El Sahly, L. R. Baden, M. Baron, L. De La Cruz, C. Gay, S. Kalams, C. F. Kelley, M. Kutner, M. P. Andrasik, J. G. Kublin, L. Corey, K. M. Neuzil, L. N. Carpp, R. Pajon, D. Follmann, R. O. Donis, R. A. Koup, Immune correlates analysis of the mRNA-1273 COVID-19 vaccine efficacy trial. medRxiv 2021.08.09.21261290 [Preprint]. 12 August 2021. https://doi.org/10.1101/2021.08.09.21261290.

13. D. S. Khoury, D. Cromer, A. Reynaldi, T. E. Schlub, A. K. Wheatley, J. A. Juno, K. Subbarao, S. J. Kent, J. A. Triccas, M. P. Davenport, Neutralizing antibody levels are highly predictive of immune protection from symptomatic SARS-CoV-2 infection. *Nat. Med.* **27**, 1205–1211 (2021). doi:10.1038/s41591-021-01377-8 Medline

14. A. Tauzin, M. Nayrac, M. Benlarbi, S. Y. Gong, R. Gasser, G. Beaudoin-Bussières, N. Brassard, A. Laumaea, D. Vézina, J. Prévost, S. P. Anand, C. Bourassa, G. Gendron-Lepage, H. Medjahed, G. Goyette, J. Niessl, O. Tastet, L. Gokool, C. Morrisseau, P. Arlotto, L. Stamatatos, A. T. McGuire, C. Larochelle, P. Uchil, M. Lu, W. Mothes, G. De Serres, S. Moreira, M. Roger, J. Richard, V. Martel-Laferrière, R. Duerr, C. Tremblay, D. E. Kaufmann, A. Finzi, A single dose of the SARS-CoV-2 vaccine BNT162b2 elicits Fc-mediated antibody effector functions and T cell responses. *Cell Host Microbe* **29**, 1137–1150.e6 (2021). doi:10.1016/j.chom.2021.06.001 Medline

15. A. Sette, S. Crotty, Adaptive immunity to SARS-CoV-2 and COVID-19. *Cell* **184**, 861–880 (2021). doi:10.1016/j.cell.2021.01.007 Medline

16. L. J. Abu-Raddad, H. Chemaitelly, P. Coyle, J. A. Malek, A. A. Ahmed, Y. A. Mohamoud, S. Younuskunju, H. H. Ayoub, Z. Al Kanaani, E. Al Kuwari, A. A. Butt, A. Jeremijenko, A. H. Kaleeckal, A. N. Latif, R. M. Shaik, H. F. Abdul Rahim, G. K. Nasrallah, H. M.

Yassine, M. G. Al Kuwari, H. E. Al Romaihi, M. H. Al-Thani, A. Al Khal, R. Bertollini, SARS-CoV-2 antibody-positivity protects against reinfection for at least seven months with 95% efficacy. *EClinicalMedicine* **35**, 100861 (2021). doi:10.1016/j.eclinm.2021.100861 Medline

17. S. F. Lumley, D. O'Donnell, N. E. Stoesser, P. C. Matthews, A. Howarth, S. B. Hatch, B. D. Marsden, S. Cox, T. James, F. Warren, L. J. Peck, T. G. Ritter, Z. de Toledo, L. Warren, D. Axten, R. J. Cornall, E. Y. Jones, D. I. Stuart, G. Screaton, D. Ebner, S. Hoosdally, M. Chand, D. W. Crook, A.-M. O'Donnell, C. P. Conlon, K. B. Pouwels, A. S. Walker, T. E. A. Peto, S. Hopkins, T. M. Walker, K. Jeffery, D. W. Eyre; Oxford University Hospitals Staff Testing Group, Antibody status and incidence of SARS-CoV-2 infection in health care workers. *N. Engl. J. Med.* **384**, 533–540 (2021). doi:10.1056/NEJMoa2034545 Medline

18. V. J. Hall, S. Foulkes, A. Charlett, A. Atti, E. J. M. Monk, R. Simmons, E. Wellington, M. J. Cole, A. Saei, B. Oguti, K. Munro, S. Wallace, P. D. Kirwan, M. Shrotri, A. Vusirikala, S. Rokadiya, M. Kall, M. Zambon, M. Ramsay, T. Brooks, C. S. Brown, M. A. Chand, S. Hopkins,; SIREN Study Group, SARS-CoV-2 infection rates of antibody-positive compared with antibody-negative health-care workers in England: A large, multicentre, prospective cohort study (SIREN). *Lancet* **397**, 1459–1469 (2021). doi:10.1016/S0140-6736(21)00675-9 Medline

19. A. Leidi, F. Koegler, R. Dumont, R. Dubos, M. E. Zaballa, G. Piumatti, M. Coen, A. Berner, P. Darbellay Farhoumand, P. Vetter, N. Vuilleumier, L. Kaiser, D. Courvoisier, A. S. Azman, I. Guessous, S. Stringhini; SEROCoV-POP study group, Risk of reinfection after seroconversion to SARS-CoV-2: A population-based propensity-score matched cohort study. *Clin. Infect. Dis.* ciab495 (2021). doi:10.1093/cid/ciab495 Medline

20. N. R. Faria, T. A. Mellan, C. Whittaker, I. M. Claro, D. D. S. Candido, S. Mishra, M. A. E. Crispim, F. C. S. Sales, I. Hawryluk, J. T. McCrone, R. J. G. Hulswit, L. A. M. Franco, M. S. Ramundo, J. G. de Jesus, P. S. Andrade, T. M. Coletti, G. M. Ferreira, C. A. M. Silva, E. R. Manuli, R. H. M. Pereira, P. S. Peixoto, M. U. G. Kraemer, N. Gaburo Jr., C. D. C. Camilo, H. Hoeltgebaum, W. M. Souza, E. C. Rocha, L. M. de Souza, M. C. de Pinho, L. J. T. Araujo, F. S. V. Malta, A. B. de Lima, J. D. P. Silva, D. A. G. Zauli, A. C. S. Ferreira, R. P. Schnekenberg, D. J. Laydon, P. G. T. Walker, H. M. Schlüter, A. L. P. Dos Santos, M. S. Vidal, V. S. Del Caro, R. M. F. Filho, H. M. Dos Santos, R. S. Aguiar, J. L. Proença-Modena, B. Nelson, J. A. Hay, M. Monod, X. Miscouridou, H. Coupland, R. Sonabend, M. Vollmer, A. Gandy, C. A. Prete Jr., V. H. Nascimento, M. A. Suchard, T. A. Bowden, S. L. K. Pond, C.-H. Wu, O. Ratmann, N. M. Ferguson, C. Dye, N. J. Loman, P. Lemey, A. Rambaut, N. A. Fraiji, M. D. P. S. S. Carvalho, O. G. Pybus, S. Flaxman, S. Bhatt, E. C. Sabino, Genomics and epidemiology of the P.1 SARS-CoV-2 lineage in Manaus, Brazil. *Science* **372**, 815–821 (2021). doi:10.1126/science.abh2644 Medline

21. J. Zuo, A. C. Dowell, H. Pearce, K. Verma, H. M. Long, J. Begum, F. Aiano, Z. Amin-Chowdhury, K. Hoschler, T. Brooks, S. Taylor, J. Hewson, B. Hallis, L. Stapley, R. Borrow, E. Linley, S. Ahmad, B. Parker, A. Horsley, G. Amirthalingam, K. Brown, M. E. Ramsay, S. Ladhani, P. Moss, Robust SARS-CoV-2-specific T cell immunity is maintained at 6 months following primary infection. *Nat. Immunol.* **22**, 620–626 (2021). doi:10.1038/s41590-021-00902-8 Medline

22. J. M. Dan, J. Mateus, Y. Kato, K. M. Hastie, E. D. Yu, C. E. Faliti, A. Grifoni, S. I. Ramirez, S. Haupt, A. Frazier, C. Nakao, V. Rayaprolu, S. A. Rawlings, B. Peters, F. Krammer, V. Simon, E. O. Saphire, D. M. Smith, D. Weiskopf, A. Sette, S. Crotty, Immunological memory to SARS-CoV-2 assessed for up to 8 months after infection. *Science* **371**, eabf4063 (2021). doi:10.1126/science.abf4063 Medline

23. R. J. Boyton, D. M. Altmann, Risk of SARS-CoV-2 reinfection after natural infection. *Lancet* **397**, 1161–1163 (2021). doi:10.1016/S0140-6736(21)00662-0 Medline

24. C. Li, D. Yu, X. Wu, H. Liang, Z. Zhou, Y. Xie, T. Li, J. Wu, F. Lu, L. Feng, M. Mao, L. Lin, H. Guo, S. Yue, F. Wang, Y. Peng, Y. Hu, Z. Wang, J. Yu, Y. Zhang, J. Lu, H. Ning, H. Yang, D. Fu, Y. He, D. Zhou, T. Du, K. Duan, D. Dong, K. Deng, X. Zou, Y. Zhang, R. Zhou, Y. Gao, X. Zhang, X. Yang, Twelve-month specific IgG response to SARS-CoV-2 receptor-binding domain among COVID-19 convalescent plasma donors in Wuhan. *Nat. Commun.* **12**, 4144 (2021). doi:10.1038/s41467-021-24230-5 Medline

25. Z. Wang, F. Muecksch, D. Schaefer-Babajew, S. Finkin, C. Viant, C. Gaebler, H.-H. Hoffmann, C. O. Barnes, M. Cipolla, V. Ramos, T. Y. Oliveira, A. Cho, F. Schmidt, J. Da Silva, E. Bednarski, L. Aguado, J. Yee, M. Daga, M. Turroja, K. G. Millard, M. Jankovic, A. Gazumyan, Z. Zhao, C. M. Rice, P. D. Bieniasz, M. Caskey, T. Hatziioannou, M. C. Nussenzweig, Naturally enhanced neutralizing breadth against SARS-CoV-2 one year after infection. *Nature* **595**, 426–431 (2021). doi:10.1038/s41586-021-03696-9 Medline

26. K. W. Cohen, S. L. Linderman, Z. Moodie, J. Czartoski, L. Lai, G. Mantus, C. Norwood, L. E. Nyhoff, V. V. Edara, K. Floyd, S. C. De Rosa, H. Ahmed, R. Whaley, S. N. Patel, B. Prigmore, M. P. Lemos, C. W. Davis, S. Furth, J. B. O'Keefe, M. P. Gharpure, S. Gunisetty, K. Stephens, R. Antia, V. I. Zarnitsyna, D. S. Stephens, S. Edupuganti, N. Rouphael, E. J. Anderson, A. K. Mehta, J. Wrammert, M. S. Suthar, R. Ahmed, M. J. McElrath, Longitudinal analysis shows durable and broad immune memory after SARS-CoV-2 infection with persisting antibody responses and memory B and T cells. *Cell Rep. Med.* **2**, 100354 (2021). doi:10.1016/j.xcrm.2021.100354 Medline

27. A. Pegu, S. O'Connell, S. D. Schmidt, S. O'Dell, C. A. Talana, L. Lai, J. Albert, E. Anderson, H. Bennett, K. S. Corbett, B. Flach, L. Jackson, B. Leav, J. E. Ledgerwood, C. J. Luke, M. Makowski, M. C. Nason, P. C. Roberts, M. Roederer, P. A. Rebolledo, C. A. Rostad, N. G. Rouphael, W. Shi, L. Wang, A. T. Widge, E. S. Yang, J. H. Beigel, B. S. Graham, J. R. Mascola, M. S. Suthar, A. B. McDermott, N. A. Doria-Rose; mRNA-1273 Study Group, Durability of mRNA-1273 vaccine-induced antibodies against SARS-CoV-2 variants. *Science* eabj4176 (2021). doi:10.1126/science.abj4176 Medline

28. E. J. Anderson, N. G. Rouphael, A. T. Widge, L. A. Jackson, P. C. Roberts, M. Makhene, J. D. Chappell, M. R. Denison, L. J. Stevens, A. J. Pruijssers, A. B. McDermott, B. Flach, B. C. Lin, N. A. Doria-Rose, S. O'Dell, S. D. Schmidt, K. S. Corbett, P. A. Swanson 2nd, M. Padilla, K. M. Neuzil, H. Bennett, B. Leav, M. Makowski, J. Albert, K. Cross, V. V. Edara, K. Floyd, M. S. Suthar, D. R. Martinez, R. Baric, W. Buchanan, C. J. Luke, V. K. Phadke, C. A. Rostad, J. E. Ledgerwood, B. S. Graham, J. H. Beigel; mRNA-1273 Study Group, Safety and immunogenicity of SARS-CoV-2 mRNA-1273 vaccine in older adults. *N. Engl. J. Med.* **383**, 2427–2438 (2020). doi:10.1056/NEJMoa2028436 Medline

29. US Food and Drug Administration, "Vaccines and Related Biological Products Advisory Committee Meeting Presentation," 22 October 2020.

30. J. Braun, L. Loyal, M. Frentsch, D. Wendisch, P. Georg, F. Kurth, S. Hippenstiel, M. Dingeldey, B. Kruse, F. Fauchere, E. Baysal, M. Mangold, L. Henze, R. Lauster, M. A. Mall, K. Beyer, J. Röhmel, S. Voigt, J. Schmitz, S. Miltenyi, I. Demuth, M. A. Müller, A. Hocke, M. Witzenrath, N. Suttorp, F. Kern, U. Reimer, H. Wenschuh, C. Drosten, V. M. Corman, C. Giesecke-Thiel, L. E. Sander, A. Thiel, SARS-CoV-2-reactive T cells in healthy donors and patients with COVID-19. *Nature* **587**, 270–274 (2020). doi:10.1038/s41586-020-2598-9 Medline

31. A. Grifoni, D. Weiskopf, S. I. Ramirez, J. Mateus, J. M. Dan, C. R. Moderbacher, S. A. Rawlings, A. Sutherland, L. Premkumar, R. S. Jadi, D. Marrama, A. M. de Silva, A. Frazier, A. F. Carlin, J. A. Greenbaum, B. Peters, F. Krammer, D. M. Smith, S. Crotty, A. Sette, Targets of T cell responses to SARS-CoV-2 coronavirus in humans with COVID-19 disease and unexposed individuals. *Cell* **181**, 1489–1501.e15 (2020). doi:10.1016/j.cell.2020.05.015 Medline

32. N. Le Bert, A. T. Tan, K. Kunasegaran, C. Y. L. Tham, M. Hafezi, A. Chia, M. H. Y. Chng, M. Lin, N. Tan, M. Linster, W. N. Chia, M. I.-C. Chen, L.-F. Wang, E. E. Ooi, S. Kalimuddin, P. A. Tambyah, J. G.-H. Low, Y.-J. Tan, A. Bertoletti, SARS-CoV-2-specific T cell immunity in cases of COVID-19 and SARS, and uninfected controls. *Nature* **584**, 457–462 (2020). doi:10.1038/s41586-020-2550-z Medline

33. M. Lipsitch, Y. H. Grad, A. Sette, S. Crotty, Cross-reactive memory T cells and herd immunity to SARS-CoV-2. *Nat. Rev. Immunol.* **20**, 709–713 (2020). doi:10.1038/s41577-020-00460-4 Medline

34. J. Mateus, A. Grifoni, A. Tarke, J. Sidney, S. I. Ramirez, J. M. Dan, Z. C. Burger, S. A. Rawlings, D. M. Smith, E. Phillips, S. Mallal, M. Lammers, P. Rubiro, L. Quiambao, A. Sutherland, E. D. Yu, R. da Silva Antunes, J. Greenbaum, A. Frazier, A. J. Markmann, L. Premkumar, A. de Silva, B. Peters, S. Crotty, A. Sette, D. Weiskopf, Selective and cross-reactive SARS-CoV-2 T cell epitopes in unexposed humans. *Science* **370**, 89–94 (2020). doi:10.1126/science.abd3871 Medline

35. B. J. Meckiff, C. Ramírez-Suástegui, V. Fajardo, S. J. Chee, A. Kusnadi, H. Simon, S. Eschweiler, A. Grifoni, E. Pelosi, D. Weiskopf, A. Sette, F. Ay, G. Seumois, C. H. Ottensmeier, P. Vijayanand, Imbalance of regulatory and cytotoxic SARS-CoV-2-reactive $CD4^+$ T cells in COVID-19. *Cell* **183**, 1340–1353.e16 (2020). doi:10.1016/j.cell.2020.10.001 Medline

36. A. Sette, S. Crotty, Pre-existing immunity to SARS-CoV-2: The knowns and unknowns. *Nat. Rev. Immunol.* **20**, 457–458 (2020). doi:10.1038/s41577-020-0389-z Medline

37. D. Weiskopf, K. S. Schmitz, M. P. Raadsen, A. Grifoni, N. M. A. Okba, H. Endeman, J. P. C. van den Akker, R. Molenkamp, M. P. G. Koopmans, E. C. M. van Gorp, B. L. Haagmans, R. L. de Swart, A. Sette, R. D. de Vries, Phenotype and kinetics of SARS-CoV-2-specific T cells in COVID-19 patients with acute respiratory distress syndrome. *Sci. Immunol.* **5**, eabd2071 (2020). doi:10.1126/sciimmunol.abd2071 Medline

38. E. M. Anderson, E. C. Goodwin, A. Verma, C. P. Arevalo, M. J. Bolton, M. E. Weirick, S. Gouma, C. M. McAllister, S. R. Christensen, J. Weaver, P. Hicks, T. B. Manzoni, O. Oniyide, H. Ramage, D. Mathew, A. E. Baxter, D. A. Oldridge, A. R. Greenplate, J. E.

Wu, C. Alanio, K. D'Andrea, O. Kuthuru, J. Dougherty, A. Pattekar, J. Kim, N. Han, S. A. Apostolidis, A. C. Huang, L. A. Vella, L. Kuri-Cervantes, M. B. Pampena, M. R. Betts, E. J. Wherry, N. J. Meyer, S. Cherry, P. Bates, D. J. Rader, S. E. Hensley; UPenn COVID Processing Unit, Seasonal human coronavirus antibodies are boosted upon SARS-CoV-2 infection but not associated with protection. *Cell* **184**, 1858–1864.e10 (2021). doi:10.1016/j.cell.2021.02.010 Medline

39. M. Sagar, K. Reifler, M. Rossi, N. S. Miller, P. Sinha, L. F. White, J. P. Mizgerd, Recent endemic coronavirus infection is associated with less-severe COVID-19. *J. Clin. Invest.* **131**, e143380 (2021). doi:10.1172/JCI143380 Medline

40. N. Doria-Rose, M. S. Suthar, M. Makowski, S. O'Connell, A. B. McDermott, B. Flach, J. E. Ledgerwood, J. R. Mascola, B. S. Graham, B. C. Lin, S. O'Dell, S. D. Schmidt, A. T. Widge, V.-V. Edara, E. J. Anderson, L. Lai, K. Floyd, N. G. Rouphael, V. Zarnitsyna, P. C. Roberts, M. Makhene, W. Buchanan, C. J. Luke, J. H. Beigel, L. A. Jackson, K. M. Neuzil, H. Bennett, B. Leav, J. Albert, P. Kunwar; mRNA-1273 Study Group, Antibody persistence through 6 months after the second dose of mRNA-1273 vaccine for Covid-19. *N. Engl. J. Med.* **384**, 2259–2261 (2021). doi:10.1056/NEJMc2103916 Medline

41. S. Crotty, T follicular helper cell biology: A decade of discovery and diseases. *Immunity* **50**, 1132–1148 (2019). doi:10.1016/j.immuni.2019.04.011 Medline

42. C. E. Gustafson, C. Kim, C. M. Weyand, J. J. Goronzy, Influence of immune aging on vaccine responses. *J. Allergy Clin. Immunol.* **145**, 1309–1321 (2020). doi:10.1016/j.jaci.2020.03.017 Medline

43. A. Grifoni, J. Sidney, Y. Zhang, R. H. Scheuermann, B. Peters, A. Sette, A sequence homology and bioinformatic approach can predict candidate targets for immune responses to SARS-CoV-2. *Cell Host Microbe* **27**, 671–680.e2 (2020). doi:10.1016/j.chom.2020.03.002 Medline

44. U. Sahin, A. Muik, I. Vogler, E. Derhovanessian, L. M. Kranz, M. Vormehr, J. Quandt, N. Bidmon, A. Ulges, A. Baum, K. E. Pascal, D. Maurus, S. Brachtendorf, V. Lörks, J. Sikorski, P. Koch, R. Hilker, D. Becker, A.-K. Eller, J. Grützner, M. Tonigold, C. Boesler, C. Rosenbaum, L. Heesen, M.-C. Kühnle, A. Poran, J. Z. Dong, U. Luxemburger, A. Kemmer-Brück, D. Langer, M. Bexon, S. Bolte, T. Palanche, A. Schultz, S. Baumann, A. J. Mahiny, G. Boros, J. Reinholz, G. T. Szabó, K. Karikó, P.-Y. Shi, C. Fontes-Garfias, J. L. Perez, M. Cutler, D. Cooper, C. A. Kyratsous, P. R. Dormitzer, K. U. Jansen, Ö. Türeci, BNT162b2 vaccine induces neutralizing antibodies and poly-specific T cells in humans. *Nature* **595**, 572–577 (2021). doi:10.1038/s41586-021-03653-6 Medline

45. K. S. Corbett, B. Flynn, K. E. Foulds, J. R. Francica, S. Boyoglu-Barnum, A. P. Werner, B. Flach, S. O'Connell, K. W. Bock, M. Minai, B. M. Nagata, H. Andersen, D. R. Martinez, A. T. Noe, N. Douek, M. M. Donaldson, N. N. Nji, G. S. Alvarado, D. K. Edwards, D. R. Flebbe, E. Lamb, N. A. Doria-Rose, B. C. Lin, M. K. Louder, S. O'Dell, S. D. Schmidt, E. Phung, L. A. Chang, C. Yap, J. M. Todd, L. Pessaint, A. Van Ry, S. Browne, J. Greenhouse, T. Putman-Taylor, A. Strasbaugh, T.-A. Campbell, A. Cook, A. Dodson, K. Steingrebe, W. Shi, Y. Zhang, O. M. Abiona, L. Wang, A. Pegu, E. S. Yang, K. Leung, T. Zhou, I.-T. Teng, A. Widge, I. Gordon, L. Novik, R. A. Gillespie, R. J. Loomis, J. I. Moliva, G. Stewart-Jones, S. Himansu, W.-P. Kong, M. C. Nason, K. M. Morabito, T. J.

Ruckwardt, J. E. Ledgerwood, M. R. Gaudinski, P. D. Kwong, J. R. Mascola, A. Carfi, M. G. Lewis, R. S. Baric, A. McDermott, I. N. Moore, N. J. Sullivan, M. Roederer, R. A. Seder, B. S. Graham, Evaluation of the mRNA-1273 vaccine against SARS-CoV-2 in nonhuman primates. *N. Engl. J. Med.* **383**, 1544–1555 (2020). doi:10.1056/NEJMoa2024671 Medline

46. C. Rydyznski Moderbacher, S. I. Ramirez, J. M. Dan, A. Grifoni, K. M. Hastie, D. Weiskopf, S. Belanger, R. K. Abbott, C. Kim, J. Choi, Y. Kato, E. G. Crotty, C. Kim, S. A. Rawlings, J. Mateus, L. P. V. Tse, A. Frazier, R. Baric, B. Peters, J. Greenbaum, E. Ollmann Saphire, D. M. Smith, A. Sette, S. Crotty, Antigen-specific adaptive immunity to SARS-CoV-2 in acute COVID-19 and associations with age and disease severity. *Cell* **183**, 996–1012.e19 (2020). doi:10.1016/j.cell.2020.09.038 Medline

47. A. T. Tan, M. Linster, C. W. Tan, N. Le Bert, W. N. Chia, K. Kunasegaran, Y. Zhuang, C. Y. L. Tham, A. Chia, G. J. D. Smith, B. Young, S. Kalimuddin, J. G. H. Low, D. Lye, L.-F. Wang, A. Bertoletti, Early induction of functional SARS-CoV-2-specific T cells associates with rapid viral clearance and mild disease in COVID-19 patients. *Cell Rep.* **34**, 108728 (2021). doi:10.1016/j.celrep.2021.108728 Medline

48. S. A. Lauer, K. H. Grantz, Q. Bi, F. K. Jones, Q. Zheng, H. R. Meredith, A. S. Azman, N. G. Reich, J. Lessler, The incubation period of coronavirus disease 2019 (COVID-19) from publicly reported confirmed cases: Estimation and application. *Ann. Intern. Med.* **172**, 577–582 (2020). doi:10.7326/M20-0504 Medline

49. F. Amanat, D. Stadlbauer, S. Strohmeier, T. H. O. Nguyen, V. Chromikova, M. McMahon, K. Jiang, G. A. Arunkumar, D. Jurczyszak, J. Polanco, M. Bermudez-Gonzalez, G. Kleiner, T. Aydillo, L. Miorin, D. S. Fierer, L. A. Lugo, E. M. Kojic, J. Stoever, S. T. H. Liu, C. Cunningham-Rundles, P. L. Felgner, T. Moran, A. García-Sastre, D. Caplivski, A. C. Cheng, K. Kedzierska, O. Vapalahti, J. M. Hepojoki, V. Simon, F. Krammer, A serological assay to detect SARS-CoV-2 seroconversion in humans. *Nat. Med.* **26**, 1033–1036 (2020). doi:10.1038/s41591-020-0913-5 Medline

50. S. Carrasco Pro, J. Sidney, S. Paul, C. Lindestam Arlehamn, D. Weiskopf, B. Peters, A. Sette, Automatic generation of validated specific epitope sets. *J. Immunol. Res.* **2015**, 763461 (2015). doi:10.1155/2015/763461 Medline

51. M. F. Kotturi, B. Peters, F. Buendia-Laysa Jr., J. Sidney, C. Oseroff, J. Botten, H. Grey, M. J. Buchmeier, A. Sette, The CD8^{+} T-cell response to lymphocytic choriomeningitis virus involves the L antigen: Uncovering new tricks for an old virus. *J. Virol.* **81**, 4928–4940 (2007). doi:10.1128/JVI.02632-06 Medline

52. A. Grifoni, J. Sidney, R. Vita, B. Peters, S. Crotty, D. Weiskopf, A. Sette, SARS-CoV-2 human T cell epitopes: Adaptive immune response against COVID-19. *Cell Host Microbe* **29**, 1076–1092 (2021). doi:10.1016/j.chom.2021.05.010 Medline
