# Supplementary material for: Low-dose mRNA-1273 COVID-19 vaccine generates durable memory enhanced by cross-reactive T cells
Source: Science. Author manuscript; Available in PMC 2021 Oct 25. (PMC8542617; doi:10.1126/science.abj9853)
Supplement: Supplementary file 2 [file NIHMS1743942-supplement-data_caption.docx]

Data S1. Spike-specific antibody and T cell data in low-dose vaccines (.xlsx file).
